# The Affordable Care Act Medicaid expansion: A difference-in-differences study of spillover participation in SNAP

**DOI:** 10.1371/journal.pone.0267244

**Published:** 2022-05-04

**Authors:** Paulette Cha, José J. Escarce

**Affiliations:** 1 Department of Health Policy and Management, UCLA Fielding School of Public Health, Los Angeles, California, United States of America; 2 Schaeffer Center for Health Policy and Economics, University of Southern California, Los Angeles, California, United States of America; 3 UC Berkeley, Institute of Government Studies, Berkeley, CA; 4 Division of General Internal Medicine, UCLA Geffen School of Medicine, Los Angeles, California, United States of America; Drexel University, UNITED STATES

## Abstract

The Affordable Care Act’s Medicaid expansion to individuals with adults under 138 percent of the federal poverty level led to insurance coverage for millions of Americans in participating states. This study investigates Medicaid expansion’s potential spillover participation in the Supplemental Nutrition Assistance Program (SNAP; formerly the Food Stamp Program). In addition to providing public insurance, the policy connects individuals to SNAP, affecting social determinants of health such as hunger. We use difference-in-differences regression to estimate the effect of the Medicaid expansion on SNAP participation among approximately 414,000 individuals from across the United States. The Current Population Survey is used to answer the main research question, and the SNAP Quality Control Database allows for supplemental analyses. Medicaid expansion produces a 2.9 percentage point increase (p = 0.002) in SNAP participation among individuals under 138 percent of federal poverty. Subgroup analyses find a larger 5.0 percentage point increase (p = 0.002) in households under 75 percent of federal poverty without children. Able-Bodied Adults Without Dependents (ABAWDs) are a category of individuals with limited access to SNAP. Although they are a subset of adults without children, we found no spillover effect for ABAWDs. We find an increase in SNAP households with $0 income, supporting the finding that spillover was strongest for very-low-income individuals. Joint processing of Medicaid and SNAP applications helps facilitate the connection between Medicaid expansion and SNAP. Our findings contribute to a growing body of evidence that Medicaid expansion does more than improve access to health care by connecting eligible individuals to supports like SNAP. SNAP recipients have increased access to food, an important social determinant of health. Our study supports reducing administrative burdens to help connect individuals to safety net programs. Finally, we note that ABAWDs are a vulnerable group that need targeted program outreach.

## Introduction

The Affordable Care Act (ACA) Medicaid expansion is one of the most significant recent public investments in health insurance. The ACA was designed as a comprehensive reform of health care in the United States, and one of its priorities was to reduce uninsurance. Medicaid, the primary public health insurance program for low-income individuals, was previously only available in many states to children and limited categories of adults such as the elderly, disabled individuals, and pregnant women. The ACA authorized an expansion of Medicaid, and mandated states to begin enrolling adults under 138 percent of the federal poverty level (FPL) in the program by 2014. Unlike past Medicaid provisions for adults, the ACA expansion had no categorical requirement such as needing to be a parent or have a disability. A Supreme Court ruling in 2012, however, recast the mandate as a state option. Twenty-five states, including the District of Columbia, implemented the expansion when it first became available in January 2014. Others have since joined and there are 39 participating states at the time of writing [1].

Medicaid expansion produced reductions in uninsurance that exceed improvements in non-expansion states [2–4], but research is finding that its effects go beyond improving coverage. The expansion increased access to and use of health care, even among hard-to-reach subpopulations [3–5]. Studies find that the expansion led to improvements in self-reported health [6], reduced hospital readmissions [7], and reduced mortality rates [8]. It also made health care more affordable and improved financial security [9, 10]. The ACA expansion is such a large shift in social policy that it had the potential to produce spillover effects that go beyond the widely known “welcome mat” enrollments of already-eligible individuals into Medicaid [11]. An important question in an evaluation of the Medicaid expansion is whether it had effects on health or well-being through enrollment in other social safety net programs.

## The Supplemental Nutrition Assistance Program (SNAP)

SNAP (formerly the Food Stamp Program) is a federal program that provides monthly food purchasing assistance to improve the nutrition of low-income households [12]. The program is disbursed through Electronic Benefit Transfer (EBT) cards, which function like debit cards. Eligibility and benefit amounts are determined at the household level, unlike individual programs such as Medicaid. Households that are larger or have lower incomes are eligible for greater benefit amounts. The program has also seen a range of expansions and cutbacks over time in response to political and economic changes [13]. Reform of the welfare system in 1996 reduced the role of cash welfare in protecting families against poverty; in its place, SNAP has emerged as one of the largest and most effective anti-poverty programs [14, 15], especially during recessionary periods [16].

A review of the literature on social determinants highlights SNAP and other nutritional supports as investments that can improve health outcomes [17]. SNAP has been found to significantly reduce child food insecurity and improve child health [18]. Research has found that *in utero* exposure to the program improved birth outcomes and reduced infant mortality [19]. Children exposed to the program have a reduced incidence of metabolic syndrome as adults, suggesting there are long-run health effects [20]. The program also improves diet quality and can reduce the likelihood of obesity among adults [21]. However, like all public programs, there are a variety of reasons eligible individuals do not participate [22, 23].

### Why Medicaid might affect SNAP

Barriers to participation in programs such as Medicaid and SNAP include transaction costs, stigma, and poor access to information. Transaction costs include collecting documentation and traveling to an agency to apply [24]. Administrative complexity was found to be an especially important barrier to enrolling children of Latino and Asian backgrounds [25], reflecting a need for culturally and linguistically appropriate outreach. Two randomized social experiments have found that assistance with applying improves SNAP participation [26, 27], showing that reducing transaction costs is an effective strategy to increase enrollment. When the potential benefit is high enough, overcoming transaction costs is more worthwhile. A study of low-income immigrant families found that having a greater number of eligible children predicted a higher likelihood of Medicaid enrollment [28], suggesting that transaction costs play a role separate from stigma.

Stigma associated with programs may result in a situation where some eligible people prefer not to participate [29]. Experimental evidence finds that outreach materials highlighting a lack of stigma improves individuals’ response to SNAP outreach relative to standard materials [27]. Finally, having poor access to information could mean that eligible individuals have not heard of a program at all, or do not know that they qualify. We know from past research that some individuals are more likely to overcome information barriers. An early experimental study found, for example, that the lowest-income individuals—who stood to receive large benefit amounts reflecting their greater need—were more likely to have heard of SNAP [30]. Simply providing information increased SNAP enrollment among older adults with Medicaid relative to a control group in a randomized study [26].

Income eligibility for Medicaid and SNAP had significant overlap prior to the ACA, especially for parents, and the overlap stood to grow for childless adults in Medicaid expansion states [31]. Under the ACA expansion, Medicaid covers nearly all adults up to 138 percent of federal poverty, which is similar to the SNAP income limit. States can be more generous with eligibility criteria, but at a minimum SNAP covers households up to 130 percent of FPL that meet additional requirements [32]. The two programs target similar populations in terms of income, and the Medicaid expansion had the potential to reduce some barriers to SNAP enrollment.

The Medicaid expansion could have reduced transaction costs associated with applying for SNAP since enrolling in Medicaid could reduce the burden of applying for another program if the offices are located in the same place. The expansion also offers a range of options for states to reduce administrative burden and increase enrollment. Expansion states can train staff in assister roles to facilitate enrollment in Medicaid, and allow rapid pre-screening of eligibility [33]. In terms of aligning Medicaid and SNAP, options include data sharing across the programs to enroll and recertify, and aligning recertification timelines to reduce applicants’ burden [34]. Express Lane measures, which were initially used to automatically or more easily enroll children in public health insurance programs using data from other programs [35], can be employed to enroll adults in Medicaid through an ACA provision [36]. In some states that relied on the federal health insurance marketplace, local organizations deployed ACA healthcare navigators. Navigators are staff funded by federal grants to help enroll individuals into marketplace health insurance even if they have low health insurance literacy or are distrustful of government programs [37]. It is likely that navigators guide eligible low-income adults to Medicaid in expansion states, possibly along with other safety net programs. While not as direct a link between Medicaid and SNAP as Express Lane measures, the navigators would operate in the direction we hypothesize (i.e., Medicaid to SNAP). Finally, in some states, Medicaid and SNAP applications are jointly processed, meaning that there is automatic consideration for SNAP when an individual applies for Medicaid. This, too, could have helped Medicaid expansion connect individuals to SNAP benefits (or the other way around).

The ACA may have reduced the stigma associated with Medicaid. It required individuals to have health insurance through its individual mandate, and authorized the expansion of Medicaid as one of the ways to help people comply with the law. The mandate made exceptions for those who met certain financial requirements (and has since been effectively repealed [38]), but while it was in force, it conveyed the message that health insurance was required by law. These provisions may have reduced Medicaid’s stigma by making participation “mandatory” for those who would otherwise be uninsured. Qualitative research has found that health care navigators did not question individuals’ worthiness for health insurance in the ways documented for SNAP or other programs [39], supporting the idea that stigma associated with health insurance is low in the context of the ACA. Finally, but importantly, uninsured individuals can be enrolled in Medicaid by clinics or hospitals after receiving health services. Providers should be motivated to enroll eligible low-income patients in Medicaid to ensure payment for their services, and their actions both reduce transaction costs and circumvent the issue of stigma for the applicant.

Information costs may also have decreased with the ACA Medicaid expansion.

Medicaid was already one of the largest and most well-known social programs, but the ACA expansion further publicized the program at the national level, and participating states conducted print, radio, and billboard campaigns to further improve the target population’s information about the program [33]. And the additional information cost of learning about another program such as SNAP can decline substantially when there is physical co-location of program offices, or once an applicant has begun to engage with a government employee.

### Related research

The few studies on the relationship between Medicaid expansions and SNAP show there are links between new Medicaid eligibility and spillover effects on SNAP, and provide an important baseline of results to which to compare our findings. Early work by Yelowitz found that Medicaid expansions to children that began in the 1980s led to a 0.22 percentage point (or about 3 percent) increase in SNAP participation [40]. A study by Agirdas focused on several states that expanded Medicaid in the early 2000s to adults, most of whom were childless; comparing more affected counties to less affected ones, the author estimated a 0.49 percent increase in SNAP participation [41]. Baicker et al. found in the Oregon Health Insurance Experiment, which notably targeted adults, being randomly selected for a Medicaid application opportunity produced a 2.5 percentage point (4 percent) increase in SNAP participation [42]. Based on these pre-ACA findings, it is reasonable to investigate the ACA Medicaid expansion for possible effects on SNAP participation.

Researchers have been building evidence that the ACA Medicaid expansion affects SNAP. Lanese et al. showed that the expansion combined with outreach led to about a 10 percent participation increase in SNAP [43]. Burney et al. found increases in SNAP participation, with strong effects for households with very low incomes [44]. Schmidt et al. analyze counties along borders separating ACA expansion states from non-expansion states; their preferred approach finds that expansion leads to about a 0.6 percentage point (4 percent) increase in SNAP [45]. Most of the effects translate to around a 3 to 4 percent increase in SNAP. In the Lanese et al. study, the authors found an (insignificant) effect size under 4 percent for expansion states without outreach that was not statistically distinguishable from their larger estimate for expansion states with outreach, suggesting that their average effect could be closer to the findings in the other studies we describe.

Able-Bodied Adults Without Dependents (ABAWDs) typically have to meet additional work requirements to receive SNAP. This population is of policy interest because safety net programs tend to target working adults or families with children, meaning that ABAWDs who are not working are vulnerable to falling through the cracks even if they have very high need. Households without children are not exactly equivalent to those with ABAWDs; having elder care responsibilities, being 50 years of age or older, or having a disability are examples of reasons a non-working adult would be exempt from the ABAWD work requirements. ABAWDs are not completely barred from SNAP, but their access to the program is very limited. They may access SNAP for three months every three years before reaching a time limit; after that, they could be ineligible for the program even when working [46]. States also have discretion to allow non-working ABAWDs to participate provided they do not make up more than 15 percent of the state’s SNAP caseload [47, 48]. The work requirements were broadly waived in most of the country during the Great Recession because of reduced employment opportunities, with states and localities shedding waivers as their economies improved [49, 50]. One of the key findings in the Burney et al. work is that the effect of Medicaid expansion on SNAP is especially strong for households without children, which they interpret as households made up of ABAWDs. However, identifying ABAWDs in secondary data is difficult because most sources do not have sufficient information to determine whether, for example, an individual has care responsibilities for an elder. Like the Burney et al. study, our project investigates households without children, but we do not assume that members are ABAWDs; instead, we analyze ABAWDs in data where they may be more accurately identified.

Baicker et al., Schmidt et al., Lanese et al., and Burney et al. provide the most relevant context for our work because they evaluate recent Medicaid expansions to adults, a group that historically lacked eligibility unless they met specific categorical requirements. The Oregon experiment used the gold-standard research design of random assignment, but its findings are based on a homogenous population in a single state. The study sample was over 80 percent white and over 80 percent non-Latino, with more than 90 percent indicating English as their preferred language [51]. The border-county study is compared similar communities to each other and covered a broad geography, but large swaths of the US were excluded because they and their neighbors made the same Medicaid expansion decision [45]. This was likely to overrepresent rural communities like those on the New York State border with Pennsylvania while excluding dominant population centers like New York City and all of California.

Our study complements and extends the work of these authors. We endeavor to understand how Medicaid expansion affects SNAP participation, including studying which individuals experience spillover, and how spillover might occur. Like the Lanese et al. and Burney et al. studies, we include all of the US. Lanese et al. produced evidence that the expansion plus outreach was effective in enrolling individuals in SNAP, but their study did not investigate what types of outreach worked, or for which groups. We build on their work by investigating potential mechanisms for connecting individuals to SNAP, and we do so for subgroups. We also extend the Burney et al. work by analyzing ABAWDS, an especially vulnerable group of low-income adults, in a way that distinguishes them among the broader group of adults without children.

## Hypotheses

This study investigates several related hypotheses: first, the ACA Medicaid expansion produces a spillover increase in participation in SNAP and, since benefits are disbursed to households, any spillover participation into SNAP would affect members not targeted by the insurance expansion such as children. Because households with children have more access to safety net programs generally, our second hypothesis is that the presence of children in a household predicts a larger effect. Our third hypothesis is that the spillover effect may be concentrated among the poorest eligible households, who have the greatest need for nutritional assistance and consequently, stand to receive larger benefit amounts from SNAP [30]. Our final hypothesis is that ABAWDs, who have limited access to safety net programs, but who gained eligibility for Medicaid in the expansion, will have large spillover effects; this would be consistent with the Burney et al. findings.

## Methods

### Data

We analyze publicly-available data from the Annual Social and Economic Supplement (ASEC) of the Current Population Survey (CPS) organized by the Integrated Public Use Microdata Series [52]. The CPS is an ongoing series of monthly national surveys of the labor force conducted by the US Census Bureau. The ASEC Supplement contains data on a large, nationally representative sample of households and includes information on sociodemographic characteristics and participation in public programs. It references the previous calendar year in its question about SNAP participation, our outcome of interest. Data from 2011 to 2020 therefore result in an analysis period from 2010 to 2019.

We also estimate supplementary models using publicly-available SNAP Quality Control Database (SNAP QC) to describe changes to characteristics of participating households that are difficult to verify in other data sources [53]. SNAP QC is an administrative dataset containing monthly representative samples of SNAP households submitted by state agencies to the federal government for program oversight. These data include only participating households. We use the SNAP QC to estimate changes to the monthly benefit amount for participants. We also use it to investigate whether there was a change to the types of households participating. We analyze the number of SNAP households with no income at all, and the number with an ABAWD member. We expect that ABAWD households have substantial overlap with the households without children we study in the CPS ASEC, but it is likely more reliable to analyze ABAWDs in the administrative data, where their status is explicitly flagged. We reorganize the SNAP QC fiscal year data from 2010 to 2019 into calendar years 2010 to 2019 (2019 is a partial year).

Our final data source is the Current Population Survey’s December Supplement, which includes monthly SNAP participation [52]. A handful of states—Indiana, Michigan, New Hampshire, Louisiana, and Alaska—expanded Medicaid in months other than January. Studying these states illustrates whether SNAP receipt patterns varied with respect to the expansion timing. We use the December CPS from 2014 to 2016 since only these are the only years in our study period containing mid-year expansions. Documentation from the Kaiser Family Foundation is used to define the presence and timing of ACA Medicaid expansions for each state and year [1]. We obtain state-by-year unemployment rates from the Bureau of Labor Statistics [54], and state-by-year indicators of ABAWD work requirement waivers from the US Department of Agriculture Food and Nutrition Service, organized by the Center on Budget and Policy Priorities [50].

The CPS ASEC study population includes individuals with family incomes under 138 percent of the federal poverty level. We calculate income as a percentage of the federal poverty level using ASEC data on income and family size, and published federal Health and Human Services annual poverty guidelines. Study participants can be any age, but they must reside with a household member between 18 and 64 years old, the age group targeted by the Medicaid expansion. We use the full population available in the SNAP QC, and all SNAP participants in the December CPS. The Public Policy Institute of California Institutional Review Board conducted an exempt human subjects review, and approved this study in writing.

### Estimation

Not all states participated in the ACA Medicaid expansion, and there was inconsistent timing among those that did [1]. This state-time variation in implementation provides a natural experiment for investigating the relationship between the two programs. We use a difference-in-differences strategy to estimate the effect of the Medicaid expansion on SNAP receipt using the CPS ASEC (Eq 1). The outcome *Υ* represents binary SNAP receipt (yes/no), and *Postexp* is an indicator of the post-expansion period for Medicaid expansion states; this variable changes from 0 to 1 in the calendar year containing a state’s expansion. χ contains individual covariates: binary sex, continuous age and its square, binary marital status, indicators for race and Latino ethnicity, indicators for educational attainment of the household head, continuous family size, continuous number of minor children in the family, continuous income and its square. Indicators of characteristics such as race, ethnicity, and education level control for systematic differences in safety net program access attributable to language, information, discrimination, and other factors we are unable to capture explicitly in this study. All of the individual-level characteristics are from the CPS ASEC. *ω* contains state-year unemployment rates and indicators of ABAWD waivers. Full sets of state and year fixed effects are indicated by *γ* and *δ*, and *η* and *ε* represent state and individual disturbances. The subscripts *i*, *s*, and *t* reference individuals, states, and years, respectively.


$$\Upsilon_{ist}=\alpha_{0}+\beta_{1}Pos{texp}_{st}+\beta_{2}\chi_{it}^{\prime }+\beta_{3}\omega_{st}+\gamma_{s}+\delta_{t}+\eta_{st}+\varepsilon_{ist}$$ (1)

We estimate linear probability models, and coefficients are interpretable as percentage point changes in likelihood of receiving SNAP. In Eq 1, our parameter of interest is *β*_*1*_, the effect of the Medicaid expansion on SNAP participation. The models are estimated for the full study sample and for subgroups defined by income and the presence of children in the household. SNAP has additional work requirements for ABAWDs, meaning that there is an important hurdle that many non-parents must clear before gaining access, although not all non-parents are ABAWDs. For brevity, we refer to study members living in households with children as individuals “with children,” and to their counterparts in households without children as “without children.”

Although all of the individuals targeted by the Medicaid expansion are low-income, we stratify analyses by income to investigate potential heterogeneity of effects for different groups. We were interested in investigating this based on the past finding that, even within a group eligible for SNAP, the lowest-income individuals were most familiar with the program [30]. We use a cut point of 75 percent of FPL. This is close to the mean and the median income of the sample, making it a natural choice for defining income subgroups. Those at 75 percent of FPL have extremely low incomes and high need for nutritional assistance; in 2019, a family of four at this income level would earn less than $20,000.

Analyses of SNAP benefit amounts and household characteristics use the SNAP QC data and follow Eq 2. Here, study units are households, weighted to be representative and then averaged by state-year, rather than individuals. The outcomes, represented by *W*, are the monthly benefit amount in continuous dollars, the continuous number of SNAP households with $0 income, or the continuous number of households with an ABAWD member. We control for aggregate covariates that vary at the state-year level in *υ*: the maximum SNAP benefit amount, unemployment rates, and presence of ABAWD waivers. State and year fixed effects are controlled in *ρ* and *z*, and *ξ* represents state-year disturbances. The SNAP QC supplemental models are estimated as ordinary least-squares, and we interpret the coefficient estimate for the Medicaid expansion period, *ϕ*_*1*_. In all CPS ASEC and SNAP QC models, standard errors are robust to heteroskedasticity and clustered by state [55]. We use Stata MP versions 14 and 16.


$$W_{st}=c_{0}+\phi_{1}Pos{texp}_{st}+\phi_{2}{}_{st}+\rho_{s}+z_{t}+\xi_{st}$$ (2)

### Parallel trends assumption

We analyze pre-expansion trends to determine whether the parallel trends assumption underlying a causal interpretation of our difference-in-differences model is plausible. An event study version of difference-in-differences accounts for the fact that states expanded Medicaid in varying years. Eq 3 shows how SNAP participation is modeled using *Exp*, an indicator of being an expansion state and *Relyr*, the year relative to expansion year (2014 for non-expansion states). The terms X, w, g, d, n, and e are analogues of χ, ω, γ, δ, η, and ε from Eq 1.


$$\begin{array}{c} Y_{ist}=\sum_{j}\;a_{j}Exp_{s}Relyr_{sj}+b_{2}X_{it}^{\prime }+b_{3}w_{st}+g_{s}+d_{t}+n_{st}+e_{ist}\\ with\;j\in \{-3,-2,-1,0,1,2+\} \end{array}$$ (3)

**Fig 1:**
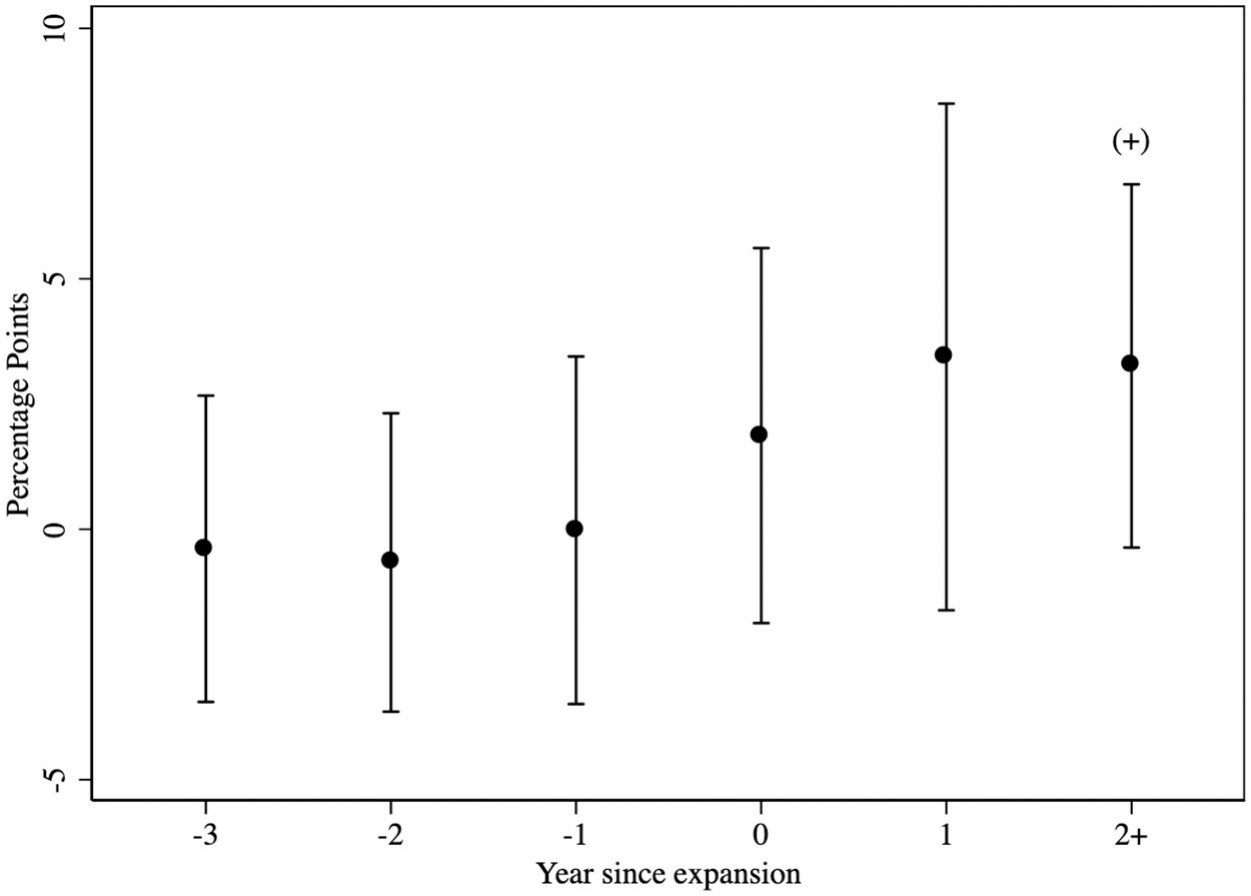
An event study test of the parallel trends assumption. +p<0.10 Differences in SNAP participation shown for Medicaid expansion status versus non-expansion states, relative to expansion year. Ninety-five-percent confidence intervals shown. Data source is the Current Population Survey ASEC, 2011–2020 (reporting periods 2010–2019).

The *a*_*j*_ terms illustrate how SNAP participation changed relative to the timing of Medicaid expansion. For parallel trends to be plausible, *a*_*-3*_, *a*_*-2*_, *a*_*-1*_ should be close to zero since they capture the differences in SNAP between expansion and non-expansion states in the years just before expansion. The coefficients *a*_*0*_, *a*_*1*_, *a*_*2+*_ represent differences in the year of expansion, the first year after, and two or more years after; being greater than zero would lend support to our hypothesis. The *a*_*j*_ terms follow the desired patterns, and are consistent with a causal interpretation of the main model (Fig 1).

### Placebo tests

We explore whether our findings reflect a phenomenon other than Medicaid expansion’s effect on SNAP with placebo tests. If something were driving changes to SNAP that happened to coincide with the timing of Medicaid expansion, the unrelated driver likely affected individuals beyond those who signed up for Medicaid. In the first placebo test, we conduct the difference-in-differences model for low-income individuals in households with only senior citizen members. No household members are eligible for Medicaid expansion, but they could be affected by an unrelated driver, and are not barred from taking up SNAP. We conduct a similar test using low-income individuals who have employer-supplied insurance. Some may switch from private coverage to Medicaid mid-year or enroll in Medicaid and employer coverage at the same time; we include these individuals in our analysis. However, the majority of low-income individuals with employer coverage are unlikely to be connected to SNAP through Medicaid expansion since they have private coverage. One weakness is that, by definition, the group analyzed is employed, and they have higher income on average than the main study group; consequently, they may not be as likely to seek out SNAP. Positive SNAP takeup findings in either test would challenge our interpretation of the main models. Null results in these placebo tests would help support our findings.

### Study limitations

Our study contributes new evidence on an important policy topic, but it has limitations. Analyses are at the state-year level and do not capture what may be important administrative differences at the local level or seasonal changes in enrollment and recertification in Medicaid and SNAP. Our investigations of mechanisms focus on certain official policies that could facilitate a spillover effect on SNAP participation. They do not cover important unofficial policies such as attitudes, biases, or intentional delays by front-line workers, sometimes called “street-level bureaucracy,” that can strongly encourage or discourage participation [56]. Although we analyze certain subgroups, our findings are still average effects of expansion on low-income adults and not the reflection of any particular individual experiences. In particular, we note that our study defines expansion in a binary way, averaging states with large parental expansions with ones with small or no changes for parents despite the Medicaid expansion. This limitation biases our findings towards the null, so it should not lead to an overstatement of findings. Finally, our study is not designed to follow individuals over time to assess fluctuating program eligibility, churning enrollment, or other changes to participation in Medicaid or SNAP. The limitations of this study point to areas of future research, which we discuss later.

## Results

**Fig 2:**
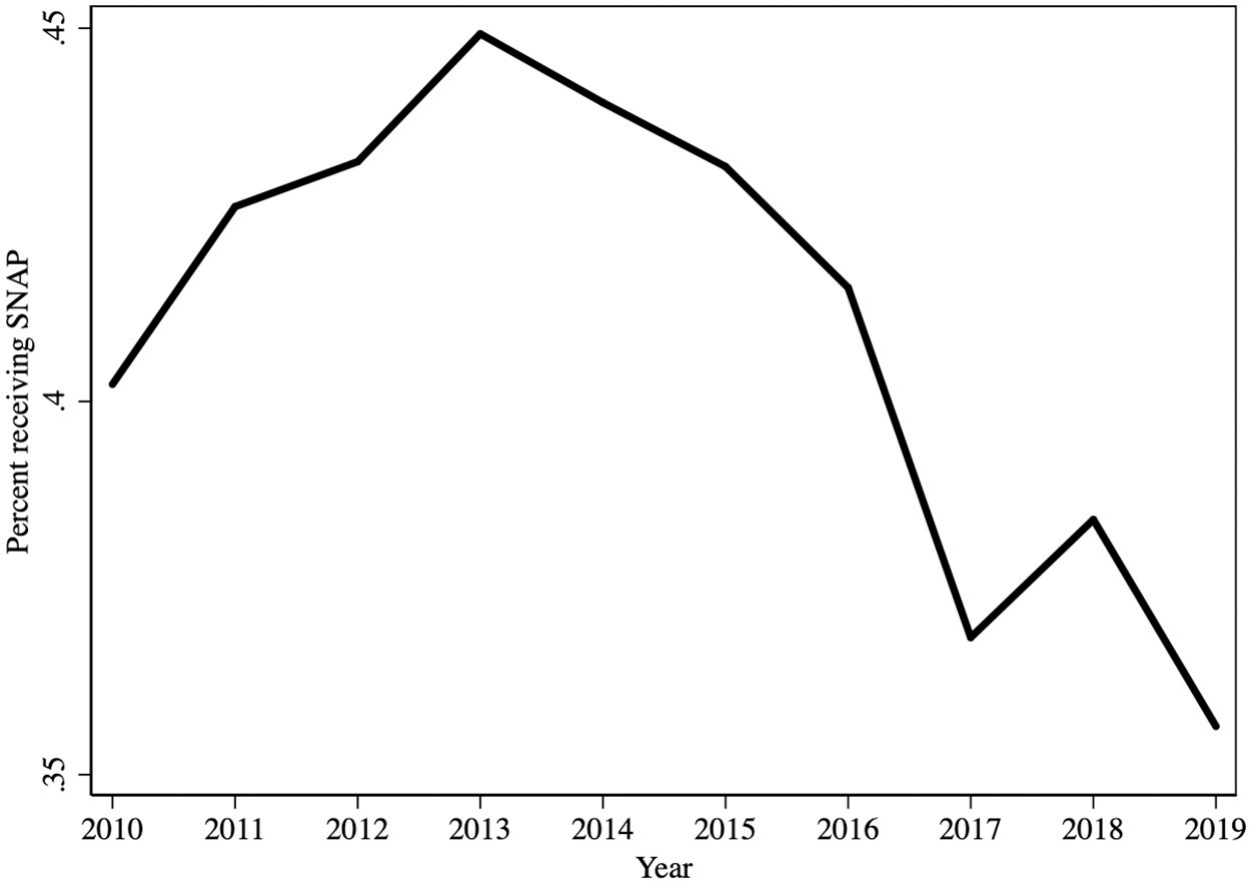
National trend in SNAP participation. Data source is the Current Population Survey ASEC, 2011–2020 (reporting periods 2010–2019). All 50 states and DC are included.

Fig 2 shows growing national participation in SNAP between 2010 and 2013 among the study population, followed by a decrease that reflects recovery from the recession [57]. Since the first Medicaid expansions began in 2014, any effect that the reform may have produced on SNAP would be in the context of the national decline in participation.

### Verification of changes to Medicaid

Though many studies have documented that Medicaid expansion successfully increased Medicaid coverage for low-income adults [58], we verify that this change is detectible in our data and study population as a preliminary step. We estimate the effect of the expansion on Medicaid coverage using the difference-in-differences approach described in Eq 1. The results are highly significant increases in Medicaid coverage (S1 Appendix), confirming it is reasonable to investigate downstream effects such as SNAP participation.

### Main findings on SNAP participation

The Medicaid expansion produced a highly significant 2.9 percentage point increase (p = 0.002) in SNAP participation. This translates to about a 7 percent increase (relative to the mean) during a period of declining national SNAP participation (Table 1). Effects are about 2.4 percentage points for those with children, and 3.5 percentage points for those without children. These estimates are all significantly different from zero, but the 13 percent increase for those in all-adult study households is especially meaningful, since their average SNAP participation rate is much lower than for those with children (27.7 percent versus 47.8 percent).

**Table 1:** ACA Medicaid expansion effects on SNAP receipt by household composition.

	In all HHs	In HHs with	In HHs with
		Children	No Children
Effect of Medicaid Expansion	0.029**	0.024*	0.035**
Robust standard error	(0.009)	(0.011)	(0.013)
P-value	0.002	0.030	0.007
N	413893	306533	107360
Mean of dependent variable	0.416	0.478	0.277

*p<0.05

**p<0.01

Data source is the Current Population Survey ASEC, 2011–2020 (reporting periods 2010–2019). All 50 states and DC are included. Difference-in-differences regressions are linear probability models using survey weights. The unit of analysis is the individual, and stratification is by presence of children in the household. Standard errors in parentheses are robust to heteroskedasticity and clustered by state.

The very-low-income individuals without children experience a large (5.4 percentage point) increase in SNAP participation, translating to more than a 19 percent increase (Table 2). The first column of Table 2 shows the overall results by income. Those between 75 and 138 percent of FPL have a 2.4 percentage point SNAP increase due to Medicaid expansion, while those under 75 percent of FPL have a 3.3 percentage point increase. Individuals with children have the same effect size of 2.4 percentage points regardless of how low income their households are (Table 2 center column), although precision declines to marginal significance (p<0.10) for these estimates. Among those with no children, all of the effect is concentrated in the lowest income group (Table 2, third column). Although the entire study population is low-income, the gradations of income mattered, and the poorest adults without children experience the largest and most meaningful SNAP increases due to the Medicaid expansions.

**Table 2:** ACA Medicaid expansion effects on SNAP receipt by household income and composition.

**75–138% FPL**			
	**In all HHs**	**HHs w/ Children**	**HHs w/o Children**
Effect of Medicaid Expansion	0.024*	0.024+	0.022
Robust standard error	(0.012)	(0.013)	(0.016)
P-value	0.042	0.069	0.183
N	206692	149764	56928
Mean of dependent variable	0.355	0.394	0.275
**<75% FPL**			
	**In all HHs**	**HHs w/ Children**	**HHs w/o Children**
Effect of Medicaid Expansion	0.033**	0.024+	0.050**
Robust standard error	(0.010)	(0.012)	(0.015)
P-value	0.002	0.051	0.002
N	207201	156769	50432
Mean of dependent variable	0.476	0.557	0.279

^+^ p<0.10

*p<0.05

**p< 0.01

Data source is the Current Population Survey ASEC, 2011–2020 (reporting periods 2010–2019). All 50 states and DC are included. Difference-in-differences regressions are linear probability models using survey weights. The unit of analysis is the individual, and stratification is by income and presence of children in the household. Standard errors in parentheses are robust to heteroskedasticity and clustered by state.

### Changes to benefit amounts, participating households

SNAP benefit amounts, which average around $258 per month in the study period, do not change as a result of Medicaid expansion (Table 3). However, there is a small increase in the number of households with $0 income. Together with the main results, we interpret these findings to mean that the Medicaid expansion may have helped SNAP reach the most destitute households with deep need for food assistance.

**Table 3:** ACA Medicaid expansion effects on SNAP benefit amount and characteristics of participating households.

	Benefit amount	SNAP HHs w/	SNAP HHs w/
	per SNAP HH	$0 Income	ABAWD Member
Effect of Medicaid Expansion	-0.150	15846.073*	12207.328
Robust standard error	(2.597)	(6694.702)	(7640.911)
P-value	0.954	0.022	0.116
N	510	510	510
Mean of dependent variable	$258.25	83182.904	40054.753

*p< 0.05

Data source is the SNAP Quality Control Data Fiscal Years 2010–2019. All 50 states and DC are included. Regressions are ordinary least-squares. The unit of analysis is SNAP households at the state-year level. The average SNAP household has two persons, and the mean benefit amount translates to about $130 per participant. The models of characteristics of participating households estimate the average number of households with either $0 income or an ABAWD member. Standard errors in parentheses are robust to heteroskedasticity and clustered by state.

Because individuals without children experience the largest increases in SNAP in both absolute and relative measures, we investigate whether Medicaid expansion increased the number of SNAP households participating that have ABAWD members. If this were the case, the Medicaid expansion would be a connection to SNAP for vulnerable individuals who have limited access to the safety net. However, analysis of participating households shows no change to the number with an ABAWD member as a result of Medicaid expansion (Table 3). We showed earlier that households without children have increased SNAP participation following expansion; this is consistent with Burney et al.’s findings. However, we find no evidence that ABAWDs’ access specifically improves. The ACA Medicaid expansion’s lack of categorical requirement means that the policy has the potential to be a point of entry to the safety net for ABAWDs. Either this does not occur, or alternately, Medicaid enrollment fails to produce engagement in SNAP. Medicaid enrollment information is not in the SNAP QC, and ABAWDs are difficult to identify accurately in large survey data such as the CPS because to do so would require information about care responsibilities and disability. Answering the question of ABAWD engagement in Medicaid is beyond the capability of our study, but it is an important one for future research to take up with appropriate data.

### Sensitivity tests of main findings

An alternate approach using low education rather than income to define the study population also finds that Medicaid expansion led to SNAP participation (S3 Appendix). The main models analyze individuals with incomes under 138 percent of federal poverty, but income is potentially endogenous to new Medicaid availability since families can adjust their income (e.g., by working fewer hours) to meet the program’s income requirements. If this kind of behavior were common in states that expanded Medicaid, our study’s key estimates would be biased upward. In this sensitivity test, the analytic sample includes individuals in families headed by an adult with a high school education or less. These are individuals with low socioeconomic status not defined by income. The effects of Medicaid expansion on SNAP for this group are positive and highly significant, though smaller magnitude (2.3 percentage point increase, p<0.001) compared to the main findings.

### Placebo tests

Null estimates in placebo tests of two low-income populations eligible for SNAP that were unlikely to be affected by the Medicaid expansion are consistent with the main findings. Low-income elderly individuals in entirely senior citizen households do not have their SNAP participation affected by the Medicaid expansion (S4 Appendix). The placebo test is the same difference-in-differences model for individuals under 138 percent of FPL in households entirely composed of senior citizens aged 65 and older, and it produces a null estimate. This is not simply due to a loss of power. The precision of the zero is comparable to some of the previously reported subgroup findings (se = 0.013) though the all-senior population is much smaller than the main study group. (Recall that the main study group includes seniors if they had an expansion-eligible household member.) Low-income individuals with employer-supplied insurance are less likely to be affected by the Medicaid expansion. Even including likely insurance switchers who report both Medicaid and private coverage in the same calendar year (and therefore may have experienced spillover into SNAP), the model produces a null estimate with higher precision (se = 0.011) than the all-senior model (S4 Appendix).

### Timing of SNAP participation relative to Medicaid expansion

**Fig 3:**
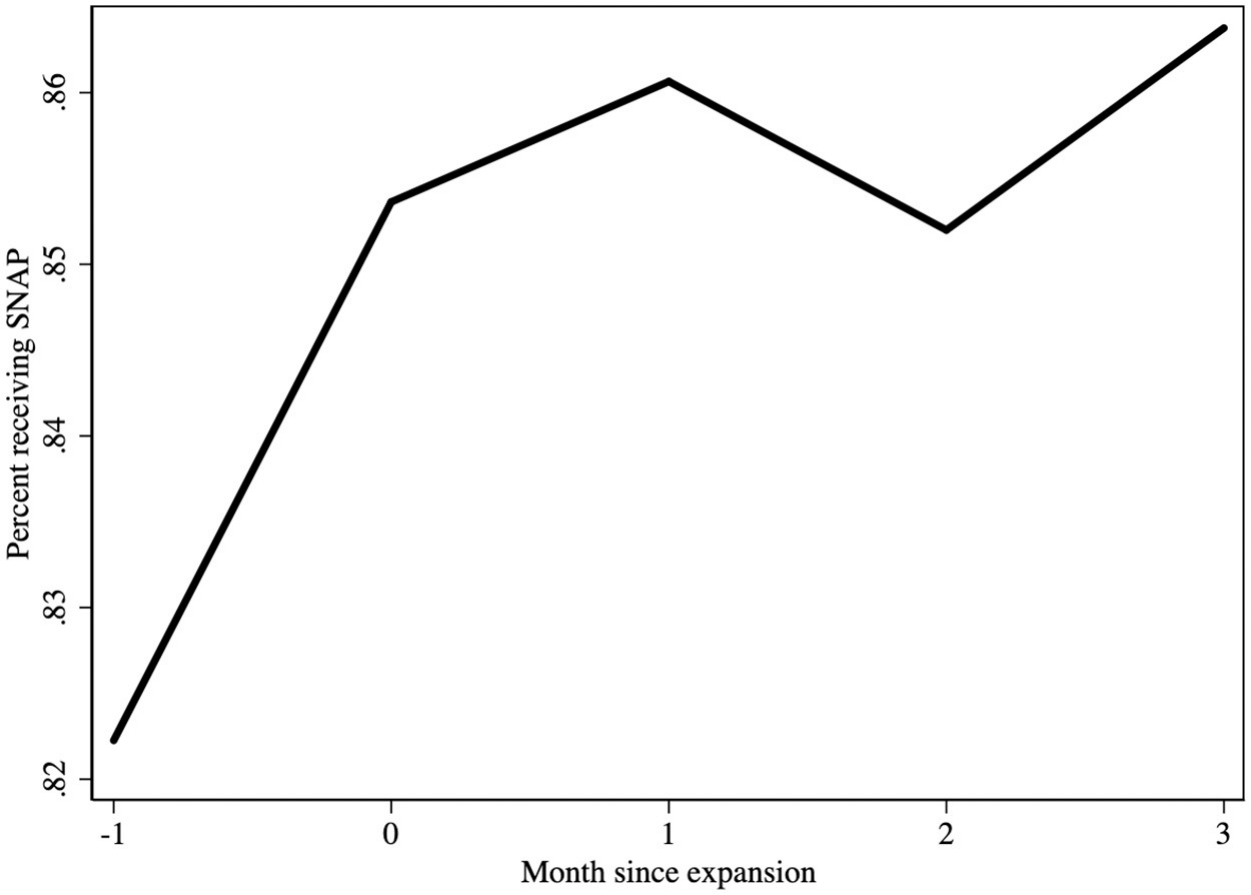
SNAP receipt patterns relative to Medicaid expansion month. Data source is the Current Population Survey December Supplement, 2014–2016. Includes only individuals reporting SNAP receipt in state-years with non-January Medicaid expansions (IN, MI, NH, LA, AK).

Both joint processing and Express Lane strategies allow participants in programs including SNAP to have automatic or streamlined access to Medicaid, which suggest that it is possible for spillover to operate in the other direction. We investigate whether this is the driver of our findings. The December CPS provides evidence that in cases of mid-year Medicaid expansions, new Medicaid eligibility preceded higher SNAP enrollment, supporting our interpretation that Medicaid led to SNAP rather than the other way around. Indiana, Michigan, New Hampshire, Louisiana, and Alaska implemented Medicaid expansion in a month other than January. In expansion years for these states only, we plotted monthly SNAP receipt among those who reported receiving any SNAP benefit that year (Fig 3). If receipt rose in response to forces unrelated to Medicaid expansion, there would be a chance that benefit receipt rates would be similar or higher pre-expansion compared to post-expansion. Fig 3 plots average SNAP participation in the set of months that are available in all of the states. The participation rates are much higher than in the main analysis since the sample includes only SNAP participants. Monthly receipt rose by about 3 percentage points in the month Medicaid expanded, and remained at least that high afterwards. This pattern and the similarity of the magnitude increase to our main findings support our interpretation that SNAP participation increased due to Medicaid expansion.

### Investigation of possible mechanisms

We sought to investigate mechanisms for how the Medicaid expansion led to increased SNAP participation; joint processing of Medicaid and SNAP applications and ACA healthcare navigators arose as two candidates among a range of policy options. The federal government defines eligibility requirements for SNAP, but states vary in certain details of the program administration [32]. Some states jointly process SNAP and Medicaid applications, ensuring that individuals applying for one of the programs are considered for the other [32, 59]. Because joint processing affects the applications for both programs, it could be a pathway through which Medicaid expansion affected SNAP. (States report the optional policy decisions they implement in SNAP to the US Department of Agriculture’s Food and Nutrition Service, including joint processing; this information is available in public reports [48].) We investigate joint processing status using a triple-differences analysis. This produces similar SNAP increases in joint and non-joint-processing states for eligible individuals overall, and for those with children (S4 Appendix). For those without children, however, the effect of Medicaid expansion on SNAP is only present in states with joint processing, suggesting that jointly processing applications may be a driver of spillover for this group.

Another possible mechanism is that ACA healthcare navigators could have guided individuals to apply for the programs. The navigators are intended to help with selecting and enrolling in marketplace health plans. However, they can also help eligible individuals who might hesitate to engage with government programs enroll in Medicaid [37], reflecting “no wrong door” enrollment approaches. Many states received federal grants to implement navigator programs, typically through community-based organizations or technical assistance providers. For investigating the possible role of navigators, we used per-capita navigator grant amounts to operationalize the availability of assistance; the grant amounts used are posted publicly by the federal government [60]. We find no evidence that navigators were associated with a change in SNAP participation (S4 Appendix). We also find no combined effect of the Medicaid expansion and navigators on SNAP (S4 Appendix).

## Discussion

SNAP, by addressing nutritional needs, can improve social determinants of health. These are life conditions outside of the health care system—such as insufficient food—that affect health. Low-income individuals are more likely to have poorer diets [61], which is associated with poorer health [62]. Low-income patients are also less likely to improve their diets in response to physician recommendations than those with higher incomes [63], possibly because they lack the resources to do so. We find support for our first hypothesis that Medicaid expansion leads to greater SNAP participation. The 2.9 percentage point overall effect we estimate translates to a 7 percent increase in SNAP. This effect size is in the same range as comparable findings by other researchers even though the studies use a variety of settings, data sources, and methods. Our estimate is smaller than national findings by Lanese et al. (10 percent) or Burney et al. (17 percent), and larger than findings in the Oregon Health Insurance Experiment (4 percent), or Schmidt et al.’s study of border counties (4 percent). Our estimate is substantially larger than Agirdas’ (0.49 percent), though the latter compares differentially treated counties, not treated versus control counties. Since overall SNAP participation declines in the post-ACA period (Fig 2), the increase is likely to be a combination of new enrollments and continued participation among individuals who otherwise would have been disenrolled. We do not know for certain which effect dominates since we do not follow individuals over time.

Though we had hypothesized that presence of children would predict a larger effect, we found the opposite. In households with children, the average rates of participation in Medicaid and SNAP are higher in the study period than households without children. These higher baselines are likely to be some of the reason spillover effects were smaller in households with children. Still, we find a 2.4 percentage point increase for these individuals, implying that some low-income children—who are not the focus of the Medicaid expansion—benefit indirectly through more access to SNAP. Even if these effects are principally continued enrollment, the consequences are meaningful. Evidence shows that losing SNAP is associated with an array of negative economic and health consequences for children and families [64].

We find strong support for our third hypothesis, which stated that spillover may be concentrated among the poorest eligible households. We find substantially larger effects in very-low-income households under 75 percent of FPL without children: about a 5.0 percentage point (19 percent) increase, though this partially reflects the fact that SNAP and Medicaid outside of the ACA expansion are more available for parents than for non-parents, resulting in lower baseline participation rates for the latter group. The increase in SNAP in very-low-income households is likely to provide substantial improvements in access to food. SNAP is a graduated program, with larger benefit amounts allocated to households with more members or greater need. The lowest income individuals therefore can gain the most nutritional assistance, in absolute and relative terms, when they participate.

We find no evidence to support our final hypothesis that ABAWDs would experience a large spillover effect. Although both our and the Burney et al. findings for households without children suggest that ABAWDs could be connected to SNAP following Medicaid expansion, we find this is not the case. We find no effect on participation by households with an ABAWD member. ABAWDs have largely been excluded from social safety net programs, which tend to prioritize working adults and families with children. Whether Medicaid does not succeed in enrolling this underserved group, or it fails to produce further engagement with the safety net beyond public insurance, is an important question for future research to answer.

Our study finds that getting connected to Medicaid increases SNAP participation, a program that addresses hunger, an important social determinant of poor health associated with being low-income. States that have not expanded Medicaid under the ACA can use these findings to reevaluate cost-benefit calculations. The federal government currently pays for 90 percent of the cost of covering the ACA Medicaid expansion population [1], and SNAP is a federal program. The net cost to states for implementing Medicaid expansion and experiencing SNAP spillover participation is relatively low compared to the amount of support their residents receive. In the long run, these investments would help keep their populations healthier, further magnifying the benefits of expanding Medicaid.

Determining how Medicaid expansion leads to improved SNAP access can inform policy investments in all states. Lanese et al. estimated an average effect of Medicaid expansion combined with at least one of several policies to streamline enrollment. They found large effects of the combined changes, but they did not investigate the role of any specific outreach policy or effects for different eligible groups. We extend this knowledge in our analyses of joint processing and navigators as possible linkages between Medicaid and SNAP for several household types. We find joint processing is especially helpful for facilitating SNAP enrollment in households without children. This finding has implications for Medicaid and SNAP, and the implications can be extrapolated to other programs. Joint processing with Temporary Assistance for Needy Families and combined applications for Supplemental Security Income are both official SNAP options that states can choose that would reduce transaction costs for individuals eligible for multiple programs [48]. Opting into joint processing and other coordination measures such as Express Lane eligibility across multiple safety net programs would benefit all eligible individuals by reducing administrative burden, and the approach appears to be especially valuable for reaching individuals in households without children. We found no effect of navigators, alone or in combination with Medicaid expansion, on SNAP participation. Our findings do not rule out the importance of navigators in their official role assisting individuals to get insurance coverage. They do suggest, however, that navigators are not a likely link between Medicaid expansion and increased SNAP participation.

Our study arrives at an important time for assessing the Medicaid expansion policy. After being rolled out in a period of improving economic conditions, the coronavirus pandemic and associated economic difficulties are the first true test of Medicaid expansion as a countercyclical safety net program. Its positive effects on SNAP participation are especially beneficial during a time of illness and economic uncertainty.

This study should not be the last word on connections between Medicaid and SNAP. Future research should address a wider range of possible mechanisms that could facilitate positive spillovers; local efforts may be especially fruitful to study. In many states, counties and cities play a large role in verifying eligibility and enrolling individuals in programs. Studying their work can provide information about which strategies are the easiest to adopt, which show high promise for connecting eligible individuals to multiple services, and how to balance these two objectives. Additionally, community-based organizations that help their clients enroll in programs may have tailored, culturally and linguistically appropriate approaches that merit study and consideration for scale-up. We did not take on the subject of new technology in this project, but efforts like Code for America’s Integrated Benefits Application are able to reach large numbers of eligible individuals, which can be especially useful if street-level bureaucrats do not conduct outreach or if they discourage applications [65]. Understanding the roles of these and other overlapping strategies can help policy-makers and communities direct investments to productive measures, and connect eligible individuals to programs they need.

## Conclusion

We find that the ACA Medicaid expansion connects vulnerable individuals to SNAP, the primary nutritional safety net program in the country. This main finding is consistent with the Oregon Health Insurance Experiment, as well as the handful of ACA Medicaid expansion studies in this area of research. The spillover affects children, who are not the target of the expansion, and produces large effects for very-low-income adults, many of whom were not connected to SNAP despite their limited resources. Joint processing of Medicaid and SNAP appears to facilitate the spillover effect, suggesting that reducing administrative burden would be helpful for improving access to multiple safety net programs. Although SNAP is a federal program and Medicaid is a state-federal program, states can streamline applications, recertifications, and other hurdles to accessing and staying enrolled in these programs. We find no spillover effect for ABAWDs, however, who are a vulnerable group of adults that need additional outreach and support to access programs for which they may be eligible. Our findings contribute to a body of evidence that the Medicaid expansion does more than improve access to health care; it connects eligible low-income individuals to multiple supports. Enrolling in SNAP increases access to food, an important social determinant of health, and an investment in population health for states.

## Supporting information

S1 Appendix Medicaid outcome. (DOCX)

S2 Appendix Full regression output for tables. (DOCX)

S3 Appendix Alternate populations. (DOCX)

S4 Appendix Possible mechanisms. (DOCX)

S5 Appendix Underlying numbers Figs 1–3. (XLSX)
