# Supplementary material for: The Affordable Care Act Medicaid expansion: A difference-in-differences study of spillover participation in SNAP
Source: PLoS One. 2022 May 4;17(5):e0267244. doi: 10.1371/journal.pone.0267244 (PMC9067645; doi:10.1371/journal.pone.0267244)
Supplement: S1 Appendix — (DOCX) [file pone.0267244.s001.docx]

**S1 Appendix: Medicaid Outcome**

| **ACA Medicaid Expansion Effects on Medicaid Coverage by Household Composition** | | | |
| --- | --- | --- | --- |
|  |  |  |  |
|  | **In all HHs** | **In HHs with** | **In HHs with** |
|  |  | **Children** | **No Children** |
| Effect of Medicaid Expansion | 0.058*** | 0.043*** | 0.089*** |
| Robust standard error | (0.009) | (0.011) | (0.012) |
| P-value | <0.001 | <0.001 | <0.001 |
| N | 413728 | 306368 | 107360 |
| Mean of dependent variable | 0.471 | 0.553 | 0.287 |

***p< 0.001 Data source is the Current Population Survey ASEC, 2011-2020 (reporting periods 2010-2019). All 50 states and DC are included. Infants not alive in previous year excluded. Difference-in-differences regressions are linear probability models using survey weights. The unit of analysis is the individual, and stratification is by presence of children in the household. Standard errors in parentheses are robust to heteroskedasticity and clustered by state.

**Difference-in-differences results for full population: full regression results**

Linear regression Number of obs = 413,728

F(24, 50) = .

Prob > F = .

R-squared = 0.1785

Root MSE = .45245

(Std. Err. adjusted for 51 clusters in statefip)

------------------------------------------------------------------------------

| Robust

medicaid | Coef. Std. Err. t P>|t| [95% Conf. Interval]

-------------+----------------------------------------------------------------

medicaid

expansion | .0579418 .0090278 6.42 0.000 .039809 .0760747

|

year |

2011 | .0156235 .0055726 2.80 0.007 .0044307 .0268164

2012 | .0150419 .0073793 2.04 0.047 .0002202 .0298636

2013 | .0394479 .0093301 4.23 0.000 .0207078 .0581881

2014 | .0288162 .0122005 2.36 0.022 .0043108 .0533215

2015 | .023613 .0144431 1.63 0.108 -.0053969 .0526229

2016 | .0352707 .0175936 2.00 0.050 -.0000671 .0706086

2017 | .0211278 .0174875 1.21 0.233 -.0139968 .0562523

2018 | .0676353 .0225668 3.00 0.004 .0223085 .1129621

2019 | .064483 .0212814 3.03 0.004 .021738 .107228

|

statefip |

2 | -.0352234 .0046615 -7.56 0.000 -.0445863 -.0258606

4 | .0752531 .0056342 13.36 0.000 .0639364 .0865698

5 | .0262934 .0053248 4.94 0.000 .0155983 .0369885

6 | .0930629 .006408 14.52 0.000 .0801921 .1059337

8 | -.0102385 .0070545 -1.45 0.153 -.0244079 .0039308

9 | .0979101 .0049226 19.89 0.000 .0880229 .1077974

10 | .07286 .005897 12.36 0.000 .0610154 .0847045

11 | .1698128 .0058727 28.92 0.000 .1580171 .1816085

12 | -.0003251 .0033909 -0.10 0.924 -.007136 .0064858

13 | -.0373174 .0012007 -31.08 0.000 -.039729 -.0349057

15 | -.0218513 .0099513 -2.20 0.033 -.0418391 -.0018636

16 | -.0286079 .0058474 -4.89 0.000 -.0403527 -.016863

17 | .0512595 .0048233 10.63 0.000 .0415717 .0609474

18 | .0421736 .0039082 10.79 0.000 .0343238 .0500235

19 | .026964 .0098111 2.75 0.008 .0072578 .0466701

20 | -.02543 .0074623 -3.41 0.001 -.0404184 -.0104416

21 | .0536306 .0047319 11.33 0.000 .0441262 .063135

22 | .0236737 .0036459 6.49 0.000 .0163506 .0309968

23 | .1827927 .003935 46.45 0.000 .1748889 .1906964

24 | -.0014233 .0064749 -0.22 0.827 -.0144286 .011582

25 | .1896494 .0064547 29.38 0.000 .1766848 .2026141

26 | .0979538 .005489 17.85 0.000 .0869289 .1089786

27 | .0507502 .0083475 6.08 0.000 .0339838 .0675166

28 | .0566499 .0024499 23.12 0.000 .0517291 .0615707

29 | -.0223916 .0028388 -7.89 0.000 -.0280935 -.0166897

30 | .0136176 .0062959 2.16 0.035 .000972 .0262632

31 | -.0491568 .0120996 -4.06 0.000 -.0734595 -.0248541

32 | -.0789874 .0074853 -10.55 0.000 -.094022 -.0639527

33 | -.0176363 .0098524 -1.79 0.080 -.0374255 .0021529

34 | .030036 .00523 5.74 0.000 .0195313 .0405408

35 | .0795109 .0059357 13.40 0.000 .0675887 .091433

36 | .1378119 .0051689 26.66 0.000 .1274299 .1481939

37 | .0177981 .0013228 13.45 0.000 .0151411 .0204551

38 | -.1226044 .0129369 -9.48 0.000 -.148589 -.0966199

39 | .0776751 .004658 16.68 0.000 .0683192 .0870309

40 | -.0041304 .0072475 -0.57 0.571 -.0186874 .0104266

41 | .0725876 .0046028 15.77 0.000 .0633426 .0818326

42 | .0693713 .004016 17.27 0.000 .061305 .0774377

44 | .0940458 .0054161 17.36 0.000 .0831671 .1049244

45 | .0126586 .0006481 19.53 0.000 .0113567 .0139604

46 | -.0262802 .0105385 -2.49 0.016 -.0474473 -.0051131

47 | .0502363 .0014421 34.84 0.000 .0473398 .0531328

48 | -.0598746 .007489 -8.00 0.000 -.0749166 -.0448325

49 | -.1001283 .0091714 -10.92 0.000 -.1185495 -.0817071

50 | .1688458 .0099754 16.93 0.000 .1488096 .1888821

51 | -.0713806 .0067365 -10.60 0.000 -.0849113 -.0578499

53 | .0577577 .0045507 12.69 0.000 .0486173 .066898

54 | .1337194 .0050784 26.33 0.000 .1235192 .1439196

55 | .0989908 .0044461 22.26 0.000 .0900605 .1079211

56 | -.0398191 .0071318 -5.58 0.000 -.0541437 -.0254945

|

male | -.0311353 .0033805 -9.21 0.000 -.0379253 -.0243454

age | -.0162329 .0013378 -12.13 0.000 -.0189201 -.0135458

age2 | .0001531 .0000161 9.54 0.000 .0001209 .0001854

race_w | -.010321 .0054351 -1.90 0.063 -.0212376 .0005956

race_b | .062195 .0075339 8.26 0.000 .0470628 .0773272

latino | -.0051783 .0102833 -0.50 0.617 -.0258329 .0154763

married | -.0149908 .0097412 -1.54 0.130 -.0345566 .004575

famsize | -.0256918 .0020043 -12.82 0.000 -.0297174 -.0216661

fpl_pct | .0031335 .0001374 22.81 0.000 .0028575 .0034095

fpl_pct2 | -.0000278 9.01e-07 -30.90 0.000 -.0000296 -.000026

hhkids | .0584537 .0019659 29.73 0.000 .054505 .0624023

educ_1 | .1815418 .0084579 21.46 0.000 .1645536 .19853

educ_2 | .1448381 .0061801 23.44 0.000 .132425 .1572511

educ_3 | .1040978 .0057422 18.13 0.000 .0925642 .1156314

unempl | -.0061985 .0035186 -1.76 0.084 -.0132657 .0008688

_cons | .5540051 .0391555 14.15 0.000 .4753591 .6326512

------------------------------------------------------------------------------

**Difference-in-differences results for those in households with children: full regression results**

Linear regression Number of obs = 306,368

F(24, 50) = .

Prob > F = .

R-squared = 0.1691

Root MSE = .45325

(Std. Err. adjusted for 51 clusters in statefip)

------------------------------------------------------------------------------

| Robust

medicaid | Coef. Std. Err. t P>|t| [95% Conf. Interval]

-------------+----------------------------------------------------------------

medicaid

expansion | .0429966 .0106873 4.02 0.000 .0215305 .0644626

|

year |

2011 | .0174454 .0055139 3.16 0.003 .0063704 .0285205

2012 | .0186025 .008878 2.10 0.041 .0007706 .0364344

2013 | .054728 .0109811 4.98 0.000 .0326717 .0767842

2014 | .0405253 .0141551 2.86 0.006 .012094 .0689566

2015 | .0362066 .0181225 2.00 0.051 -.0001936 .0726068

2016 | .0460066 .0201924 2.28 0.027 .005449 .0865643

2017 | .0272112 .0190158 1.43 0.159 -.0109831 .0654055

2018 | .0892469 .0261174 3.42 0.001 .0367886 .1417053

2019 | .0883197 .0246229 3.59 0.001 .0388632 .1377762

|

statefip |

2 | -.0551257 .0049148 -11.22 0.000 -.0649973 -.0452541

4 | .0886817 .0074563 11.89 0.000 .0737052 .1036582

5 | .046358 .0064501 7.19 0.000 .0334026 .0593133

6 | .1083538 .0079945 13.55 0.000 .0922963 .1244113

8 | .0010743 .0097806 0.11 0.913 -.0185706 .0207192

9 | .0941176 .0064367 14.62 0.000 .0811891 .1070462

10 | .0736676 .0073425 10.03 0.000 .0589198 .0884155

11 | .2151411 .0072625 29.62 0.000 .200554 .2297283

12 | .008273 .0035096 2.36 0.022 .0012239 .0153222

13 | -.0297955 .0015856 -18.79 0.000 -.0329803 -.0266107

15 | -.012004 .011177 -1.07 0.288 -.0344536 .0104455

16 | -.0168741 .0077969 -2.16 0.035 -.0325347 -.0012135

17 | .0894527 .0059175 15.12 0.000 .0775671 .1013383

18 | .0468756 .0043429 10.79 0.000 .0381526 .0555987

19 | .0144092 .0125088 1.15 0.255 -.0107156 .0395339

20 | -.0305874 .0094589 -3.23 0.002 -.0495861 -.0115887

21 | .0540522 .0052244 10.35 0.000 .0435587 .0645458

22 | .0267312 .0042577 6.28 0.000 .0181793 .0352831

23 | .2087037 .0053891 38.73 0.000 .1978795 .219528

24 | .009975 .0079012 1.26 0.213 -.0058951 .025845

25 | .1774318 .0084032 21.11 0.000 .1605534 .1943101

26 | .1053063 .006144 17.14 0.000 .0929657 .1176468

27 | .0366303 .0107186 3.42 0.001 .0151014 .0581593

28 | .0577016 .0033303 17.33 0.000 .0510125 .0643906

29 | -.0401703 .0038074 -10.55 0.000 -.0478176 -.032523

30 | .025045 .0079494 3.15 0.003 .0090783 .0410118

31 | -.0406757 .0151714 -2.68 0.010 -.0711484 -.0102029

32 | -.0833996 .0087742 -9.51 0.000 -.1010231 -.0657761

33 | -.0155464 .0121414 -1.28 0.206 -.0399331 .0088403

34 | .0292536 .0067561 4.33 0.000 .0156837 .0428236

35 | .0871762 .0074337 11.73 0.000 .0722452 .1021073

36 | .1407604 .0065966 21.34 0.000 .1275108 .1540101

37 | .0319902 .0015683 20.40 0.000 .0288402 .0351402

38 | -.1190073 .0160326 -7.42 0.000 -.1512098 -.0868049

39 | .1031201 .0055002 18.75 0.000 .0920726 .1141676

40 | .0057972 .008888 0.65 0.517 -.0120549 .0236493

41 | .0781585 .0056083 13.94 0.000 .0668938 .0894231

42 | .0598148 .005094 11.74 0.000 .0495831 .0700464

44 | .074852 .0065152 11.49 0.000 .0617658 .0879382

45 | .0146412 .0011206 13.07 0.000 .0123904 .016892

46 | -.0215516 .012764 -1.69 0.098 -.0471888 .0040856

47 | .0697143 .0019677 35.43 0.000 .0657621 .0736664

48 | -.0572114 .0092873 -6.16 0.000 -.0758654 -.0385574

49 | -.1151087 .011638 -9.89 0.000 -.1384843 -.091733

50 | .1513145 .012521 12.08 0.000 .1261653 .1764637

51 | -.0693251 .008169 -8.49 0.000 -.085733 -.0529172

53 | .0600014 .0054429 11.02 0.000 .0490689 .0709339

54 | .120906 .0057298 21.10 0.000 .1093974 .1324147

55 | .1107617 .0057134 19.39 0.000 .099286 .1222375

56 | -.0278734 .0091286 -3.05 0.004 -.0462088 -.009538

|

male | -.0302068 .0039136 -7.72 0.000 -.0380675 -.0223461

age | -.0168456 .0013743 -12.26 0.000 -.0196061 -.0140852

age2 | .0001461 .0000168 8.70 0.000 .0001124 .0001798

race_w | -.0123108 .0077174 -1.60 0.117 -.0278117 .0031901

race_b | .0537528 .0090056 5.97 0.000 .0356646 .071841

latino | -.0064608 .0106 -0.61 0.545 -.0277516 .0148299

married | -.0071944 .0125201 -0.57 0.568 -.0323418 .017953

famsize | -.0294034 .0016876 -17.42 0.000 -.032793 -.0260138

fpl_pct | .0022794 .0002033 11.21 0.000 .001871 .0026878

fpl_pct2 | -.0000238 1.41e-06 -16.85 0.000 -.0000267 -.000021

hhkids | .0461776 .0025833 17.88 0.000 .0409888 .0513663

educ_1 | .1577616 .0090903 17.36 0.000 .1395033 .1760199

educ_2 | .1339059 .0057409 23.33 0.000 .1223751 .1454368

educ_3 | .112643 .0066765 16.87 0.000 .0992328 .1260531

unempl | -.0062107 .0041601 -1.49 0.142 -.0145664 .002145

_cons | .6598452 .0375609 17.57 0.000 .5844019 .7352884

------------------------------------------------------------------------------

**Difference-in-differences results for those in households with NO children: full regression results**

Linear regression Number of obs = 107,360

F(23, 50) = .

Prob > F = .

R-squared = 0.1085

Root MSE = .4274

(Std. Err. adjusted for 51 clusters in statefip)

------------------------------------------------------------------------------

| Robust

medicaid | Coef. Std. Err. t P>|t| [95% Conf. Interval]

-------------+----------------------------------------------------------------

medicaid

expansion | .0886259 .0119777 7.40 0.000 .064568 .1126838

|

year |

2011 | .0077511 .0072806 1.06 0.292 -.0068725 .0223747

2012 | -.0007213 .0109281 -0.07 0.948 -.022671 .0212284

2013 | -.0046776 .0115699 -0.40 0.688 -.0279165 .0185613

2014 | -.002744 .0116702 -0.24 0.815 -.0261843 .0206962

2015 | -.0086185 .0177352 -0.49 0.629 -.0442408 .0270038

2016 | .0057112 .0189597 0.30 0.764 -.0323704 .0437928

2017 | .0043901 .0241012 0.18 0.856 -.0440187 .0527988

2018 | .0203078 .0256841 0.79 0.433 -.0312802 .0718957

2019 | .0088515 .0250328 0.35 0.725 -.0414284 .0591314

|

statefip |

2 | .0345224 .005865 5.89 0.000 .0227421 .0463027

4 | .0820132 .0066152 12.40 0.000 .0687262 .0953001

5 | -.001648 .0065094 -0.25 0.801 -.0147225 .0114264

6 | .1011135 .0080495 12.56 0.000 .0849456 .1172815

8 | .0174614 .0067643 2.58 0.013 .0038749 .0310479

9 | .1162284 .005779 20.11 0.000 .104621 .1278357

10 | .090549 .0065013 13.93 0.000 .0774908 .1036073

11 | .1595235 .0078802 20.24 0.000 .1436957 .1753513

12 | .0048957 .0037433 1.31 0.197 -.002623 .0124144

13 | -.0294392 .0015826 -18.60 0.000 -.032618 -.0262603

15 | -.0140844 .0125555 -1.12 0.267 -.0393029 .0111341

16 | -.0163589 .0057938 -2.82 0.007 -.027996 -.0047218

17 | -.0051354 .006614 -0.78 0.441 -.0184201 .0081493

18 | .0353016 .0056782 6.22 0.000 .0238966 .0467066

19 | .0865021 .0100365 8.62 0.000 .0663433 .1066609

20 | .0088685 .0076665 1.16 0.253 -.0065301 .024267

21 | .0529703 .0067538 7.84 0.000 .039405 .0665356

22 | .0307822 .0042445 7.25 0.000 .0222569 .0393075

23 | .1490033 .0053394 27.91 0.000 .1382789 .1597278

24 | -.0104516 .006516 -1.60 0.115 -.0235395 .0026363

25 | .2316226 .0073727 31.42 0.000 .2168141 .2464311

26 | .1015326 .0073694 13.78 0.000 .0867306 .1163345

27 | .1140987 .0090042 12.67 0.000 .0960133 .1321842

28 | .0654763 .0022886 28.61 0.000 .0608796 .0700731

29 | .0322083 .0038401 8.39 0.000 .0244952 .0399213

30 | .0200449 .0076143 2.63 0.011 .0047512 .0353385

31 | -.0296355 .0123514 -2.40 0.020 -.054444 -.004827

32 | -.0378217 .0096443 -3.92 0.000 -.0571929 -.0184505

33 | -.0233031 .0107764 -2.16 0.035 -.0449481 -.0016582

34 | .0476443 .0062999 7.56 0.000 .0349906 .060298

35 | .0838223 .0064023 13.09 0.000 .070963 .0966816

36 | .1575333 .0055845 28.21 0.000 .1463165 .16875

37 | .0106881 .0014761 7.24 0.000 .0077234 .0136529

38 | -.077411 .0131942 -5.87 0.000 -.1039123 -.0509097

39 | .0441225 .0061034 7.23 0.000 .0318634 .0563816

40 | -.0254773 .0074923 -3.40 0.001 -.040526 -.0104285

41 | .0972181 .0067062 14.50 0.000 .0837484 .1106879

42 | .1055284 .0050307 20.98 0.000 .095424 .1156328

44 | .1266016 .0076938 16.45 0.000 .1111481 .142055

45 | .018095 .0006698 27.02 0.000 .0167497 .0194402

46 | -.0198714 .0121233 -1.64 0.107 -.0442217 .0044789

47 | .0232559 .0018895 12.31 0.000 .0194608 .027051

48 | -.0335944 .0061256 -5.48 0.000 -.045898 -.0212908

49 | .0142538 .0081912 1.74 0.088 -.0021987 .0307062

50 | .2003121 .010974 18.25 0.000 .1782702 .2223539

51 | -.049317 .0062817 -7.85 0.000 -.0619342 -.0366998

53 | .0702385 .0066735 10.52 0.000 .0568343 .0836427

54 | .1404827 .0075361 18.64 0.000 .125346 .1556194

55 | .1030271 .0052915 19.47 0.000 .0923989 .1136553

56 | -.026912 .0077555 -3.47 0.001 -.0424893 -.0113346

|

male | -.0250741 .0030667 -8.18 0.000 -.0312338 -.0189145

age | .01917 .0016649 11.51 0.000 .0158259 .0225141

age2 | -.0001891 .0000193 -9.82 0.000 -.0002278 -.0001504

race_w | -.0289511 .0089199 -3.25 0.002 -.0468673 -.0110348

race_b | .0432242 .0094186 4.59 0.000 .0243064 .0621421

latino | -.0025855 .011265 -0.23 0.819 -.025212 .020041

married | -.0495243 .0051978 -9.53 0.000 -.0599644 -.0390843

famsize | -.0045059 .0039419 -1.14 0.258 -.0124234 .0034116

fpl_pct | .0045076 .0002637 17.09 0.000 .0039778 .0050373

fpl_pct2 | -.0000341 2.11e-06 -16.13 0.000 -.0000383 -.0000298

educ_1 | .1955973 .0094647 20.67 0.000 .176587 .2146077

educ_2 | .1191394 .0080535 14.79 0.000 .1029636 .1353153

educ_3 | .0744796 .005639 13.21 0.000 .0631533 .085806

unempl | -.0078954 .0034248 -2.31 0.025 -.0147743 -.0010166

_cons | -.3001587 .0507644 -5.91 0.000 -.402122 -.1981954

------------------------------------------------------------------------------
