# Supplementary material for: The Affordable Care Act Medicaid expansion: A difference-in-differences study of spillover participation in SNAP
Source: PLoS One. 2022 May 4;17(5):e0267244. doi: 10.1371/journal.pone.0267244 (PMC9067645; doi:10.1371/journal.pone.0267244)
Supplement: S2 Appendix — (DOCX) [file pone.0267244.s002.docx]

**S2 Full Regression Output for Tables**

**Table 1**

**Difference-in-differences full study population full regression results**

Linear regression Number of obs = 413,893

F(28, 50) = .

Prob > F = .

R-squared = 0.1250

Root MSE = .46113

(Std. Err. adjusted for 51 clusters in statefip)

------------------------------------------------------------------------------

| Robust

snap | Coef. Std. Err. t P>|t| [95% Conf. Interval]

-------------+----------------------------------------------------------------

medicaid expansion| .0289249 .0089788 3.22 0.002 .0108905 .0469593

|

year |

2011 | .0334086 .0090507 3.69 0.001 .0152297 .0515876

2012 | .0431908 .0102027 4.23 0.000 .022698 .0636835

2013 | .0654902 .0110757 5.91 0.000 .0432439 .0877365

2014 | .0485329 .0153031 3.17 0.003 .0177958 .07927

2015 | .0438437 .0210135 2.09 0.042 .0016369 .0860505

2016 | .0369274 .0193716 1.91 0.062 -.0019817 .0758365

2017 | -.006465 .0217974 -0.30 0.768 -.0502463 .0373163

2018 | .0135857 .0244795 0.55 0.581 -.0355828 .0627542

2019 | -.0080289 .0217825 -0.37 0.714 -.0517804 .0357226

|

statefip |

2 | -.1277315 .0056083 -22.78 0.000 -.1389961 -.1164668

4 | -.0410935 .0092835 -4.43 0.000 -.0597399 -.0224471

5 | -.0196373 .0067872 -2.89 0.006 -.0332699 -.0060048

6 | -.1283869 .008933 -14.37 0.000 -.1463293 -.1104445

8 | -.0731835 .0100074 -7.31 0.000 -.093284 -.0530829

9 | -.0138487 .0072674 -1.91 0.062 -.0284456 .0007482

10 | -.0317797 .0088235 -3.60 0.001 -.0495021 -.0140573

11 | -.0462857 .0052156 -8.87 0.000 -.0567616 -.0358099

12 | -.0230639 .0044823 -5.15 0.000 -.0320669 -.0140609

13 | -.0894545 .0023314 -38.37 0.000 -.0941372 -.0847718

15 | -.0618877 .0146953 -4.21 0.000 -.0914041 -.0323714

16 | -.0152526 .0069603 -2.19 0.033 -.0292328 -.0012723

17 | -.0254165 .0061893 -4.11 0.000 -.0378482 -.0129849

18 | -.0290825 .0054648 -5.32 0.000 -.0400589 -.018106

19 | .0186887 .0125256 1.49 0.142 -.0064697 .043847

20 | -.0360372 .0079401 -4.54 0.000 -.0519853 -.020089

21 | .0070239 .0052197 1.35 0.184 -.0034602 .0175079

22 | -.062754 .0044586 -14.07 0.000 -.0717094 -.0537987

23 | .103579 .0051565 20.09 0.000 .0932219 .1139362

24 | -.0877843 .0082761 -10.61 0.000 -.1044072 -.0711613

25 | .0046415 .0090299 0.51 0.610 -.0134957 .0227787

26 | .0374524 .0048029 7.80 0.000 .0278054 .0470993

27 | -.0255344 .0111273 -2.29 0.026 -.0478844 -.0031845

28 | -.0023737 .002507 -0.95 0.348 -.0074093 .0026618

29 | -.0145487 .0036652 -3.97 0.000 -.0219105 -.0071868

30 | -.0105811 .0096729 -1.09 0.279 -.0300096 .0088474

31 | -.037364 .0122627 -3.05 0.004 -.0619944 -.0127336

32 | -.1401656 .0079608 -17.61 0.000 -.1561553 -.1241759

33 | -.0645278 .0121112 -5.33 0.000 -.0888538 -.0402019

34 | -.1045422 .007418 -14.09 0.000 -.1194418 -.0896426

35 | .003081 .0100256 0.31 0.760 -.017056 .023218

36 | -.0143322 .0071982 -1.99 0.052 -.0287901 .0001258

37 | -.0128765 .0017695 -7.28 0.000 -.0164307 -.0093223

38 | -.0801927 .016269 -4.93 0.000 -.1128699 -.0475155

39 | .0292985 .0061429 4.77 0.000 .0169602 .0416368

40 | -.0331897 .0076091 -4.36 0.000 -.048473 -.0179065

41 | .0685729 .0066148 10.37 0.000 .0552867 .081859

42 | -.0162617 .0060228 -2.70 0.009 -.0283589 -.0041646

44 | .0671538 .0066788 10.05 0.000 .0537391 .0805685

45 | -.0151693 .0005609 -27.05 0.000 -.0162958 -.0140428

46 | .0307634 .0120451 2.55 0.014 .0065701 .0549568

47 | .0327752 .0022506 14.56 0.000 .0282548 .0372957

48 | -.0365815 .0086102 -4.25 0.000 -.0538757 -.0192874

49 | -.0997423 .0103595 -9.63 0.000 -.1205499 -.0789347

50 | .0337925 .0126519 2.67 0.010 .0083803 .0592046

51 | -.0860183 .0067458 -12.75 0.000 -.0995677 -.072469

53 | .0064478 .0069039 0.93 0.355 -.0074191 .0203147

54 | .0699348 .0053328 13.11 0.000 .0592236 .0806459

55 | .0388877 .005254 7.40 0.000 .0283348 .0494405

56 | -.0862588 .0081203 -10.62 0.000 -.1025689 -.0699487

|

male | -.0231722 .001696 -13.66 0.000 -.0265787 -.0197658

age | -.0007022 .0002388 -2.94 0.005 -.0011818 -.0002227

age2 | 6.02e-06 5.03e-06 1.20 0.237 -4.08e-06 .0000161

race_w | -.0861727 .0104463 -8.25 0.000 -.1071547 -.0651907

race_b | .0396531 .0114028 3.48 0.001 .0167499 .0625563

race_n | -.0197996 .0165282 -1.20 0.237 -.0529974 .0133982

race_a | -.13819 .0147716 -9.36 0.000 -.1678596 -.1085204

race_p | -.0482191 .0358276 -1.35 0.184 -.120181 .0237427

latino | -.012743 .0138933 -0.92 0.363 -.0406486 .0151625

married | -.039272 .0054449 -7.21 0.000 -.0502084 -.0283356

famsize | -.0238315 .0025766 -9.25 0.000 -.0290068 -.0186562

fpl_pct | .0042301 .0001884 22.46 0.000 .0038518 .0046084

fpl_pct2 | -.00004 1.33e-06 -30.04 0.000 -.0000426 -.0000373

hhkids | .0742164 .0045123 16.45 0.000 .0651533 .0832796

educ_1 | .2178286 .0102962 21.16 0.000 .197148 .2385092

educ_2 | .1653186 .0093692 17.64 0.000 .1464999 .1841373

educ_3 | .1298589 .0089869 14.45 0.000 .1118083 .1479095

unempl | .0040995 .0031507 1.30 0.199 -.0022288 .0104279

abawdwaive | .0152186 .0063173 2.41 0.020 .0025298 .0279073

_cons | .251179 .0366798 6.85 0.000 .1775055 .3248525

------------------------------------------------------------------------------

**Difference-in-differences in households with children full regression results**

Linear regression Number of obs = 306,533

F(28, 50) = .

Prob > F = .

R-squared = 0.1074

Root MSE = .472

(Std. Err. adjusted for 51 clusters in statefip)

------------------------------------------------------------------------------

| Robust

snap | Coef. Std. Err. t P>|t| [95% Conf. Interval]

-------------+----------------------------------------------------------------

medicaid expansion| .0237526 .0105996 2.24 0.030 .0024626 .0450426

|

year |

2011 | .0366837 .0115254 3.18 0.003 .0135343 .0598332

2012 | .0448179 .012119 3.70 0.001 .0204761 .0691596

2013 | .0714821 .0142715 5.01 0.000 .0428169 .1001474

2014 | .0519132 .0182662 2.84 0.006 .0152245 .0886019

2015 | .0474487 .0243954 1.94 0.057 -.0015508 .0964482

2016 | .0282965 .0224601 1.26 0.214 -.016816 .0734089

2017 | -.022363 .0249376 -0.90 0.374 -.0724517 .0277256

2018 | -.0007234 .0287197 -0.03 0.980 -.0584086 .0569618

2019 | -.019157 .027693 -0.69 0.492 -.07478 .0364661

|

statefip |

2 | -.1424844 .0073859 -19.29 0.000 -.1573194 -.1276495

4 | -.0372642 .0112024 -3.33 0.002 -.0597648 -.0147636

5 | -.0263037 .007992 -3.29 0.002 -.042356 -.0102513

6 | -.1156225 .0113448 -10.19 0.000 -.1384092 -.0928357

8 | -.0701733 .0112773 -6.22 0.000 -.0928245 -.0475222

9 | -.0625621 .0081698 -7.66 0.000 -.0789716 -.0461526

10 | -.0382103 .0106955 -3.57 0.001 -.0596928 -.0167278

11 | -.0533532 .0063775 -8.37 0.000 -.0661628 -.0405437

12 | -.0310365 .0041827 -7.42 0.000 -.0394377 -.0226353

13 | -.1018104 .0038748 -26.27 0.000 -.1095933 -.0940276

15 | -.0742516 .0178011 -4.17 0.000 -.1100061 -.0384971

16 | -.005968 .0082292 -0.73 0.472 -.0224967 .0105608

17 | -.018716 .0080427 -2.33 0.024 -.0348702 -.0025617

18 | -.0397535 .0061232 -6.49 0.000 -.0520523 -.0274546

19 | -.00837 .015423 -0.54 0.590 -.039348 .0226079

20 | -.0625862 .0096921 -6.46 0.000 -.0820534 -.043119

21 | -.0050654 .0069008 -0.73 0.466 -.018926 .0087952

22 | -.0732354 .0059078 -12.40 0.000 -.0851015 -.0613692

23 | .0970095 .0069407 13.98 0.000 .0830687 .1109503

24 | -.1000527 .0099392 -10.07 0.000 -.1200162 -.0800892

25 | -.0137257 .0103175 -1.33 0.189 -.034449 .0069975

26 | .0178974 .0066197 2.70 0.009 .0046014 .0311935

27 | -.0390924 .0138762 -2.82 0.007 -.0669636 -.0112211

28 | .0000755 .003287 0.02 0.982 -.0065267 .0066777

29 | -.0309303 .004744 -6.52 0.000 -.0404589 -.0214016

30 | -.0205608 .0123371 -1.67 0.102 -.0453406 .0042189

31 | -.0451066 .0157547 -2.86 0.006 -.0767507 -.0134624

32 | -.1551037 .011029 -14.06 0.000 -.1772562 -.1329513

33 | -.1042271 .01517 -6.87 0.000 -.134697 -.0737571

34 | -.1285028 .0077406 -16.60 0.000 -.1440502 -.1129553

35 | .0192944 .011491 1.68 0.099 -.003786 .0423747

36 | -.0300885 .0078355 -3.84 0.000 -.0458266 -.0143505

37 | -.0159916 .0024169 -6.62 0.000 -.020846 -.0111372

38 | -.1028948 .0208772 -4.93 0.000 -.1448279 -.0609617

39 | .0205439 .007339 2.80 0.007 .0058032 .0352847

40 | -.0513903 .0094605 -5.43 0.000 -.0703922 -.0323884

41 | .0517227 .0087231 5.93 0.000 .0342018 .0692436

42 | -.0518399 .0075009 -6.91 0.000 -.066906 -.0367739

44 | .0396731 .0085564 4.64 0.000 .0224869 .0568592

45 | -.0342279 .0009089 -37.66 0.000 -.0360535 -.0324024

46 | .0173675 .015709 1.11 0.274 -.0141851 .04892

47 | .0211933 .0030662 6.91 0.000 .0150347 .0273519

48 | -.0388903 .0098247 -3.96 0.000 -.0586238 -.0191569

49 | -.1168112 .0120607 -9.69 0.000 -.1410359 -.0925866

50 | .0221891 .0159292 1.39 0.170 -.0098056 .0541838

51 | -.0990114 .0088108 -11.24 0.000 -.1167085 -.0813143

53 | -.0124686 .0084393 -1.48 0.146 -.0294195 .0044823

54 | .0386284 .0075402 5.12 0.000 .0234836 .0537733

55 | .0305247 .0064193 4.76 0.000 .0176311 .0434182

56 | -.1018749 .0102853 -9.90 0.000 -.1225334 -.0812163

|

male | -.0156749 .00203 -7.72 0.000 -.0197523 -.0115975

age | -.0006351 .0002811 -2.26 0.028 -.0011996 -.0000706

age2 | 5.94e-06 5.36e-06 1.11 0.273 -4.82e-06 .0000167

race_w | -.0815593 .0116017 -7.03 0.000 -.1048621 -.0582566

race_b | .0479068 .0132603 3.61 0.001 .0212727 .0745409

race_n | -.023486 .0171317 -1.37 0.177 -.057896 .010924

race_a | -.1330658 .0156557 -8.50 0.000 -.1645113 -.1016204

race_p | -.0447082 .0450995 -0.99 0.326 -.1352932 .0458767

latino | -.0205554 .0136418 -1.51 0.138 -.0479557 .0068449

married | -.0282574 .0053231 -5.31 0.000 -.0389492 -.0175657

famsize | -.0347076 .0024227 -14.33 0.000 -.0395737 -.0298415

fpl_pct | .0041599 .0001452 28.66 0.000 .0038683 .0044515

fpl_pct2 | -.0000426 1.02e-06 -41.68 0.000 -.0000447 -.0000406

hhkids | .0658148 .0055117 11.94 0.000 .0547442 .0768855

educ_1 | .2065576 .0086297 23.94 0.000 .1892243 .2238908

educ_2 | .1667224 .0107742 15.47 0.000 .1450818 .188363

educ_3 | .144283 .0095861 15.05 0.000 .1250288 .1635372

unempl | .002812 .0041922 0.67 0.505 -.0056082 .0112322

abawdwaive | .0120244 .010298 1.17 0.248 -.0086597 .0327084

_cons | .3696697 .0428735 8.62 0.000 .2835558 .4557836

------------------------------------------------------------------------------

**Difference-in-differences in households with NO children full regression results**

Linear regression Number of obs = 107,360

F(27, 50) = .

Prob > F = .

R-squared = 0.1111

Root MSE = .42219

(Std. Err. adjusted for 51 clusters in statefip)

------------------------------------------------------------------------------

| Robust

snap | Coef. Std. Err. t P>|t| [95% Conf. Interval]

-------------+----------------------------------------------------------------

medicaid expansion | .0353093 .0126327 2.80 0.007 .0099358 .0606827

|

year |

2011 | .0246338 .008552 2.88 0.006 .0074566 .0418109

2012 | .0345113 .0116117 2.97 0.005 .0111885 .0578342

2013 | .0456509 .0114575 3.98 0.000 .0226377 .068664

2014 | .0413374 .0170079 2.43 0.019 .0071759 .0754988

2015 | .0377328 .0206407 1.83 0.074 -.0037253 .0791909

2016 | .0583241 .0213247 2.74 0.009 .0154922 .1011559

2017 | .0334452 .0232385 1.44 0.156 -.0132307 .0801211

2018 | .0482111 .0258012 1.87 0.068 -.0036122 .1000344

2019 | .0170432 .0255126 0.67 0.507 -.0342004 .0682867

|

statefip |

2 | -.0602941 .0086329 -6.98 0.000 -.0776338 -.0429545

4 | -.006541 .0079779 -0.82 0.416 -.022565 .009483

5 | .0160738 .007756 2.07 0.043 .0004955 .0316521

6 | -.1214791 .0080502 -15.09 0.000 -.1376484 -.1053099

8 | -.0288759 .0094778 -3.05 0.004 -.0479126 -.0098392

9 | .0965382 .0076755 12.58 0.000 .0811215 .1119549

10 | .0046862 .0120299 0.39 0.699 -.0194766 .028849

11 | .0189242 .0081562 2.32 0.024 .002542 .0353063

12 | .0134369 .0050146 2.68 0.010 .0033647 .0235091

13 | -.0371521 .0042446 -8.75 0.000 -.0456777 -.0286265

15 | .0016385 .0158114 0.10 0.918 -.0301197 .0333966

16 | .000693 .0056179 0.12 0.902 -.0105908 .0119768

17 | -.0097974 .0077555 -1.26 0.212 -.0253748 .0057801

18 | .0034092 .0067626 0.50 0.616 -.0101739 .0169923

19 | .1147717 .0116577 9.85 0.000 .0913565 .1381869

20 | .0441547 .0076408 5.78 0.000 .0288077 .0595017

21 | .0373212 .0078854 4.73 0.000 .0214828 .0531596

22 | -.0300404 .0059552 -5.04 0.000 -.0420018 -.0180791

23 | .1352624 .0047419 28.53 0.000 .1257381 .1447868

24 | -.0401046 .0086527 -4.63 0.000 -.057484 -.0227251

25 | .0571398 .009252 6.18 0.000 .0385566 .0757231

26 | .0990277 .0084964 11.66 0.000 .0819622 .1160932

27 | .0441541 .0109638 4.03 0.000 .0221326 .0661757

28 | .005436 .0022306 2.44 0.018 .0009558 .0099162

29 | .042539 .0032895 12.93 0.000 .0359319 .0491461

30 | .042604 .012659 3.37 0.001 .0171777 .0680303

31 | .0106222 .0134193 0.79 0.432 -.0163314 .0375757

32 | -.0702852 .0088908 -7.91 0.000 -.0881429 -.0524275

33 | .0177392 .0128595 1.38 0.174 -.0080898 .0435683

34 | -.0356138 .0080201 -4.44 0.000 -.0517226 -.019505

35 | -.0081901 .0091659 -0.89 0.376 -.0266004 .0102201

36 | .041563 .0075684 5.49 0.000 .0263613 .0567646

37 | .0146765 .0017657 8.31 0.000 .0111299 .018223

38 | .0156552 .0163859 0.96 0.344 -.0172569 .0485673

39 | .0729685 .0079191 9.21 0.000 .0570625 .0888745

40 | .0254476 .0068614 3.71 0.001 .0116661 .039229

41 | .1427157 .0085009 16.79 0.000 .1256412 .1597903

42 | .0767594 .0067802 11.32 0.000 .0631409 .0903778

44 | .1307069 .0085324 15.32 0.000 .1135689 .1478448

45 | .0333871 .0010543 31.67 0.000 .0312695 .0355048

46 | .0920455 .0133606 6.89 0.000 .06521 .118881

47 | .0723928 .0028129 25.74 0.000 .066743 .0780427

48 | -.0090053 .0127181 -0.71 0.482 -.0345504 .0165397

49 | -.0084011 .0101315 -0.83 0.411 -.0287509 .0119487

50 | .0667433 .0130549 5.11 0.000 .0405218 .0929648

51 | -.0342657 .0068486 -5.00 0.000 -.0480215 -.0205099

53 | .0737139 .0083507 8.83 0.000 .056941 .0904869

54 | .1268224 .0086928 14.59 0.000 .1093625 .1442824

55 | .08799 .0046191 19.05 0.000 .0787123 .0972677

56 | -.0167722 .0089935 -1.86 0.068 -.0348362 .0012918

|

male | -.0215771 .0037208 -5.80 0.000 -.0290505 -.0141037

age | .0157439 .0015668 10.05 0.000 .0125969 .018891

age2 | -.0001479 .0000163 -9.10 0.000 -.0001806 -.0001153

race_w | -.086412 .015708 -5.50 0.000 -.1179626 -.0548615

race_b | .0145187 .0182302 0.80 0.430 -.0220979 .0511352

race_n | -.0204664 .0344327 -0.59 0.555 -.0896265 .0486936

race_a | -.1131995 .0187373 -6.04 0.000 -.1508344 -.0755646

race_p | -.012362 .0469235 -0.26 0.793 -.1066106 .0818866

latino | -.005015 .0188001 -0.27 0.791 -.042776 .0327461

married | -.1070893 .0095261 -11.24 0.000 -.1262231 -.0879555

famsize | .0151459 .0051812 2.92 0.005 .0047392 .0255527

fpl_pct | .0039773 .0005015 7.93 0.000 .00297 .0049845

fpl_pct2 | -.0000323 3.50e-06 -9.23 0.000 -.0000393 -.0000253

educ_1 | .2242434 .0177779 12.61 0.000 .1885355 .2599512

educ_2 | .1299285 .0093928 13.83 0.000 .1110624 .1487946

educ_3 | .090069 .0089025 10.12 0.000 .0721877 .1079503

unempl | .0055236 .0029484 1.87 0.067 -.0003984 .0114455

abawdwaive | .022979 .0114982 2.00 0.051 -.0001158 .0460738

_cons | -.2915034 .0591552 -4.93 0.000 -.41032 -.1726868

------------------------------------------------------------------------------

**Table 2**

**Difference-in-differences 75-138% FPL full regression results**

Linear regression Number of obs = 206,692

F(28, 50) = .

Prob > F = .

R-squared = 0.0829

Root MSE = .45832

(Std. Err. adjusted for 51 clusters in statefip)

------------------------------------------------------------------------------

| Robust

snap | Coef. Std. Err. t P>|t| [95% Conf. Interval]

-------------+----------------------------------------------------------------

medicaid expansion | .0240435 .0115103 2.09 0.042 .0009243 .0471626

|

year |

2011 | .0178531 .0125016 1.43 0.159 -.007257 .0429633

2012 | .0213569 .0151093 1.41 0.164 -.008991 .0517049

2013 | .0391407 .0134942 2.90 0.006 .0120367 .0662447

2014 | .032397 .0196508 1.65 0.105 -.0070728 .0718668

2015 | .0334822 .0250418 1.34 0.187 -.0168157 .0837801

2016 | .0260266 .0238159 1.09 0.280 -.021809 .0738622

2017 | -.0109424 .0264963 -0.41 0.681 -.0641618 .0422771

2018 | -.0033733 .0299685 -0.11 0.911 -.0635669 .0568202

2019 | -.0106575 .0290221 -0.37 0.715 -.0689502 .0476352

|

statefip |

2 | -.1454049 .0064048 -22.70 0.000 -.1582693 -.1325405

4 | -.0474227 .0087223 -5.44 0.000 -.064942 -.0299033

5 | -.0447351 .0068975 -6.49 0.000 -.0585891 -.0308811

6 | -.1415744 .0088655 -15.97 0.000 -.1593813 -.1237676

8 | -.0765685 .0094273 -8.12 0.000 -.0955037 -.0576333

9 | .0068123 .0067969 1.00 0.321 -.0068397 .0204642

10 | -.0141405 .0117899 -1.20 0.236 -.0378213 .0095403

11 | -.0919381 .0079581 -11.55 0.000 -.1079224 -.0759537

12 | -.0320856 .004747 -6.76 0.000 -.0416202 -.0225509

13 | -.1217591 .0038428 -31.68 0.000 -.1294777 -.1140406

15 | -.0669446 .0153863 -4.35 0.000 -.0978488 -.0360403

16 | -.0373956 .0067117 -5.57 0.000 -.0508765 -.0239148

17 | -.0439155 .0071135 -6.17 0.000 -.0582033 -.0296276

18 | -.0785455 .0056408 -13.92 0.000 -.0898754 -.0672156

19 | .017104 .012545 1.36 0.179 -.0080934 .0423014

20 | -.0605956 .0082165 -7.37 0.000 -.0770989 -.0440923

21 | -.0290375 .0059353 -4.89 0.000 -.040959 -.0171161

22 | -.0795641 .0051503 -15.45 0.000 -.0899088 -.0692195

23 | .1026502 .0054818 18.73 0.000 .0916398 .1136606

24 | -.0984199 .008255 -11.92 0.000 -.1150007 -.0818392

25 | .0069848 .008148 0.86 0.395 -.009381 .0233506

26 | .0345517 .0067378 5.13 0.000 .0210184 .0480849

27 | -.0363767 .0112663 -3.23 0.002 -.0590058 -.0137476

28 | -.0470103 .0025146 -18.70 0.000 -.0520609 -.0419597

29 | -.025371 .0034389 -7.38 0.000 -.0322782 -.0184637

30 | -.0196037 .01241 -1.58 0.120 -.04453 .0053225

31 | -.0821427 .0143823 -5.71 0.000 -.1110304 -.0532551

32 | -.167465 .0089415 -18.73 0.000 -.1854244 -.1495055

33 | -.0636976 .0134046 -4.75 0.000 -.0906216 -.0367737

34 | -.1075255 .007597 -14.15 0.000 -.1227845 -.0922665

35 | -.0114134 .0094903 -1.20 0.235 -.0304753 .0076484

36 | -.0115421 .0073009 -1.58 0.120 -.0262064 .0031223

37 | -.0295752 .0017827 -16.59 0.000 -.0331559 -.0259945

38 | -.0788896 .0169519 -4.65 0.000 -.1129384 -.0448407

39 | .0312751 .0071658 4.36 0.000 .0168822 .045668

40 | -.0897506 .0080587 -11.14 0.000 -.1059369 -.0735642

41 | .085306 .0062612 13.62 0.000 .07273 .097882

42 | -.0075309 .0054026 -1.39 0.169 -.0183822 .0033205

44 | .078516 .0072469 10.83 0.000 .0639602 .0930718

45 | -.0514923 .0009306 -55.33 0.000 -.0533614 -.0496232

46 | .0061725 .0141744 0.44 0.665 -.0222977 .0346427

47 | .0059653 .0019139 3.12 0.003 .0021212 .0098094

48 | -.0587151 .0110759 -5.30 0.000 -.0809616 -.0364685

49 | -.088409 .0125992 -7.02 0.000 -.1137153 -.0631027

50 | .0398333 .0132696 3.00 0.004 .0131806 .066486

51 | -.109247 .0072979 -14.97 0.000 -.1239052 -.0945888

53 | .0125642 .0066092 1.90 0.063 -.0007108 .0258392

54 | .043409 .0054243 8.00 0.000 .032514 .0543039

55 | .0376976 .0048752 7.73 0.000 .0279054 .0474898

56 | -.1079823 .0097112 -11.12 0.000 -.1274878 -.0884768

|

male | -.0187937 .0024641 -7.63 0.000 -.0237429 -.0138445

age | .0001716 .0002366 0.73 0.472 -.0003036 .0006468

age2 | -9.68e-07 4.87e-06 -0.20 0.843 -.0000108 8.82e-06

race_w | -.0919867 .0133045 -6.91 0.000 -.1187095 -.0652639

race_b | .0157583 .0141036 1.12 0.269 -.0125695 .0440861

race_n | -.0361754 .0175616 -2.06 0.045 -.0714489 -.0009018

race_a | -.1370775 .0199675 -6.87 0.000 -.1771835 -.0969716

race_p | -.0559781 .0380235 -1.47 0.147 -.1323506 .0203944

latino | -.0186461 .0152903 -1.22 0.228 -.0493576 .0120654

married | -.0345016 .0074922 -4.61 0.000 -.0495501 -.0194531

famsize | -.0273578 .0045603 -6.00 0.000 -.0365175 -.0181981

fpl_pct | -.0038713 .0013647 -2.84 0.007 -.0066123 -.0011303

fpl_pct2 | 1.78e-06 6.60e-06 0.27 0.788 -.0000115 .000015

hhkids | .065831 .005147 12.79 0.000 .055493 .076169

educ_1 | .1727102 .0114522 15.08 0.000 .1497078 .1957126

educ_2 | .1239493 .0083533 14.84 0.000 .1071713 .1407273

educ_3 | .113325 .0075984 14.91 0.000 .0980632 .1285868

unempl | .0028735 .0030977 0.93 0.358 -.0033484 .0090953

abawdwaive | .0131223 .010121 1.30 0.201 -.0072064 .0334509

_cons | .7054517 .0897617 7.86 0.000 .52516 .8857435

------------------------------------------------------------------------------

**Difference-in-differences with kids and 75-138% FPL full regression results**

Linear regression Number of obs = 149,764

F(28, 50) = .

Prob > F = .

R-squared = 0.0733

Root MSE = .47045

(Std. Err. adjusted for 51 clusters in statefip)

------------------------------------------------------------------------------

| Robust

snap | Coef. Std. Err. t P>|t| [95% Conf. Interval]

-------------+----------------------------------------------------------------

medicaid expansion | .0241521 .0129786 1.86 0.069 -.0019162 .0502203

|

year |

2011 | .0102663 .0150879 0.68 0.499 -.0200387 .0405712

2012 | .0082367 .0178857 0.46 0.647 -.0276877 .0441611

2013 | .0336703 .0185377 1.82 0.075 -.0035636 .0709043

2014 | .0208555 .0251242 0.83 0.410 -.029608 .0713191

2015 | .0198606 .0303777 0.65 0.516 -.0411548 .080876

2016 | .0032189 .028724 0.11 0.911 -.054475 .0609128

2017 | -.0445377 .0324934 -1.37 0.177 -.1098027 .0207272

2018 | -.039424 .03472 -1.14 0.262 -.1091611 .0303131

2019 | -.0424438 .0371589 -1.14 0.259 -.1170796 .0321919

|

statefip |

2 | -.1632529 .0075118 -21.73 0.000 -.1783407 -.1481651

4 | -.0556863 .0108177 -5.15 0.000 -.0774143 -.0339583

5 | -.0660026 .0081305 -8.12 0.000 -.0823332 -.0496719

6 | -.1300388 .0113873 -11.42 0.000 -.1529109 -.1071667

8 | -.0819928 .011039 -7.43 0.000 -.1041652 -.0598203

9 | -.0619602 .0077684 -7.98 0.000 -.0775635 -.0463569

10 | -.0091276 .0152956 -0.60 0.553 -.0398497 .0215944

11 | -.1601935 .0088884 -18.02 0.000 -.1780464 -.1423407

12 | -.0388474 .004509 -8.62 0.000 -.0479041 -.0297907

13 | -.1559537 .005672 -27.50 0.000 -.1673463 -.1445611

15 | -.0750501 .0192208 -3.90 0.000 -.1136562 -.036444

16 | -.0527488 .0084118 -6.27 0.000 -.0696444 -.0358532

17 | -.0494879 .0085831 -5.77 0.000 -.0667275 -.0322484

18 | -.1015856 .0065217 -15.58 0.000 -.1146849 -.0884864

19 | -.0222541 .0161491 -1.38 0.174 -.0546906 .0101824

20 | -.1183675 .010472 -11.30 0.000 -.1394012 -.0973339

21 | -.0600856 .0070131 -8.57 0.000 -.0741717 -.0459994

22 | -.1088481 .005534 -19.67 0.000 -.1199635 -.0977328

23 | .0831441 .0077338 10.75 0.000 .0676104 .0986778

24 | -.1260585 .0090316 -13.96 0.000 -.1441991 -.1079179

25 | -.0262169 .0100026 -2.62 0.012 -.0463077 -.0061261

26 | .0008156 .0077931 0.10 0.917 -.0148373 .0164684

27 | -.0676195 .0146517 -4.62 0.000 -.0970483 -.0381908

28 | -.0578756 .0036112 -16.03 0.000 -.065129 -.0506222

29 | -.0511065 .0047093 -10.85 0.000 -.0605654 -.0416476

30 | -.025014 .0165926 -1.51 0.138 -.0583412 .0083131

31 | -.099525 .0186642 -5.33 0.000 -.1370132 -.0620368

32 | -.1970646 .0117435 -16.78 0.000 -.2206522 -.173477

33 | -.1029649 .0175831 -5.86 0.000 -.1382816 -.0676483

34 | -.1498604 .008339 -17.97 0.000 -.1666097 -.133111

35 | .0004893 .011583 0.04 0.966 -.0227758 .0237543

36 | -.0457184 .0081898 -5.58 0.000 -.062168 -.0292687

37 | -.0445351 .0026843 -16.59 0.000 -.0499267 -.0391435

38 | -.1122266 .022953 -4.89 0.000 -.158329 -.0661243

39 | .0222833 .0092809 2.40 0.020 .0036422 .0409245

40 | -.126234 .0108184 -11.67 0.000 -.1479634 -.1045047

41 | .0602888 .0080182 7.52 0.000 .0441838 .0763938

42 | -.0531761 .0065139 -8.16 0.000 -.0662595 -.0400926

44 | .040996 .0086521 4.74 0.000 .0236178 .0583741

45 | -.0890633 .0011311 -78.74 0.000 -.0913352 -.0867914

46 | -.0178293 .0193028 -0.92 0.360 -.0566002 .0209416

47 | -.0214253 .0031613 -6.78 0.000 -.0277749 -.0150758

48 | -.0733941 .0127774 -5.74 0.000 -.0990581 -.04773

49 | -.1183452 .0142895 -8.28 0.000 -.1470465 -.0896439

50 | .027432 .0176729 1.55 0.127 -.0080651 .0629291

51 | -.1345767 .0096929 -13.88 0.000 -.1540455 -.1151079

53 | -.0103617 .0077013 -1.35 0.185 -.0258303 .0051068

54 | -.0046686 .0068471 -0.68 0.498 -.0184214 .0090842

55 | .021821 .0067568 3.23 0.002 .0082496 .0353923

56 | -.1239899 .0125795 -9.86 0.000 -.1492565 -.0987233

|

male | -.0108911 .0031376 -3.47 0.001 -.0171931 -.0045891

age | -.0005792 .0003198 -1.81 0.076 -.0012215 .0000631

age2 | 9.72e-06 5.55e-06 1.75 0.086 -1.43e-06 .0000209

race_w | -.0886855 .0154307 -5.75 0.000 -.1196791 -.057692

race_b | .0217029 .0162647 1.33 0.188 -.0109658 .0543716

race_n | -.0326782 .0239634 -1.36 0.179 -.08081 .0154537

race_a | -.1358047 .022284 -6.09 0.000 -.1805634 -.0910461

race_p | -.0584745 .0437047 -1.34 0.187 -.1462579 .0293089

latino | -.0296382 .0156402 -1.90 0.064 -.0610524 .001776

married | -.0217684 .0065807 -3.31 0.002 -.0349862 -.0085506

famsize | -.0411455 .0035142 -11.71 0.000 -.048204 -.034087

fpl_pct | -.0037557 .0020009 -1.88 0.066 -.0077746 .0002632

fpl_pct2 | 2.26e-07 9.40e-06 0.02 0.981 -.0000186 .0000191

hhkids | .0635505 .0055259 11.50 0.000 .0524514 .0746496

educ_1 | .1530978 .0121779 12.57 0.000 .1286379 .1775577

educ_2 | .1206362 .01085 11.12 0.000 .0988433 .142429

educ_3 | .1208358 .0100597 12.01 0.000 .1006303 .1410413

unempl | .0005437 .004218 0.13 0.898 -.0079284 .0090159

abawdwaive | .0125926 .014005 0.90 0.373 -.0155372 .0407224

_cons | .8466396 .121196 6.99 0.000 .6032102 1.090069

------------------------------------------------------------------------------

**Difference-in-differences with NO kids and 75-138% FPL full regression results**

Linear regression Number of obs = 56,928

F(27, 50) = .

Prob > F = .

R-squared = 0.1063

Root MSE = .42254

(Std. Err. adjusted for 51 clusters in statefip)

------------------------------------------------------------------------------

| Robust

snap | Coef. Std. Err. t P>|t| [95% Conf. Interval]

-------------+----------------------------------------------------------------

medicaid expansion | .0215249 .0159467 1.35 0.183 -.0105051 .0535548

|

year |

2011 | .0349856 .010749 3.25 0.002 .0133957 .0565756

2012 | .0496997 .0152278 3.26 0.002 .0191138 .0802856

2013 | .0524423 .0131092 4.00 0.000 .0261116 .0787729

2014 | .0567147 .0170864 3.32 0.002 .0223957 .0910337

2015 | .0676601 .0213467 3.17 0.003 .024784 .1105361

2016 | .083485 .0251878 3.31 0.002 .0328939 .1340762

2017 | .0678788 .0242283 2.80 0.007 .0192148 .1165428

2018 | .0789606 .0295102 2.68 0.010 .0196877 .1382335

2019 | .0596564 .0259628 2.30 0.026 .0075085 .1118042

|

statefip |

2 | -.0839142 .0120345 -6.97 0.000 -.1080862 -.0597423

4 | -.0075051 .0106579 -0.70 0.485 -.0289122 .013902

5 | .0086394 .0092319 0.94 0.354 -.0099034 .0271823

6 | -.1426836 .0115498 -12.35 0.000 -.1658821 -.1194851

8 | -.0328278 .0101298 -3.24 0.002 -.0531742 -.0124815

9 | .1442253 .0105268 13.70 0.000 .1230817 .165369

10 | -.0099606 .0111912 -0.89 0.378 -.0324387 .0125175

11 | .0412781 .0098918 4.17 0.000 .0214099 .0611463

12 | -.0024178 .0049042 -0.49 0.624 -.0122682 .0074325

13 | -.0348506 .0051854 -6.72 0.000 -.0452657 -.0244355

15 | -.0242727 .0162051 -1.50 0.140 -.0568217 .0082762

16 | .0201937 .0057334 3.52 0.001 .0086778 .0317097

17 | -.0203056 .011177 -1.82 0.075 -.0427554 .0021441

18 | -.0263366 .0078571 -3.35 0.002 -.0421181 -.0105551

19 | .123867 .011677 10.61 0.000 .1004131 .1473208

20 | .0775853 .0080113 9.68 0.000 .0614942 .0936764

21 | .035437 .0113838 3.11 0.003 .012572 .058302

22 | -.0176788 .008407 -2.10 0.041 -.0345648 -.0007929

23 | .153837 .0052309 29.41 0.000 .1433304 .1643435

24 | -.0285871 .0114037 -2.51 0.015 -.0514922 -.0056821

25 | .0721406 .009623 7.50 0.000 .0528122 .0914691

26 | .1151085 .0119645 9.62 0.000 .0910772 .1391399

27 | .0562101 .0114214 4.92 0.000 .0332696 .0791505

28 | -.020778 .0022366 -9.29 0.000 -.0252703 -.0162856

29 | .0367129 .0031278 11.74 0.000 .0304306 .0429952

30 | .0309686 .0132887 2.33 0.024 .0042774 .0576597

31 | -.0029089 .0150511 -0.19 0.848 -.0331399 .027322

32 | -.0809172 .0122497 -6.61 0.000 -.1055214 -.0563131

33 | .0243334 .0125547 1.94 0.058 -.0008834 .0495503

34 | -.0096799 .0092936 -1.04 0.303 -.0283466 .0089868

35 | -.0186469 .0108142 -1.72 0.091 -.0403679 .0030742

36 | .0683827 .0094338 7.25 0.000 .0494343 .087331

37 | .0157464 .0016311 9.65 0.000 .0124704 .0190225

38 | .0262755 .0170451 1.54 0.129 -.0079606 .0605116

39 | .0706995 .0087661 8.07 0.000 .0530923 .0883067

40 | .0005166 .0075264 0.07 0.946 -.0146006 .0156337

41 | .1588437 .0116136 13.68 0.000 .1355171 .1821702

42 | .0927408 .0091622 10.12 0.000 .074338 .1111436

44 | .1493174 .0123563 12.08 0.000 .124499 .1741359

45 | .0218417 .001345 16.24 0.000 .0191401 .0245433

46 | .08778 .0155166 5.66 0.000 .0566141 .118946

47 | .0607378 .0033811 17.96 0.000 .0539466 .067529

48 | -.0078736 .0136403 -0.58 0.566 -.035271 .0195237

49 | .0109432 .0126687 0.86 0.392 -.0145026 .0363891

50 | .0868695 .0126304 6.88 0.000 .0615007 .1122384

51 | -.0441429 .0068295 -6.46 0.000 -.0578604 -.0304255

53 | .0794579 .0112054 7.09 0.000 .0569512 .1019647

54 | .1205177 .0125602 9.60 0.000 .0952899 .1457456

55 | .1001539 .0047193 21.22 0.000 .090675 .1096329

56 | -.0343668 .0103765 -3.31 0.002 -.0552086 -.0135251

|

male | -.0253089 .0037078 -6.83 0.000 -.0327562 -.0178615

age | .015346 .0015498 9.90 0.000 .012233 .0184589

age2 | -.0001403 .000015 -9.35 0.000 -.0001704 -.0001101

race_w | -.0935134 .0175543 -5.33 0.000 -.1287723 -.0582545

race_b | -.0015701 .0193195 -0.08 0.936 -.0403744 .0372342

race_n | -.0444871 .0414486 -1.07 0.288 -.1277391 .0387648

race_a | -.1225357 .0211747 -5.79 0.000 -.1650664 -.080005

race_p | -.0340454 .044786 -0.76 0.451 -.1240008 .0559099

latino | -.0017161 .0185361 -0.09 0.927 -.0389468 .0355147

married | -.0987595 .0135593 -7.28 0.000 -.1259942 -.0715249

famsize | .0123321 .0085047 1.45 0.153 -.0047501 .0294143

fpl_pct | -.003953 .0018301 -2.16 0.036 -.0076289 -.000277

fpl_pct2 | 3.56e-06 8.70e-06 0.41 0.684 -.0000139 .000021

educ_1 | .1957718 .0196781 9.95 0.000 .1562472 .2352964

educ_2 | .1047456 .0109206 9.59 0.000 .0828108 .1266803

educ_3 | .082581 .010481 7.88 0.000 .0615293 .1036326

unempl | .0075415 .0029339 2.57 0.013 .0016486 .0134344

abawdwaive | .0180584 .0131921 1.37 0.177 -.0084388 .0445556

_cons | .1407804 .1112036 1.27 0.211 -.0825787 .3641395

------------------------------------------------------------------------------

**Difference-in-differences under 75% FPL full regression results**

Linear regression Number of obs = 207,201

F(28, 50) = .

Prob > F = .

R-squared = 0.1513

Root MSE = .46019

(Std. Err. adjusted for 51 clusters in statefip)

------------------------------------------------------------------------------

| Robust

snap | Coef. Std. Err. t P>|t| [95% Conf. Interval]

-------------+----------------------------------------------------------------

medicaid expansion | .0332466 .0100529 3.31 0.002 .0130547 .0534385

|

year |

2011 | .0491666 .0107384 4.58 0.000 .0275979 .0707353

2012 | .0627731 .0117641 5.34 0.000 .0391442 .0864021

2013 | .0925369 .0164011 5.64 0.000 .0595944 .1254794

2014 | .065914 .0166672 3.95 0.000 .032437 .099391

2015 | .0563705 .0238379 2.36 0.022 .0084906 .1042504

2016 | .0517943 .0223808 2.31 0.025 .006841 .0967475

2017 | .0044378 .0240892 0.18 0.855 -.0439468 .0528224

2018 | .0365275 .0261475 1.40 0.169 -.0159913 .0890463

2019 | -.0015341 .0232978 -0.07 0.948 -.0483292 .0452609

|

statefip |

2 | -.1106221 .0085493 -12.94 0.000 -.1277939 -.0934504

4 | -.0316898 .0111588 -2.84 0.007 -.054103 -.0092767

5 | .0055988 .008103 0.69 0.493 -.0106766 .0218741

6 | -.1142182 .0115905 -9.85 0.000 -.1374985 -.090938

8 | -.0709561 .0113001 -6.28 0.000 -.093653 -.0482592

9 | -.0348102 .0092125 -3.78 0.000 -.053314 -.0163063

10 | -.0464676 .0089905 -5.17 0.000 -.0645255 -.0284097

11 | -.0086449 .0059162 -1.46 0.150 -.0205279 .0032381

12 | -.0145846 .0040282 -3.62 0.001 -.0226754 -.0064938

13 | -.0575204 .0031729 -18.13 0.000 -.0638933 -.0511475

15 | -.0531312 .0189009 -2.81 0.007 -.0910947 -.0151677

16 | .0150158 .0081872 1.83 0.073 -.0014287 .0314604

17 | -.0047533 .0078213 -0.61 0.546 -.0204629 .0109562

18 | .0137687 .0063708 2.16 0.035 .0009726 .0265648

19 | .0182215 .01575 1.16 0.253 -.0134133 .0498563

20 | -.016243 .0103793 -1.56 0.124 -.0370904 .0046043

21 | .0367678 .0073773 4.98 0.000 .0219501 .0515854

22 | -.045968 .0061435 -7.48 0.000 -.0583076 -.0336284

23 | .0986822 .0071421 13.82 0.000 .0843369 .1130275

24 | -.0696043 .0101813 -6.84 0.000 -.090054 -.0491546

25 | .0026136 .0108782 0.24 0.811 -.0192359 .0244631

26 | .0381576 .0066215 5.76 0.000 .0248579 .0514573

27 | -.0142741 .0134696 -1.06 0.294 -.0413286 .0127804

28 | .0391713 .0036735 10.66 0.000 .0317929 .0465498

29 | -.0084669 .0052805 -1.60 0.115 -.0190732 .0021393

30 | -.0066123 .0115093 -0.57 0.568 -.0297295 .0165048

31 | .0023936 .0162835 0.15 0.884 -.0303128 .0351

32 | -.1095719 .0115981 -9.45 0.000 -.1328674 -.0862763

33 | -.0694881 .0152042 -4.57 0.000 -.1000267 -.0389495

34 | -.0993947 .0086508 -11.49 0.000 -.1167705 -.082019

35 | .0105954 .0126167 0.84 0.405 -.0147459 .0359368

36 | -.0150678 .0079949 -1.88 0.065 -.031126 .0009904

37 | .0058176 .0023284 2.50 0.016 .0011409 .0104943

38 | -.0840022 .0217689 -3.86 0.000 -.1277263 -.0402781

39 | .0299756 .0065579 4.57 0.000 .0168037 .0431476

40 | .0232833 .0106266 2.19 0.033 .0019392 .0446274

41 | .049801 .0088964 5.60 0.000 .0319321 .0676698

42 | -.0236883 .0078578 -3.01 0.004 -.0394712 -.0079054

44 | .0536439 .0092871 5.78 0.000 .0349901 .0722976

45 | .0227612 .0010402 21.88 0.000 .020672 .0248504

46 | .0499149 .0172923 2.89 0.006 .0151823 .0846474

47 | .0547165 .003454 15.84 0.000 .0477788 .0616541

48 | -.0149751 .0094436 -1.59 0.119 -.0339431 .0039928

49 | -.11881 .0121868 -9.75 0.000 -.1432879 -.0943321

50 | .0198064 .0162131 1.22 0.228 -.0127586 .0523714

51 | -.0584762 .0087002 -6.72 0.000 -.075951 -.0410013

53 | .0036762 .0088604 0.41 0.680 -.0141204 .0214729

54 | .0930257 .0085438 10.89 0.000 .0758649 .1101865

55 | .038944 .0069121 5.63 0.000 .0250606 .0528274

56 | -.0650616 .0103897 -6.26 0.000 -.0859299 -.0441932

|

male | -.025103 .0022922 -10.95 0.000 -.0297069 -.020499

age | -.0010667 .0003284 -3.25 0.002 -.0017263 -.0004071

age2 | 5.42e-06 5.32e-06 1.02 0.313 -5.27e-06 .0000161

race_w | -.0753285 .0118821 -6.34 0.000 -.0991944 -.0514625

race_b | .0604602 .0134708 4.49 0.000 .0334033 .0875171

race_n | -.0015135 .0216842 -0.07 0.945 -.0450675 .0420405

race_a | -.1241407 .0158535 -7.83 0.000 -.1559834 -.0922981

race_p | -.0465468 .0430582 -1.08 0.285 -.1330318 .0399382

latino | -.001863 .0128941 -0.14 0.886 -.0277616 .0240357

married | -.0426653 .0054397 -7.84 0.000 -.0535913 -.0317394

famsize | -.0226097 .0024818 -9.11 0.000 -.0275945 -.0176249

fpl_pct | .007628 .0005096 14.97 0.000 .0066045 .0086515

fpl_pct2 | -.0000881 7.55e-06 -11.66 0.000 -.0001032 -.0000729

hhkids | .0797749 .0058202 13.71 0.000 .0680848 .0914651

educ_1 | .2495281 .011545 21.61 0.000 .2263393 .272717

educ_2 | .1987577 .0128077 15.52 0.000 .1730326 .2244827

educ_3 | .1392493 .0128382 10.85 0.000 .113463 .1650357

unempl | .0050119 .0047614 1.05 0.298 -.0045517 .0145754

abawdwaive | .0165822 .0096566 1.72 0.092 -.0028136 .035978

_cons | .1523657 .0486796 3.13 0.003 .0545899 .2501415

------------------------------------------------------------------------------

**Difference-in-differences with kids and under 75% FPL full regression results**

Linear regression Number of obs = 156,769

F(28, 50) = .

Prob > F = .

R-squared = 0.1092

Root MSE = .46899

(Std. Err. adjusted for 51 clusters in statefip)

------------------------------------------------------------------------------

| Robust

snap | Coef. Std. Err. t P>|t| [95% Conf. Interval]

-------------+----------------------------------------------------------------

medicaid expansion | .0239521 .0119855 2.00 0.051 -.0001215 .0480258

|

year |

2011 | .0614827 .0130654 4.71 0.000 .03524 .0877254

2012 | .0783035 .0133016 5.89 0.000 .0515865 .1050204

2013 | .1098281 .0178913 6.14 0.000 .0738924 .1457638

2014 | .0839541 .0200494 4.19 0.000 .0436836 .1242245

2015 | .077959 .0283706 2.75 0.008 .020975 .1349431

2016 | .060293 .0260225 2.32 0.025 .0080252 .1125608

2017 | .0079072 .0302115 0.26 0.795 -.0527744 .0685888

2018 | .0464397 .0316325 1.47 0.148 -.0170961 .1099755

2019 | .0110048 .0312188 0.35 0.726 -.0517 .0737097

|

statefip |

2 | -.1261544 .0104187 -12.11 0.000 -.1470809 -.1052278

4 | -.0217027 .0124463 -1.74 0.087 -.0467019 .0032965

5 | .0074349 .0087708 0.85 0.401 -.0101817 .0250515

6 | -.10516 .0139137 -7.56 0.000 -.1331064 -.0772135

8 | -.0649179 .0118881 -5.46 0.000 -.0887959 -.04104

9 | -.068071 .0094827 -7.18 0.000 -.0871175 -.0490245

10 | -.067874 .0101933 -6.66 0.000 -.0883478 -.0474002

11 | .0178778 .0080501 2.22 0.031 .0017087 .0340468

12 | -.0233705 .0039459 -5.92 0.000 -.031296 -.015445

13 | -.0536162 .0043589 -12.30 0.000 -.0623712 -.0448611

15 | -.06619 .0241724 -2.74 0.009 -.1147417 -.0176382

16 | .0499673 .0099695 5.01 0.000 .029943 .0699916

17 | .0085487 .0094553 0.90 0.370 -.0104428 .0275403

18 | .0099043 .0065314 1.52 0.136 -.0032145 .0230231

19 | -.0034524 .0189832 -0.18 0.856 -.0415814 .0346766

20 | -.0151769 .0133214 -1.14 0.260 -.0419337 .0115798

21 | .0349879 .0084107 4.16 0.000 .0180945 .0518813

22 | -.0432644 .007518 -5.75 0.000 -.0583649 -.028164

23 | .1019924 .0092238 11.06 0.000 .0834659 .1205189

24 | -.0676352 .0114355 -5.91 0.000 -.090604 -.0446664

25 | -.0027548 .0114834 -0.24 0.811 -.0258199 .0203104

26 | .0282752 .0088866 3.18 0.003 .0104259 .0461245

27 | -.0142092 .0165256 -0.86 0.394 -.0474018 .0189835

28 | .0487747 .0050762 9.61 0.000 .0385789 .0589706

29 | -.017101 .0069084 -2.48 0.017 -.030977 -.003225

30 | -.0190089 .0143696 -1.32 0.192 -.0478711 .0098533

31 | .0032363 .0219059 0.15 0.883 -.040763 .0472357

32 | -.1157326 .0155013 -7.47 0.000 -.1468678 -.0845974

33 | -.1122971 .0184185 -6.10 0.000 -.1492918 -.0753024

34 | -.1060243 .008594 -12.34 0.000 -.1232858 -.0887628

35 | .029552 .0134751 2.19 0.033 .0024864 .0566176

36 | -.0141145 .0078658 -1.79 0.079 -.0299135 .0016845

37 | .0123436 .0030591 4.03 0.000 .0061991 .0184881

38 | -.093713 .0276305 -3.39 0.001 -.1492104 -.0382155

39 | .0208644 .0068184 3.06 0.004 .0071692 .0345596

40 | .0191679 .0136509 1.40 0.166 -.0082509 .0465866

41 | .0327372 .0103937 3.15 0.003 .0118608 .0536136

42 | -.049934 .008636 -5.78 0.000 -.0672799 -.0325882

44 | .0338881 .011342 2.99 0.004 .0111071 .0566691

45 | .0161501 .0021995 7.34 0.000 .0117322 .020568

46 | .0459798 .0226532 2.03 0.048 .0004794 .0914802

47 | .0517191 .0041161 12.57 0.000 .0434518 .0599865

48 | -.0075618 .0119198 -0.63 0.529 -.0315033 .0163798

49 | -.1304143 .0156532 -8.33 0.000 -.1618547 -.0989739

50 | .012998 .0195454 0.67 0.509 -.0262601 .0522562

51 | -.0603433 .0114272 -5.28 0.000 -.0832955 -.037391

53 | -.0179508 .0095138 -1.89 0.065 -.0370598 .0011581

54 | .0717812 .0102821 6.98 0.000 .051129 .0924334

55 | .0345348 .0084084 4.11 0.000 .0176461 .0514235

56 | -.0820883 .0137566 -5.97 0.000 -.1097193 -.0544573

|

male | -.0183131 .0022491 -8.14 0.000 -.0228305 -.0137957

age | -.0006522 .0003086 -2.11 0.040 -.001272 -.0000325

age2 | 1.98e-06 5.77e-06 0.34 0.733 -9.61e-06 .0000136

race_w | -.0716193 .0138667 -5.16 0.000 -.0994713 -.0437673

race_b | .0667765 .0163208 4.09 0.000 .0339952 .0995579

race_n | -.0088486 .0219677 -0.40 0.689 -.0529719 .0352747

race_a | -.1166023 .0204871 -5.69 0.000 -.1577518 -.0754527

race_p | -.0404949 .0549298 -0.74 0.464 -.1508247 .0698349

latino | -.0049755 .0120939 -0.41 0.683 -.0292667 .0193158

married | -.0333717 .0056584 -5.90 0.000 -.0447368 -.0220065

famsize | -.032647 .0026549 -12.30 0.000 -.0379795 -.0273145

fpl_pct | .0096672 .0006259 15.45 0.000 .0084101 .0109243

fpl_pct2 | -.0001207 8.48e-06 -14.23 0.000 -.0001377 -.0001037

hhkids | .0673382 .0066664 10.10 0.000 .0539484 .0807281

educ_1 | .246738 .011526 21.41 0.000 .2235874 .2698886

educ_2 | .2055161 .0162301 12.66 0.000 .172917 .2381152

educ_3 | .160837 .0145442 11.06 0.000 .1316241 .1900498

unempl | .0053468 .006323 0.85 0.402 -.0073533 .0180469

abawdwaive | .0111928 .0129005 0.87 0.390 -.0147186 .0371043

_cons | .2094409 .059915 3.50 0.001 .0890981 .3297836

------------------------------------------------------------------------------

**Difference-in-differences with NO kids and under 75% FPL full regression results**

Linear regression Number of obs = 50,432

F(27, 50) = .

Prob > F = .

R-squared = 0.1238

Root MSE = .42034

(Std. Err. adjusted for 51 clusters in statefip)

------------------------------------------------------------------------------

| Robust

snap | Coef. Std. Err. t P>|t| [95% Conf. Interval]

-------------+----------------------------------------------------------------

medicaid expansion | .0499607 .0149642 3.34 0.002 .0199042 .0800173

|

year |

2011 | .0148956 .0138671 1.07 0.288 -.0129573 .0427485

2012 | .0201065 .0187694 1.07 0.289 -.0175929 .0578059

2013 | .040562 .0196126 2.07 0.044 .0011691 .079955

2014 | .0272843 .0225164 1.21 0.231 -.0179412 .0725098

2015 | .0083485 .0277642 0.30 0.765 -.0474176 .0641146

2016 | .0348749 .03192 1.09 0.280 -.0292383 .0989881

2017 | .001217 .0326842 0.04 0.970 -.0644312 .0668652

2018 | .0202054 .0341352 0.59 0.557 -.0483572 .088768

2019 | -.0232915 .03606 -0.65 0.521 -.0957201 .049137

|

statefip |

2 | -.033988 .010263 -3.31 0.002 -.0546019 -.0133742

4 | -.0074685 .0110703 -0.67 0.503 -.0297039 .0147668

5 | .0267664 .0106472 2.51 0.015 .0053808 .048152

6 | -.0994037 .0106229 -9.36 0.000 -.1207404 -.078067

8 | -.0254401 .0140482 -1.81 0.076 -.0536568 .0027765

9 | .0397357 .0105849 3.75 0.000 .0184752 .0609962

10 | .020063 .0154547 1.30 0.200 -.0109787 .0511046

11 | .0020254 .0099933 0.20 0.840 -.0180467 .0220974

12 | .0298998 .0055547 5.38 0.000 .0187429 .0410568

13 | -.0392772 .0057538 -6.83 0.000 -.0508341 -.0277204

15 | .0284285 .0220887 1.29 0.204 -.015938 .072795

16 | -.0209624 .0097528 -2.15 0.036 -.0405516 -.0013733

17 | .0023285 .0091211 0.26 0.800 -.0159919 .0206488

18 | .0321713 .0085928 3.74 0.000 .0149122 .0494304

19 | .1095566 .0185699 5.90 0.000 .0722578 .1468553

20 | .0057698 .0109689 0.53 0.601 -.0162619 .0278014

21 | .0361692 .0092703 3.90 0.000 .0175493 .054789

22 | -.0429796 .0076862 -5.59 0.000 -.0584179 -.0275413

23 | .1127181 .0079238 14.23 0.000 .0968027 .1286334

24 | -.0505362 .0119154 -4.24 0.000 -.0744691 -.0266033

25 | .0402774 .0135747 2.97 0.005 .0130118 .067543

26 | .0764732 .0085258 8.97 0.000 .0593486 .0935979

27 | .0325149 .0162241 2.00 0.050 -.0000721 .0651019

28 | .0318838 .0037285 8.55 0.000 .0243948 .0393728

29 | .0484437 .0058547 8.27 0.000 .0366841 .0602033

30 | .0571541 .0172884 3.31 0.002 .0224293 .0918788

31 | .0285779 .0195932 1.46 0.151 -.0107761 .067932

32 | -.0620793 .0104972 -5.91 0.000 -.0831635 -.0409951

33 | .0093492 .0188174 0.50 0.621 -.0284466 .0471451

34 | -.0666418 .0107029 -6.23 0.000 -.0881392 -.0451445

35 | -.0015357 .0142897 -0.11 0.915 -.0302374 .027166

36 | .0116313 .0105769 1.10 0.277 -.009613 .0328755

37 | .0135154 .0031698 4.26 0.000 .0071487 .0198821

38 | .0028752 .0245565 0.12 0.907 -.0464479 .0521983

39 | .0741341 .0100095 7.41 0.000 .0540295 .0942386

40 | .0549127 .0111668 4.92 0.000 .0324835 .0773419

41 | .1262976 .0096739 13.06 0.000 .106867 .1457282

42 | .0602072 .0089367 6.74 0.000 .0422572 .0781571

44 | .101837 .0103054 9.88 0.000 .081138 .122536

45 | .0447047 .0018826 23.75 0.000 .0409234 .0484859

46 | .1005374 .019315 5.21 0.000 .0617421 .1393327

47 | .0866509 .0045722 18.95 0.000 .0774675 .0958344

48 | -.0108477 .0142982 -0.76 0.452 -.0395665 .0178711

49 | -.034154 .0142803 -2.39 0.021 -.0628368 -.0054712

50 | .0408592 .0193697 2.11 0.040 .001954 .0797644

51 | -.0209648 .0099837 -2.10 0.041 -.0410177 -.000912

53 | .0663368 .0101292 6.55 0.000 .0459918 .0866819

54 | .1307354 .0099721 13.11 0.000 .110706 .1507649

55 | .0743225 .0078407 9.48 0.000 .058574 .090071

56 | .0089307 .0131839 0.68 0.501 -.0175499 .0354113

|

male | -.0159351 .005258 -3.03 0.004 -.0264961 -.005374

age | .0163902 .0018028 9.09 0.000 .0127692 .0200111

age2 | -.0001603 .0000198 -8.09 0.000 -.0002001 -.0001205

race_w | -.077429 .0228896 -3.38 0.001 -.1234042 -.0314539

race_b | .031313 .0245759 1.27 0.209 -.0180492 .0806751

race_n | .0035617 .0391692 0.09 0.928 -.075112 .0822353

race_a | -.100096 .0251168 -3.99 0.000 -.1505446 -.0496473

race_p | .0112386 .0756174 0.15 0.882 -.1406434 .1631206

latino | -.0078344 .0205294 -0.38 0.704 -.0490689 .0334002

married | -.1149231 .0092803 -12.38 0.000 -.133563 -.0962832

famsize | .0212243 .0050748 4.18 0.000 .0110312 .0314174

fpl_pct | .001921 .0006241 3.08 0.003 .0006674 .0031747

fpl_pct2 | -1.01e-06 8.70e-06 -0.12 0.908 -.0000185 .0000165

educ_1 | .2491969 .0159157 15.66 0.000 .2172293 .2811644

educ_2 | .1524929 .0093567 16.30 0.000 .1336994 .1712864

educ_3 | .0941158 .0101307 9.29 0.000 .0737678 .1144639

unempl | .003899 .0048083 0.81 0.421 -.0057588 .0135568

abawdwaive | .0285221 .0143139 1.99 0.052 -.0002283 .0572724

_cons | -.2990556 .0691843 -4.32 0.000 -.4380164 -.1600947

------------------------------------------------------------------------------

**Table 3**

**Difference-in-differences of SNAP benefit amount: full regression results**

Linear regression Number of obs = 510

F(12, 50) = .

Prob > F = .

R-squared = 0.9777

Root MSE = 6.9141

(Std. Err. adjusted for 51 clusters in statefip)

------------------------------------------------------------------------------

| Robust

amount | Coef. Std. Err. t P>|t| [95% Conf. Interval]

-------------+----------------------------------------------------------------

medicaid expansion | -.1501917 2.59728 -0.06 0.954 -5.366983 5.0666

maximum snap | .3166342 .1215778 2.60 0.012 .0724381 .5608303

unempl | 1.884654 .789834 2.39 0.021 .2982259 3.471083

abawdwaive | -.2766663 1.947913 -0.14 0.888 -4.189165 3.635833

|

year |

2011 | -2.430877 1.145337 -2.12 0.039 -4.731354 -.1304004

2012 | -5.623023 2.197681 -2.56 0.014 -10.03719 -1.208852

2013 | -7.642695 3.025985 -2.53 0.015 -13.72057 -1.564825

2014 | -15.13421 4.551633 -3.33 0.002 -24.27644 -5.991988

2015 | -13.95099 4.272654 -3.27 0.002 -22.53287 -5.369111

2016 | -17.26858 4.474972 -3.86 0.000 -26.25683 -8.280338

2017 | -20.04892 5.051139 -3.97 0.000 -30.19443 -9.903409

2018 | -23.37509 6.121859 -3.82 0.000 -35.6712 -11.07897

2019 | -26.88792 6.402654 -4.20 0.000 -39.74803 -14.02781

|

statefip |

2 | 86.57903 20.67461 4.19 0.000 45.05285 128.1052

4 | 4.786899 2.818784 1.70 0.096 -.8747949 10.44859

5 | -9.496406 2.00853 -4.73 0.000 -13.53066 -5.462154

6 | 27.66822 2.20109 12.57 0.000 23.2472 32.08924

8 | 15.24416 1.452123 10.50 0.000 12.32748 18.16083

9 | -7.531988 7.116625 -1.06 0.295 -21.82615 6.762174

10 | -8.314316 2.246972 -3.70 0.001 -12.82749 -3.801141

11 | -19.2145 7.962257 -2.41 0.020 -35.20716 -3.221831

12 | -10.26035 6.906986 -1.49 0.144 -24.13344 3.612737

13 | 12.81086 .7534593 17.00 0.000 11.2975 14.32423

15 | 114.0795 25.62318 4.45 0.000 62.61386 165.5452

16 | 2.692859 2.991849 0.90 0.372 -3.316446 8.702164

17 | 4.87891 3.554556 1.37 0.176 -2.260626 12.01845

18 | 9.951047 1.341666 7.42 0.000 7.256231 12.64586

19 | -23.83374 2.135022 -11.16 0.000 -28.12206 -19.54542

20 | -8.580166 1.738993 -4.93 0.000 -12.07304 -5.087297

21 | -8.394327 1.322843 -6.35 0.000 -11.05133 -5.73732

22 | 14.0915 1.058487 13.31 0.000 11.96547 16.21753

23 | -29.81905 6.274464 -4.75 0.000 -42.42168 -17.21641

24 | -17.70794 4.173672 -4.24 0.000 -26.09101 -9.324876

25 | -21.00539 8.247988 -2.55 0.014 -37.57196 -4.438815

26 | -14.41522 3.752007 -3.84 0.000 -21.95135 -6.879097

27 | -31.20075 4.222877 -7.39 0.000 -39.68265 -22.71885

28 | -8.164353 .9992824 -8.17 0.000 -10.17147 -6.157235

29 | 2.235707 2.415807 0.93 0.359 -2.616584 7.087998

30 | -6.318658 1.97648 -3.20 0.002 -10.28853 -2.34878

31 | -1.958442 2.536776 -0.77 0.444 -7.053706 3.136823

32 | -24.92838 2.710238 -9.20 0.000 -30.37206 -19.48471

33 | -32.89013 3.879138 -8.48 0.000 -40.68161 -25.09865

34 | -9.259114 3.850538 -2.40 0.020 -16.99315 -1.525081

35 | -.8286728 1.673288 -0.50 0.623 -4.189571 2.532225

36 | 9.02041 7.228844 1.25 0.218 -5.49915 23.53997

37 | -11.06783 1.715973 -6.45 0.000 -14.51447 -7.621197

38 | 7.396092 3.148406 2.35 0.023 1.072332 13.71985

39 | 1.453171 2.555544 0.57 0.572 -3.67979 6.586132

40 | 3.901429 1.439301 2.71 0.009 1.010509 6.79235

41 | -34.56263 6.405355 -5.40 0.000 -47.42816 -21.6971

42 | -11.69942 4.187005 -2.79 0.007 -20.10927 -3.289573

44 | -16.76678 7.804074 -2.15 0.037 -32.44173 -1.091835

45 | 1.188229 1.221904 0.97 0.336 -1.266038 3.642496

46 | 25.51199 2.585531 9.87 0.000 20.3188 30.70519

47 | 2.873843 3.674378 0.78 0.438 -4.506363 10.25405

48 | 12.85899 2.825469 4.55 0.000 7.183874 18.53412

49 | 15.43701 4.845571 3.19 0.002 5.704395 25.16963

50 | -19.26143 6.520583 -2.95 0.005 -32.35841 -6.164455

51 | -4.966761 2.590157 -1.92 0.061 -10.16924 .2357214

53 | -22.81479 6.844142 -3.33 0.002 -36.56165 -9.067927

54 | -28.337 2.842602 -9.97 0.000 -34.04653 -22.62746

55 | -35.36365 3.073248 -11.51 0.000 -41.53645 -29.19085

56 | 7.07238 2.44501 2.89 0.006 2.161434 11.98333

|

_cons | 146.3165 50.28595 2.91 0.005 45.31423 247.3188

------------------------------------------------------------------------------

**Difference-in-differences on households having $0 income: full regression results**

Linear regression Number of obs = 510

F(12, 50) = .

Prob > F = .

R-squared = 0.9386

Root MSE = 28055

(Std. Err. adjusted for 51 clusters in statefip)

------------------------------------------------------------------------------

| Robust

$0 income | Coef. Std. Err. t P>|t| [95% Conf. Interval]

-------------+----------------------------------------------------------------

postperiod | 15846.07 6694.702 2.37 0.022 2399.367 29292.78

maximum snap | -244.4773 158.5012 -1.54 0.129 -562.8364 73.88183

unempl | 6651.704 3621.443 1.84 0.072 -622.1772 13925.59

abawdwaive | 12693.51 9038.377 1.40 0.166 -5460.599 30847.63

|

year |

2011 | -7361.879 3469.468 -2.12 0.039 -14330.51 -393.2471

2012 | 4560.829 5160.483 0.88 0.381 -5804.307 14925.96

2013 | 13621.84 7506.181 1.81 0.076 -1454.771 28698.45

2014 | 9287.633 11300.06 0.82 0.415 -13409.22 31984.48

2015 | 14275.72 13650.91 1.05 0.301 -13142.94 41694.39

2016 | 6672.13 13300.04 0.50 0.618 -20041.78 33386.04

2017 | 3488.77 14146.3 0.25 0.806 -24924.92 31902.46

2018 | 434.9193 14798.56 0.03 0.977 -29288.87 30158.71

2019 | -28666.01 19199.36 -1.49 0.142 -67229.06 9897.042

|

statefip |

2 | -42930.72 24633.6 -1.74 0.088 -92408.76 6547.319

4 | 16739.67 6491.782 2.58 0.013 3700.543 29778.8

5 | -39362.99 4813.182 -8.18 0.000 -49030.55 -29695.43

6 | 368351.5 7783.648 47.32 0.000 352717.6 383985.4

8 | -41603.48 6105.281 -6.81 0.000 -53866.3 -29340.67

9 | -64687.01 13150 -4.92 0.000 -91099.55 -38274.46

10 | -64862.21 6655.847 -9.75 0.000 -78230.87 -51493.55

11 | -85013.67 13965.12 -6.09 0.000 -113063.4 -56963.89

12 | 350605.4 8923.178 39.29 0.000 332682.6 368528.1

13 | 118903.4 4003.688 29.70 0.000 110861.7 126945

15 | -5801.082 33969.1 -0.17 0.865 -74030.03 62427.87

16 | -48626.8 6625.222 -7.34 0.000 -61933.95 -35319.65

17 | 154905.2 10191.74 15.20 0.000 134434.5 175375.9

18 | -10877.49 3570.258 -3.05 0.004 -18048.56 -3706.41

19 | -39896.98 9696.406 -4.11 0.000 -59372.79 -20421.17

20 | -41199.83 7051.019 -5.84 0.000 -55362.22 -27037.45

21 | -10702.34 6509.045 -1.64 0.106 -23776.14 2371.463

22 | -11184.13 5435.381 -2.06 0.045 -22101.41 -266.8442

23 | -66924.12 8701.693 -7.69 0.000 -84401.98 -49446.25

24 | -21758.95 10200.97 -2.13 0.038 -42248.19 -1269.704

25 | -33629.15 13297.91 -2.53 0.015 -60338.79 -6919.509

26 | 62967.5 10536.51 5.98 0.000 41804.31 84130.7

27 | -53682.32 9733.828 -5.52 0.000 -73233.29 -34131.36

28 | -25057.59 2822.15 -8.88 0.000 -30726.04 -19389.13

29 | 2309.568 3858.261 0.60 0.552 -5439.978 10059.11

30 | -55481.76 8066.79 -6.88 0.000 -71684.39 -39279.14

31 | -35184.3 13365.26 -2.63 0.011 -62029.2 -8339.394

32 | -61924.61 11146.57 -5.56 0.000 -84313.15 -39536.08

33 | -66954.3 10928.24 -6.13 0.000 -88904.31 -45004.28

34 | -62072.83 7960.528 -7.80 0.000 -78062.02 -46083.64

35 | -54113.33 6478.126 -8.35 0.000 -67125.03 -41101.64

36 | 35437.03 12314.68 2.88 0.006 10702.27 60171.79

37 | 83824.72 2861.786 29.29 0.000 78076.65 89572.79

38 | -55138.56 14292.54 -3.86 0.000 -83845.96 -26431.16

39 | 63443.17 5644.532 11.24 0.000 52105.8 74780.55

40 | -5020.012 7338.014 -0.68 0.497 -19758.85 9718.823

41 | -20549.83 12983.85 -1.58 0.120 -46628.66 5529.013

42 | 21100.63 9049.406 2.33 0.024 2924.359 39276.89

44 | -98707.11 15318.47 -6.44 0.000 -129475.2 -67939.05

45 | 3867.243 1700.379 2.27 0.027 451.9312 7282.554

46 | -37812.44 13510.7 -2.80 0.007 -64949.47 -10675.41

47 | 65703.31 5358.307 12.26 0.000 54940.83 76465.78

48 | 291919.1 8969.658 32.55 0.000 273903 309935.2

49 | -28857.33 11673.79 -2.47 0.017 -52304.83 -5409.837

50 | -73528.8 12965.23 -5.67 0.000 -99570.23 -47487.38

51 | 4728.084 6797.529 0.70 0.490 -8925.155 18381.32

53 | 21.13924 13411.76 0.00 0.999 -26917.18 26959.46

54 | -67526.59 8969.939 -7.53 0.000 -85543.24 -49509.94

55 | -11040.8 5393.585 -2.05 0.046 -21874.13 -207.4637

56 | -53188.79 8609.107 -6.18 0.000 -70480.69 -35896.89

|

_cons | 116072.7 67211.56 1.73 0.090 -18925.69 251071.1

------------------------------------------------------------------------------

**Difference-in-differences on percent of SNAP households with ABAWD member: full regression results**

Linear regression Number of obs = 510

F(12, 50) = .

Prob > F = .

R-squared = 0.8505

Root MSE = 26744

(Std. Err. adjusted for 51 clusters in statefip)

------------------------------------------------------------------------------

| Robust

hhs w. abawd | Coef. Std. Err. t P>|t| [95% Conf. Interval]

-------------+----------------------------------------------------------------

medicaid expansion | 12207.33 7640.911 1.60 0.116 -3139.893 27554.55

maximum snap | -202.4278 160.307 -1.26 0.213 -524.4139 119.5582

unempl | 6230.835 3294.918 1.89 0.064 -387.2025 12848.87

abawdwaive | 11637.61 9130.808 1.27 0.208 -6702.162 29977.37

|

year |

2011 | 1285.506 2631.437 0.49 0.627 -3999.891 6570.903

2012 | 10514.26 5237.26 2.01 0.050 -5.08368 21033.61

2013 | 16877.86 7930.731 2.13 0.038 948.5185 32807.2

2014 | 10620.19 9790.313 1.08 0.283 -9044.229 30284.62

2015 | 18066.34 11819.5 1.53 0.133 -5673.829 41806.5

2016 | 9973.314 11427.79 0.87 0.387 -12980.08 32926.71

2017 | 6971.956 12139.87 0.57 0.568 -17411.7 31355.61

2018 | 2917.862 12727.01 0.23 0.820 -22645.08 28480.81

2019 | -9009.475 15265.37 -0.59 0.558 -39670.87 21651.92

|

statefip |

2 | -6738.377 23239.89 -0.29 0.773 -53417.07 39940.31

4 | -13351.68 6936.902 -1.92 0.060 -27284.86 581.4976

5 | -10191.45 3975.682 -2.56 0.013 -18176.84 -2206.058

6 | 272963.8 8912.356 30.63 0.000 255062.8 290864.8

8 | -23960.05 5281.148 -4.54 0.000 -34567.54 -13352.55

9 | -41963.67 14701.37 -2.85 0.006 -71492.25 -12435.09

10 | -25448.98 6445.73 -3.95 0.000 -38395.61 -12502.35

11 | -45787.18 16040.14 -2.85 0.006 -78004.75 -13569.61

12 | 178407.2 8937.462 19.96 0.000 160455.8 196358.6

13 | 60135.48 4202.333 14.31 0.000 51694.84 68576.11

15 | 25996.97 33778.64 0.77 0.445 -41849.42 93843.37

16 | -11623.32 6789.768 -1.71 0.093 -25260.97 2014.332

17 | 109172.3 11996.05 9.10 0.000 85077.56 133267.1

18 | -10403.16 3365.259 -3.09 0.003 -17162.48 -3643.836

19 | -15810.85 8019.874 -1.97 0.054 -31919.25 297.5386

20 | -13702.37 6645.064 -2.06 0.044 -27049.38 -355.3703

21 | -2093.32 7552.294 -0.28 0.783 -17262.55 13075.91

22 | 9947.423 5661.008 1.76 0.085 -1423.046 21317.89

23 | -25115.57 8063.123 -3.11 0.003 -41310.83 -8920.314

24 | -34639.7 10638.78 -3.26 0.002 -56008.32 -13271.08

25 | -26009.63 13716.99 -1.90 0.064 -53561.02 1541.762

26 | 36563.67 12420.95 2.94 0.005 11615.46 61511.87

27 | -18434.45 8767.757 -2.10 0.041 -36045.01 -823.8929

28 | -4158.548 2815.617 -1.48 0.146 -9813.881 1496.786

29 | -243.3874 3273.962 -0.07 0.941 -6819.334 6332.56

30 | -16388.24 7607.838 -2.15 0.036 -31669.03 -1107.447

31 | -1476.637 13535.72 -0.11 0.914 -28663.93 25710.66

32 | -55852.71 13162.28 -4.24 0.000 -82289.93 -29415.48

33 | -24235.48 9436.048 -2.57 0.013 -43188.34 -5282.62

34 | -35850.03 9371.1 -3.83 0.000 -54672.44 -17027.62

35 | -20300.08 7085.619 -2.86 0.006 -34531.97 -6068.196

36 | 28308.91 13617.38 2.08 0.043 957.6011 55660.22

37 | 35764.49 3059.183 11.69 0.000 29619.95 41909.04

38 | -12453.88 12517.83 -0.99 0.325 -37596.69 12688.93

39 | 27570.83 6511.474 4.23 0.000 14492.15 40649.51

40 | 1262.763 7269.165 0.17 0.863 -13337.78 15863.31

41 | 28042.87 14716.02 1.91 0.062 -1515.125 57600.86

42 | 17445.48 9927.721 1.76 0.085 -2494.933 37385.9

44 | -55362.46 17541.79 -3.16 0.003 -90596.18 -20128.74

45 | 1867.61 1767.125 1.06 0.296 -1681.765 5416.985

46 | 87.94173 13925.8 0.01 0.995 -27882.84 28058.73

47 | 22991 5193.553 4.43 0.000 12559.44 33422.56

48 | 34738.48 9735.473 3.57 0.001 15184.2 54292.75

49 | -4768.12 12388.24 -0.38 0.702 -29650.64 20114.4

50 | -28564.53 11655.35 -2.45 0.018 -51974.98 -5154.08

51 | 15478.16 5997.348 2.58 0.013 3432.13 27524.19

53 | 30435.78 15078.18 2.02 0.049 150.3722 60721.19

54 | -43513.01 10345.36 -4.21 0.000 -64292.28 -22733.74

55 | 5921.335 4519.073 1.31 0.196 -3155.489 14998.16

56 | -12767.3 9016.59 -1.42 0.163 -30877.65 5343.056

|

_cons | 53760.68 61641.12 0.87 0.387 -70049.16 177570.5

------------------------------------------------------------------------------
