# Supplementary material for: The Affordable Care Act Medicaid expansion: A difference-in-differences study of spillover participation in SNAP
Source: PLoS One. 2022 May 4;17(5):e0267244. doi: 10.1371/journal.pone.0267244 (PMC9067645; doi:10.1371/journal.pone.0267244)
Supplement: S3 Appendix — (DOCX) [file pone.0267244.s003.docx]

**S3 Appendix: Alternate Populations**

| **ACA Medicaid Expansion Effects on SNAP Food Assistance Receipt for Alternate Populations** | | | |
| --- | --- | --- | --- |
|  |  |  |  |
|  | **Low Education** | **Elderly (65+) HHs** | **Employer-insured** |
|  | **(any income)** | **<=138% FPL** | **<=138% FPL** |
| Effect of Medicaid Expansion | 0.023*** | 0.004 | 0.013 |
| Robust standard error | (0.005) | (0.013) | (0.011) |
| P-value | <0.001 | 0.782 | 0.255 |
| N | 730082 | 28272 | 83910 |
| Mean of dependent variable | 0.204 | 0.179 | 0.229 |

***p< 0.001 Data source is the Current Population Survey ASEC, 2011-2020 (reporting periods 2010-2019). All 50 states and DC are included. Regressions are linear probability models using survey weights. The unit of analysis is the individual. Standard errors in parentheses are robust to heteroskedasticity and clustered by state. About 18% of individuals in the employer-insured group report also having Medicaid in the previous year. Individuals in the low education group live in households where the head has at most a high school education.

**Sensitivity analysis: low education household head full regression results**

Linear regression Number of obs = 730,082

F(26, 50) = .

Prob > F = .

R-squared = 0.1765

Root MSE = .36592

(Std. Err. adjusted for 51 clusters in statefip)

------------------------------------------------------------------------------

| Robust

snap | Coef. Std. Err. t P>|t| [95% Conf. Interval]

-------------+----------------------------------------------------------------

medicaid expansion| .0232599 .0052594 4.42 0.000 .0126961 .0338237

|

year |

2011 | .0114623 .0047285 2.42 0.019 .0019648 .0209597

2012 | .0174613 .0062548 2.79 0.007 .0048982 .0300245

2013 | .0314131 .005856 5.36 0.000 .019651 .0431751

2014 | .0202736 .0091147 2.22 0.031 .0019661 .038581

2015 | .0179137 .0107344 1.67 0.101 -.0036469 .0394743

2016 | .0162331 .010407 1.56 0.125 -.0046698 .0371361

2017 | -.007585 .0126907 -0.60 0.553 -.0330749 .017905

2018 | .0022302 .0148064 0.15 0.881 -.0275093 .0319697

2019 | -.0059706 .0144973 -0.41 0.682 -.0350894 .0231481

|

statefip |

2 | -.0516385 .0033752 -15.30 0.000 -.0584179 -.0448592

4 | -.0005356 .0067686 -0.08 0.937 -.0141308 .0130595

5 | -.0142916 .0040389 -3.54 0.001 -.022404 -.0061793

6 | -.0613237 .0073615 -8.33 0.000 -.0761098 -.0465377

8 | -.026137 .006393 -4.09 0.000 -.0389777 -.0132964

9 | .0074664 .0040684 1.84 0.072 -.0007052 .0156379

10 | -.0279829 .0049416 -5.66 0.000 -.0379084 -.0180574

11 | .0180765 .0046624 3.88 0.000 .0087118 .0274412

12 | .0064251 .0033697 1.91 0.062 -.0003431 .0131934

13 | -.0295176 .0017094 -17.27 0.000 -.0329509 -.0260842

15 | -.0020916 .0101856 -0.21 0.838 -.02255 .0183668

16 | -.0111835 .0046264 -2.42 0.019 -.0204759 -.0018912

17 | -.0137375 .0042822 -3.21 0.002 -.0223386 -.0051364

18 | -.0209627 .0035626 -5.88 0.000 -.0281184 -.013807

19 | -.0055834 .0076424 -0.73 0.468 -.0209336 .0097668

20 | -.01621 .005258 -3.08 0.003 -.0267709 -.005649

21 | .0117246 .0037213 3.15 0.003 .0042502 .019199

22 | -.013164 .0023864 -5.52 0.000 -.0179573 -.0083707

23 | .0723852 .0039667 18.25 0.000 .0644178 .0803527

24 | -.0439624 .0038859 -11.31 0.000 -.0517675 -.0361574

25 | .0246988 .0048585 5.08 0.000 .0149403 .0344574

26 | .0303523 .0036044 8.42 0.000 .0231127 .0375919

27 | -.006574 .0064299 -1.02 0.312 -.0194888 .0063409

28 | .0215418 .0019389 11.11 0.000 .0176475 .0254362

29 | .000701 .0028721 0.24 0.808 -.0050679 .0064698

30 | -.0014249 .0062597 -0.23 0.821 -.0139979 .0111482

31 | -.0296493 .0084098 -3.53 0.001 -.0465409 -.0127577

32 | -.0726082 .0059707 -12.16 0.000 -.0846007 -.0606157

33 | -.0212591 .0078648 -2.70 0.009 -.0370561 -.0054621

34 | -.0536993 .0043949 -12.22 0.000 -.0625267 -.0448718

35 | .0260108 .0076555 3.40 0.001 .0106344 .0413873

36 | .0157263 .0040428 3.89 0.000 .0076061 .0238465

37 | .0039416 .0014629 2.69 0.010 .0010034 .0068799

38 | -.0294026 .009712 -3.03 0.004 -.0489098 -.0098955

39 | .0153551 .003816 4.02 0.000 .0076905 .0230198

40 | -.0175988 .0046068 -3.82 0.000 -.0268518 -.0083457

41 | .0698866 .0041792 16.72 0.000 .0614924 .0782807

42 | -.0045948 .0037269 -1.23 0.223 -.0120805 .0028908

44 | .0525778 .00441 11.92 0.000 .04372 .0614355

45 | -.0009419 .0006766 -1.39 0.170 -.002301 .0004172

46 | .010596 .007735 1.37 0.177 -.0049401 .0261321

47 | .0302299 .0016027 18.86 0.000 .0270107 .0334491

48 | -.0122081 .0072757 -1.68 0.100 -.0268218 .0024057

49 | -.0539094 .0069242 -7.79 0.000 -.0678171 -.0400017

50 | .0260674 .0081363 3.20 0.002 .0097251 .0424097

51 | -.043853 .0040875 -10.73 0.000 -.052063 -.0356429

53 | .0063862 .0042219 1.51 0.137 -.0020936 .0148661

54 | .041105 .0042097 9.76 0.000 .0326496 .0495604

55 | .0148234 .0036932 4.01 0.000 .0074054 .0222414

56 | -.0526376 .005525 -9.53 0.000 -.0637348 -.0415403

|

male | -.0193148 .0013468 -14.34 0.000 -.02202 -.0166096

age | .0014527 .0002762 5.26 0.000 .0008979 .0020075

age2 | -.0000235 3.93e-06 -5.98 0.000 -.0000314 -.0000156

race_w | -.0721963 .0111144 -6.50 0.000 -.0945202 -.0498724

race_b | .0485011 .0105674 4.59 0.000 .0272758 .0697264

race_n | -.0060899 .0127336 -0.48 0.635 -.0316661 .0194864

race_a | -.0844451 .011116 -7.60 0.000 -.1067723 -.0621179

race_p | -.0402075 .0261996 -1.53 0.131 -.092831 .012416

latino | .0015582 .0129437 0.12 0.905 -.02444 .0275564

married | -.0572044 .0040901 -13.99 0.000 -.0654196 -.0489893

famsize | -.0166009 .0022528 -7.37 0.000 -.0211259 -.012076

fpl_pct | -.0004246 .0000183 -23.18 0.000 -.0004614 -.0003878

fpl_pct2 | 4.81e-08 6.49e-09 7.40 0.000 3.50e-08 6.11e-08

hhkids | .0616519 .0033883 18.20 0.000 .0548464 .0684574

educ_1 | .0730681 .0060679 12.04 0.000 .0608805 .0852558

unempl | .002362 .0020703 1.14 0.259 -.0017963 .0065202

abawdwaive | .0053229 .0038349 1.39 0.171 -.0023797 .0130255

_cons | .3134621 .0228765 13.70 0.000 .2675133 .3594108

------------------------------------------------------------------------------

**Placebo test: low-income in all-senior households full regression results**

Linear regression Number of obs = 28,272

F(27, 50) = .

Prob > F = .

R-squared = 0.1194

Root MSE = .36011

(Std. Err. adjusted for 51 clusters in statefip)

------------------------------------------------------------------------------

| Robust

snap | Coef. Std. Err. t P>|t| [95% Conf. Interval]

-------------+----------------------------------------------------------------

medicaid expansion | .0036822 .0132469 0.28 0.782 -.022925 .0302895

|

year |

2011 | -.0087706 .0110185 -0.80 0.430 -.0309019 .0133606

2012 | -.0037025 .0143785 -0.26 0.798 -.0325826 .0251777

2013 | .0180742 .0140522 1.29 0.204 -.0101505 .0462988

2014 | .0099566 .0246191 0.40 0.688 -.0394924 .0594056

2015 | .008592 .0221815 0.39 0.700 -.0359608 .0531449

2016 | .0114162 .0271743 0.42 0.676 -.043165 .0659974

2017 | -.0012887 .0278488 -0.05 0.963 -.0572245 .0546472

2018 | .0055767 .0299992 0.19 0.853 -.0546784 .0658319

2019 | -.0069849 .0335003 -0.21 0.836 -.0742722 .0603023

|

statefip |

2 | -.0213767 .0105817 -2.02 0.049 -.0426307 -.0001227

4 | -.0080292 .0116794 -0.69 0.495 -.031488 .0154297

5 | -.0156886 .0096793 -1.62 0.111 -.03513 .0037527

6 | -.0765324 .0125291 -6.11 0.000 -.1016979 -.0513669

8 | .0032384 .0117454 0.28 0.784 -.0203529 .0268297

9 | .1907081 .0088777 21.48 0.000 .1728769 .2085394

10 | .0155786 .0137328 1.13 0.262 -.0120045 .0431617

11 | .0160435 .0095948 1.67 0.101 -.0032283 .0353152

12 | .0221449 .0064401 3.44 0.001 .0092096 .0350801

13 | .0088091 .0042571 2.07 0.044 .0002585 .0173598

15 | -.0106672 .0232354 -0.46 0.648 -.0573368 .0360025

16 | .0423471 .0078477 5.40 0.000 .0265844 .0581097

17 | .0856675 .0086648 9.89 0.000 .0682638 .1030712

18 | .0838037 .0076696 10.93 0.000 .068399 .0992085

19 | .0101652 .0162719 0.62 0.535 -.0225179 .0428483

20 | -.011399 .0095776 -1.19 0.240 -.0306362 .0078381

21 | .0091221 .0086591 1.05 0.297 -.0082701 .0265144

22 | -.0056246 .0065905 -0.85 0.397 -.0188621 .0076129

23 | .2084551 .0069227 30.11 0.000 .1945504 .2223598

24 | .0624569 .0098758 6.32 0.000 .0426207 .082293

25 | .1348593 .0105028 12.84 0.000 .1137639 .1559547

26 | .0860683 .008947 9.62 0.000 .0680978 .1040389

27 | .0507488 .0147519 3.44 0.001 .0211187 .0803789

28 | -.0250939 .0030497 -8.23 0.000 -.0312193 -.0189684

29 | -.0014023 .0048986 -0.29 0.776 -.0112415 .0084368

30 | .0197116 .0141184 1.40 0.169 -.008646 .0480693

31 | -.0310075 .0177344 -1.75 0.087 -.066628 .004613

32 | .0273424 .0127999 2.14 0.038 .0016329 .0530518

33 | .0661156 .0175663 3.76 0.000 .0308326 .1013987

34 | .0295611 .0093486 3.16 0.003 .0107839 .0483384

35 | -.0445183 .0162018 -2.75 0.008 -.0770605 -.011976

36 | .1740056 .0094764 18.36 0.000 .1549716 .1930396

37 | .001134 .0025117 0.45 0.654 -.0039108 .0061788

38 | .0232572 .0221674 1.05 0.299 -.0212672 .0677817

39 | .0767387 .0093253 8.23 0.000 .0580083 .0954691

40 | -.0110028 .0100205 -1.10 0.277 -.0311295 .0091239

41 | .1549502 .0106468 14.55 0.000 .1335654 .1763349

42 | .0625859 .0076065 8.23 0.000 .0473077 .0778641

44 | .1910858 .0097358 19.63 0.000 .1715308 .2106407

45 | -.0143125 .0016585 -8.63 0.000 -.0176437 -.0109814

46 | .033209 .0167822 1.98 0.053 -.000499 .066917

47 | .0484221 .0032358 14.96 0.000 .0419227 .0549215

48 | .0059827 .0101204 0.59 0.557 -.0143448 .0263101

49 | -.003535 .0124971 -0.28 0.778 -.0286361 .0215661

50 | .1517137 .0175369 8.65 0.000 .1164898 .1869376

51 | -.010398 .0096366 -1.08 0.286 -.0297536 .0089576

53 | .0796691 .0097628 8.16 0.000 .06006 .0992782

54 | .07963 .0087396 9.11 0.000 .0620759 .0971841

55 | .111412 .0063606 17.52 0.000 .0986364 .1241876

56 | -.0450034 .0105453 -4.27 0.000 -.0661841 -.0238226

|

male | .008035 .006722 1.20 0.238 -.0054666 .0215365

age | .0043367 .0178524 0.24 0.809 -.0315209 .0401943

age2 | -.0000732 .000117 -0.63 0.534 -.0003082 .0001618

race_w | -.0891796 .0317127 -2.81 0.007 -.1528764 -.0254828

race_b | -.0123553 .0339017 -0.36 0.717 -.0804488 .0557382

race_n | -.0782684 .0456389 -1.71 0.093 -.1699368 .0134

race_a | -.0294632 .0333243 -0.88 0.381 -.096397 .0374705

race_p | .0215435 .0818987 0.26 0.794 -.1429548 .1860418

latino | .1436656 .0408586 3.52 0.001 .0615988 .2257324

married | -.0305734 .015708 -1.95 0.057 -.0621238 .000977

famsize | -.0599529 .0175687 -3.41 0.001 -.0952408 -.0246651

fpl_pct | .0054577 .0006727 8.11 0.000 .0041065 .0068089

fpl_pct2 | -.0000388 4.75e-06 -8.18 0.000 -.0000483 -.0000293

educ_1 | .1063571 .0170512 6.24 0.000 .0721087 .1406054

educ_2 | .0226709 .0175524 1.29 0.202 -.0125842 .057926

educ_3 | .0188903 .0124107 1.52 0.134 -.0060373 .0438179

unempl | -.0062415 .0046144 -1.35 0.182 -.0155097 .0030267

abawdwaive | .0088714 .0111433 0.80 0.430 -.0135106 .0312534

_cons | .2612665 .6961377 0.38 0.709 -1.136967 1.6595

------------------------------------------------------------------------------

**Placebo test: low-income with employer insurance full regression results**

Linear regression Number of obs = 83,910

F(28, 50) = .

Prob > F = .

R-squared = 0.0739

Root MSE = .40459

(Std. Err. adjusted for 51 clusters in statefip)

------------------------------------------------------------------------------

| Robust

snap | Coef. Std. Err. t P>|t| [95% Conf. Interval]

-------------+----------------------------------------------------------------

medicaid expansion | .0132167 .011469 1.15 0.255 -.0098194 .0362529

|

year |

2011 | .0337708 .0145469 2.32 0.024 .0045526 .0629891

2012 | .0333558 .0121669 2.74 0.008 .0089179 .0577938

2013 | .0484414 .0149364 3.24 0.002 .0184407 .078442

2014 | .0460833 .0168986 2.73 0.009 .0121414 .0800251

2015 | .0481151 .0196697 2.45 0.018 .0086073 .0876228

2016 | .0433071 .0212028 2.04 0.046 .00072 .0858942

2017 | .0138637 .0242666 0.57 0.570 -.0348773 .0626047

2018 | .0359858 .0258116 1.39 0.169 -.0158584 .08783

2019 | .0115255 .0272733 0.42 0.674 -.0432546 .0663055

|

statefip |

2 | -.1104597 .0073011 -15.13 0.000 -.1251245 -.0957949

4 | -.0593033 .0078553 -7.55 0.000 -.0750812 -.0435254

5 | -.0300048 .0064699 -4.64 0.000 -.0429999 -.0170096

6 | -.1444069 .0094315 -15.31 0.000 -.1633506 -.1254633

8 | -.0686512 .0088203 -7.78 0.000 -.0863674 -.050935

9 | -.0896197 .0072857 -12.30 0.000 -.1042535 -.0749858

10 | -.0500634 .0105247 -4.76 0.000 -.0712028 -.028924

11 | -.0710174 .0093671 -7.58 0.000 -.0898318 -.052203

12 | -.0062364 .0022982 -2.71 0.009 -.0108525 -.0016202

13 | -.109981 .0033304 -33.02 0.000 -.1166702 -.1032918

15 | -.0843038 .0147597 -5.71 0.000 -.1139496 -.054658

16 | -.0423648 .0060888 -6.96 0.000 -.0545945 -.030135

17 | -.0743353 .0073749 -10.08 0.000 -.0891483 -.0595224

18 | -.0944875 .0050161 -18.84 0.000 -.1045627 -.0844123

19 | -.0099092 .0106301 -0.93 0.356 -.0312605 .011442

20 | -.0536358 .0074888 -7.16 0.000 -.0686774 -.0385941

21 | -.033414 .0064776 -5.16 0.000 -.0464247 -.0204034

22 | -.0680218 .005472 -12.43 0.000 -.0790126 -.057031

23 | .0263046 .0057407 4.58 0.000 .0147741 .037835

24 | -.1042136 .008898 -11.71 0.000 -.1220857 -.0863414

25 | -.0178552 .008408 -2.12 0.039 -.0347432 -.0009673

26 | .0044884 .0070612 0.64 0.528 -.0096945 .0186712

27 | -.0402898 .0097249 -4.14 0.000 -.0598228 -.0207568

28 | -.0378222 .0025322 -14.94 0.000 -.0429084 -.0327361

29 | -.0202317 .0039542 -5.12 0.000 -.028174 -.0122895

30 | -.0369171 .0098248 -3.76 0.000 -.0566508 -.0171834

31 | -.062277 .0118453 -5.26 0.000 -.0860691 -.038485

32 | -.1396439 .0096994 -14.40 0.000 -.1591257 -.1201622

33 | -.0763043 .0115793 -6.59 0.000 -.0995619 -.0530467

34 | -.1259023 .0080006 -15.74 0.000 -.141972 -.1098326

35 | -.0843966 .0088643 -9.52 0.000 -.1022011 -.0665921

36 | -.0766169 .0069418 -11.04 0.000 -.09056 -.0626738

37 | -.0326527 .0015556 -20.99 0.000 -.0357772 -.0295282

38 | -.0558909 .0141852 -3.94 0.000 -.0843827 -.0273992

39 | -.0292733 .0072549 -4.03 0.000 -.0438451 -.0147015

40 | -.0375598 .0077222 -4.86 0.000 -.0530702 -.0220494

41 | .0314108 .0072681 4.32 0.000 .0168123 .0460092

42 | -.0400611 .0061706 -6.49 0.000 -.052455 -.0276671

44 | .0451068 .0074698 6.04 0.000 .0301032 .0601103

45 | -.0156461 .0015095 -10.36 0.000 -.0186781 -.0126141

46 | .0088045 .0118111 0.75 0.459 -.0149189 .0325279

47 | -.0042823 .0031866 -1.34 0.185 -.0106827 .0021181

48 | -.0653648 .0082586 -7.91 0.000 -.0819528 -.0487769

49 | -.0395011 .0092928 -4.25 0.000 -.0581663 -.0208359

50 | .0252278 .0122489 2.06 0.045 .0006251 .0498305

51 | -.0955257 .0068228 -14.00 0.000 -.1092297 -.0818217

53 | -.0258066 .0072064 -3.58 0.001 -.0402811 -.011332

54 | -.0078899 .007083 -1.11 0.271 -.0221165 .0063368

55 | .0038057 .0050406 0.76 0.454 -.0063186 .0139301

56 | -.0869264 .0083072 -10.46 0.000 -.1036118 -.070241

|

male | .0013336 .0042276 0.32 0.754 -.0071577 .0098249

age | .0024032 .0003415 7.04 0.000 .0017173 .003089

age2 | -.0000235 4.67e-06 -5.02 0.000 -.0000329 -.0000141

race_w | -.0771884 .0194327 -3.97 0.000 -.11622 -.0381568

race_b | .018936 .0169642 1.12 0.270 -.0151376 .0530096

race_n | -.0296056 .0351545 -0.84 0.404 -.1002154 .0410043

race_a | -.0782468 .0187227 -4.18 0.000 -.1158524 -.0406412

race_p | -.0146694 .0322906 -0.45 0.652 -.0795271 .0501883

latino | .0251486 .0103265 2.44 0.018 .0044072 .0458899

married | -.0265925 .0056728 -4.69 0.000 -.0379867 -.0151983

famsize | -.0221432 .0047641 -4.65 0.000 -.0317121 -.0125743

fpl_pct | .0035466 .0002647 13.40 0.000 .003015 .0040782

fpl_pct2 | -.0000287 1.85e-06 -15.46 0.000 -.0000324 -.0000249

hhkids | .0599972 .0060034 9.99 0.000 .0479391 .0720554

educ_1 | .1366566 .0152857 8.94 0.000 .1059544 .1673588

educ_2 | .0913889 .01164 7.85 0.000 .0680092 .1147686

educ_3 | .0778419 .0121845 6.39 0.000 .0533687 .1023152

unempl | .0056104 .0036411 1.54 0.130 -.0017029 .0129237

abawdwaive | .0119786 .0107585 1.11 0.271 -.0096304 .0335876

_cons | .0772097 .0466402 1.66 0.104 -.0164699 .1708893

------------------------------------------------------------------------------
