# Supplementary material for: The Affordable Care Act Medicaid expansion: A difference-in-differences study of spillover participation in SNAP
Source: PLoS One. 2022 May 4;17(5):e0267244. doi: 10.1371/journal.pone.0267244 (PMC9067645; doi:10.1371/journal.pone.0267244)
Supplement: S4 Appendix — (DOCX) [file pone.0267244.s004.docx]

**S4 Appendix: Possible Mechanisms**

| **ACA Medicaid Expansion and Joint Processing Effects on SNAP Food Assistance Receipt by Family Type** | | | |
| --- | --- | --- | --- |
|  |  |  |  |
|  | **All HHs** | **HHs with** | **HHs with** |
|  |  | **Children** | **No Children** |
| States with NO Joint Processing | | | |
| Effect in states w/o Joint Processing | 0.028** | 0.030** | 0.020 |
| Robust standard error | (0.010) | (0.010) | (0.020) |
| P-value | 0.008 | 0.006 | 0.330 |
| States with Joint Processing | | | |
| Effect in states with Joint Processing | 0.030** | 0.025* | 0.036** |
| Robust standard error | (0.009) | (0.011) | (0.013) |
| P-value | 0.002 | 0.023 | 0.007 |
| N | 413893 | 306533 | 107360 |
| Mean of dependent variable | 0.416 | 0.478 | 0.277 |

*p<0.05 **p<0.01 ***p< 0.001 Data source is the Current Population Survey ASEC, 2011-2020 (reporting periods 2010-2019). All 50 states and DC are included. Triple differences regressions are linear probability models using survey weights. The unit of analysis is the individual, and stratification is by presence of children in the household. Standard errors in parentheses are robust to heteroskedasticity and clustered by state.

**Triple-differences for full population: full regression results**

Linear regression Number of obs = 413,893

F(30, 50) = .

Prob > F = .

R-squared = 0.1251

Root MSE = .46113

(Std. Err. adjusted for 51 clusters in statefip)

------------------------------------------------------------------------------

| Robust

snap | Coef. Std. Err. t P>|t| [95% Conf. Interval]

-------------+----------------------------------------------------------------

joint x expansion | .0026308 .0091946 0.29 0.776 -.0158372 .0210988

medicaid expansion | .0276817 .0099692 2.78 0.008 .007658 .0477055

joint processing | .013928 .0098929 1.41 0.165 -.0059426 .0337986

|

year |

2011 | .034497 .0090883 3.80 0.000 .0162427 .0527513

2012 | .0449982 .0101625 4.43 0.000 .0245863 .0654101

2013 | .0677777 .0108625 6.24 0.000 .0459597 .0895957

2014 | .0511681 .0154678 3.31 0.002 .0201 .0822362

2015 | .0471704 .0214602 2.20 0.033 .0040663 .0902745

2016 | .0406837 .0197824 2.06 0.045 .0009495 .0804178

2017 | -.0021046 .0224075 -0.09 0.926 -.0471114 .0429022

2018 | .0183201 .024885 0.74 0.465 -.0316629 .0683032

2019 | -.0031642 .0222797 -0.14 0.888 -.0479144 .0415859

|

statefip |

2 | -.1423855 .0112453 -12.66 0.000 -.1649724 -.1197986

4 | -.0558477 .0114154 -4.89 0.000 -.0787762 -.0329191

5 | -.0335612 .011023 -3.04 0.004 -.0557017 -.0114208

6 | -.143855 .0111537 -12.90 0.000 -.1662578 -.1214522

8 | -.0868447 .0121214 -7.16 0.000 -.1111911 -.0624982

9 | -.0283547 .0104299 -2.72 0.009 -.0493037 -.0074057

10 | -.0456822 .0126836 -3.60 0.001 -.071158 -.0202063

11 | -.0615354 .0108476 -5.67 0.000 -.0833234 -.0397473

12 | -.0368712 .0096136 -3.84 0.000 -.0561807 -.0175617

13 | -.1036096 .0096933 -10.69 0.000 -.1230792 -.08414

15 | -.0664723 .0166674 -3.99 0.000 -.0999498 -.0329948

16 | -.0281199 .01095 -2.57 0.013 -.0501136 -.0061261

17 | -.0404176 .010258 -3.94 0.000 -.0610213 -.0198138

18 | -.0432679 .0108282 -4.00 0.000 -.065017 -.0215188

19 | .006138 .0147809 0.42 0.680 -.0235504 .0358263

20 | -.0413546 .0086616 -4.77 0.000 -.058752 -.0239573

21 | -.0075557 .0105083 -0.72 0.475 -.0286621 .0135508

22 | -.0632341 .0045101 -14.02 0.000 -.072293 -.0541753

23 | .0905069 .0108549 8.34 0.000 .0687043 .1123095

24 | -.101462 .0112227 -9.04 0.000 -.1240035 -.0789204

25 | .0049661 .0089081 0.56 0.580 -.0129263 .0228586

26 | .0223054 .0105232 2.12 0.039 .0011689 .0434419

27 | -.0384311 .0137612 -2.79 0.007 -.0660712 -.010791

28 | -.0028879 .0026035 -1.11 0.273 -.0081172 .0023413

29 | -.0277979 .0101989 -2.73 0.009 -.048283 -.0073128

30 | -.0235562 .0136821 -1.72 0.091 -.0510376 .0039251

31 | -.0410988 .0125579 -3.27 0.002 -.0663221 -.0158754

32 | -.1561451 .0115008 -13.58 0.000 -.1792452 -.1330451

33 | -.0770842 .0146796 -5.25 0.000 -.106569 -.0475993

34 | -.1192713 .0107461 -11.10 0.000 -.1408554 -.0976872

35 | -.0112522 .0120185 -0.94 0.354 -.035392 .0128876

36 | -.0286427 .0105436 -2.72 0.009 -.0498201 -.0074654

37 | -.0205198 .005306 -3.87 0.000 -.0311771 -.0098625

38 | -.0918429 .0182796 -5.02 0.000 -.1285585 -.0551274

39 | .0148709 .0114627 1.30 0.200 -.0081525 .0378943

40 | -.0455391 .0117546 -3.87 0.000 -.0691491 -.0219292

41 | .0539244 .0104686 5.15 0.000 .0328977 .0749512

42 | -.0302901 .0104463 -2.90 0.006 -.0512722 -.009308

44 | .058796 .0071835 8.18 0.000 .0443675 .0732245

45 | -.0152851 .0005593 -27.33 0.000 -.0164084 -.0141618

46 | .019314 .0152891 1.26 0.212 -.0113951 .0500232

47 | .0224204 .007431 3.02 0.004 .0074948 .037346

48 | -.0496202 .01176 -4.22 0.000 -.073241 -.0259995

49 | -.1118967 .0133066 -8.41 0.000 -.1386237 -.0851697

50 | .0300943 .0143214 2.10 0.041 .0013289 .0588596

51 | -.0986303 .0108411 -9.10 0.000 -.1204052 -.0768554

53 | .000378 .0089283 0.04 0.966 -.017555 .018311

54 | .055512 .0106792 5.20 0.000 .0340622 .0769618

55 | .0259395 .0103188 2.51 0.015 .0052136 .0466654

56 | -.0913116 .0090217 -10.12 0.000 -.1094322 -.073191

|

male | -.0231764 .0016965 -13.66 0.000 -.0265839 -.019769

age | -.0007004 .0002392 -2.93 0.005 -.0011808 -.00022

age2 | 6.00e-06 5.03e-06 1.19 0.239 -4.10e-06 .0000161

race_w | -.0861071 .01044 -8.25 0.000 -.1070765 -.0651377

race_b | .0396882 .0114032 3.48 0.001 .0167842 .0625922

race_n | -.0197069 .0165145 -1.19 0.238 -.0528773 .0134634

race_a | -.1381072 .0147625 -9.36 0.000 -.1677585 -.1084559

race_p | -.0481724 .0358154 -1.35 0.185 -.1201098 .023765

latino | -.0127532 .0139089 -0.92 0.364 -.0406901 .0151836

married | -.0393013 .0054537 -7.21 0.000 -.0502554 -.0283473

famsize | -.023819 .0025761 -9.25 0.000 -.0289932 -.0186448

fpl_pct | .0042298 .0001885 22.44 0.000 .0038513 .0046084

fpl_pct2 | -.00004 1.33e-06 -30.02 0.000 -.0000426 -.0000373

hhkids | .0742152 .0045147 16.44 0.000 .0651471 .0832833

educ_1 | .217872 .0103108 21.13 0.000 .1971621 .2385818

educ_2 | .1653629 .0093848 17.62 0.000 .1465129 .1842129

educ_3 | .1298873 .0089885 14.45 0.000 .1118334 .1479412

unempl | .004883 .0031905 1.53 0.132 -.0015252 .0112912

abawdwaive | .0151004 .0062834 2.40 0.020 .0024799 .0277209

_cons | .2428654 .0370351 6.56 0.000 .1684783 .3172526

------------------------------------------------------------------------------

. lincom joint x post + medicaid expansion

( 1) joint x post + medicaid expansion = 0

------------------------------------------------------------------------------

snap | Coef. Std. Err. t P>|t| [95% Conf. Interval]

-------------+----------------------------------------------------------------

(1) | .0303125 .0091319 3.32 0.002 .0119706 .0486545

------------------------------------------------------------------------------

**Triple-differences for those in households with children: full regression results**

Linear regression Number of obs = 306,533

F(30, 50) = .

Prob > F = .

R-squared = 0.1075

Root MSE = .47199

(Std. Err. adjusted for 51 clusters in statefip)

------------------------------------------------------------------------------

| Robust

snap | Coef. Std. Err. t P>|t| [95% Conf. Interval]

-------------+----------------------------------------------------------------

joint x expansion | -.005072 .0105856 -0.48 0.634 -.0263338 .0161899

medicaid expansion | .0304285 .0104923 2.90 0.006 .0093542 .0515028

joint processing | .0264841 .0121906 2.17 0.035 .0019986 .0509697

|

year |

2011 | .0384301 .0115759 3.32 0.002 .0151792 .0616809

2012 | .0476871 .01191 4.00 0.000 .0237652 .071609

2013 | .0750992 .0138917 5.41 0.000 .0471968 .1030015

2014 | .0559863 .0182814 3.06 0.004 .0192671 .0927055

2015 | .0525926 .0247655 2.12 0.039 .0028496 .1023357

2016 | .033877 .0227311 1.49 0.142 -.0117797 .0795337

2017 | -.0158179 .0255615 -0.62 0.539 -.0671598 .035524

2018 | .0064528 .028938 0.22 0.824 -.0516708 .0645765

2019 | -.0118119 .0280698 -0.42 0.676 -.0681918 .044568

|

statefip |

2 | -.1698796 .014417 -11.78 0.000 -.1988371 -.1409221

4 | -.0646996 .0149868 -4.32 0.000 -.0948014 -.0345978

5 | -.0525172 .0138867 -3.78 0.000 -.0804096 -.0246249

6 | -.1441525 .0152165 -9.47 0.000 -.1747157 -.1135892

8 | -.0959752 .0151235 -6.35 0.000 -.1263517 -.0655987

9 | -.0896059 .0133466 -6.71 0.000 -.1164133 -.0627986

10 | -.0644806 .0161699 -3.99 0.000 -.0969589 -.0320023

11 | -.0815577 .0137653 -5.92 0.000 -.1092062 -.0539092

12 | -.0573553 .0120825 -4.75 0.000 -.0816237 -.0330869

13 | -.128569 .0120605 -10.66 0.000 -.1527933 -.1043447

15 | -.0883601 .0196511 -4.50 0.000 -.1278305 -.0488897

16 | -.0308852 .0136111 -2.27 0.028 -.0582239 -.0035465

17 | -.0465328 .0136179 -3.42 0.001 -.0738853 -.0191804

18 | -.0664238 .0135956 -4.89 0.000 -.0937313 -.0391164

19 | -.0325524 .0185589 -1.75 0.086 -.069829 .0047242

20 | -.0738614 .0106829 -6.91 0.000 -.0953186 -.0524042

21 | -.0322513 .0138379 -2.33 0.024 -.0600456 -.004457

22 | -.0775778 .0056427 -13.75 0.000 -.0889115 -.0662442

23 | .0718303 .0137565 5.22 0.000 .0441997 .0994609

24 | -.1258595 .013912 -9.05 0.000 -.1538026 -.0979163

25 | -.018135 .0103605 -1.75 0.086 -.0389448 .0026747

26 | -.0101395 .0138277 -0.73 0.467 -.0379133 .0176343

27 | -.0637943 .0173669 -3.67 0.001 -.0986768 -.0289119

28 | -.0006923 .0034478 -0.20 0.842 -.0076175 .0062329

29 | -.056395 .0125977 -4.48 0.000 -.0816982 -.0310918

30 | -.0455461 .0175202 -2.60 0.012 -.0807365 -.0103558

31 | -.053108 .0156628 -3.39 0.001 -.0845676 -.0216484

32 | -.1844394 .0159562 -11.56 0.000 -.2164883 -.1523905

33 | -.1284654 .018627 -6.90 0.000 -.1658789 -.0910519

34 | -.1559191 .0135062 -11.54 0.000 -.1830471 -.1287912

35 | -.0074683 .0153316 -0.49 0.628 -.0382627 .023326

36 | -.0568762 .0133037 -4.28 0.000 -.0835974 -.0301549

37 | -.0300269 .0066366 -4.52 0.000 -.0433569 -.016697

38 | -.1256925 .0233164 -5.39 0.000 -.1725248 -.0788602

39 | -.0065008 .0146919 -0.44 0.660 -.0360103 .0230087

40 | -.075507 .0144008 -5.24 0.000 -.1044319 -.0465821

41 | .0244343 .0141015 1.73 0.089 -.0038894 .0527579

42 | -.0782402 .0133426 -5.86 0.000 -.1050397 -.0514407

44 | .0254527 .0094251 2.70 0.009 .0065219 .0443835

45 | -.0344053 .0009424 -36.51 0.000 -.0362983 -.0325124

46 | -.0055211 .0190439 -0.29 0.773 -.0437718 .0327297

47 | .0011284 .0094305 0.12 0.905 -.0178133 .02007

48 | -.0641248 .0148336 -4.32 0.000 -.0939191 -.0343305

49 | -.1406598 .0159192 -8.84 0.000 -.1726345 -.1086851

50 | .009272 .017088 0.54 0.590 -.0250501 .0435942

51 | -.12347 .0135469 -9.11 0.000 -.1506797 -.0962603

53 | -.0287595 .0112367 -2.56 0.014 -.051329 -.00619

54 | .0117617 .0141459 0.83 0.410 -.0166512 .0401747

55 | .0055371 .0127389 0.43 0.666 -.0200498 .0311241

56 | -.1123061 .0112815 -9.95 0.000 -.1349657 -.0896466

|

male | -.0156854 .0020294 -7.73 0.000 -.0197615 -.0116092

age | -.0006328 .0002811 -2.25 0.029 -.0011974 -.0000682

age2 | 5.92e-06 5.35e-06 1.11 0.274 -4.83e-06 .0000167

race_w | -.0814473 .0116046 -7.02 0.000 -.1047557 -.0581388

race_b | .0479447 .0132695 3.61 0.001 .0212921 .0745973

race_n | -.0232865 .0171077 -1.36 0.180 -.0576483 .0110753

race_a | -.1329269 .0156329 -8.50 0.000 -.1643264 -.1015273

race_p | -.0447393 .0450335 -0.99 0.325 -.1351917 .0457131

latino | -.0205871 .0136572 -1.51 0.138 -.0480184 .0068442

married | -.0283026 .005323 -5.32 0.000 -.0389941 -.0176111

famsize | -.0346813 .0024187 -14.34 0.000 -.0395394 -.0298233

fpl_pct | .0041586 .0001456 28.57 0.000 .0038662 .004451

fpl_pct2 | -.0000426 1.03e-06 -41.56 0.000 -.0000447 -.0000406

hhkids | .0658266 .0055157 11.93 0.000 .0547479 .0769052

educ_1 | .2066261 .0086326 23.94 0.000 .189287 .2239651

educ_2 | .1668178 .0107794 15.48 0.000 .1451667 .1884689

educ_3 | .1443727 .0095845 15.06 0.000 .1251217 .1636238

unempl | .0040024 .0042447 0.94 0.350 -.0045234 .0125282

abawdwaive | .0116832 .0101965 1.15 0.257 -.0087971 .0321636

_cons | .3570437 .0426164 8.38 0.000 .2714461 .4426413

------------------------------------------------------------------------------

. lincom joint x expansion + medicaid expansion

( 1) joint x expansion + medicaid expansion = 0

------------------------------------------------------------------------------

snap | Coef. Std. Err. t P>|t| [95% Conf. Interval]

-------------+----------------------------------------------------------------

(1) | .0253565 .010828 2.34 0.023 .0036078 .0471053

------------------------------------------------------------------------------

**Triple-differences for those in households with NO children: full regression results**

Linear regression Number of obs = 107,360

F(29, 50) = .

Prob > F = .

R-squared = 0.1111

Root MSE = .42219

(Std. Err. adjusted for 51 clusters in statefip)

------------------------------------------------------------------------------

| Robust

snap | Coef. Std. Err. t P>|t| [95% Conf. Interval]

-------------+----------------------------------------------------------------

joint x expansion | .0162986 .0188433 0.86 0.391 -.0215493 .0541466

medicaid expansion | .019821 .020164 0.98 0.330 -.0206797 .0603216

joint processing | -.0149091 .0101956 -1.46 0.150 -.0353876 .0055694

|

year |

2011 | .023918 .0086146 2.78 0.008 .006615 .041221

2012 | .0337214 .0116375 2.90 0.006 .0103467 .057096

2013 | .044772 .0117583 3.81 0.000 .0211548 .0683891

2014 | .0406107 .0171915 2.36 0.022 .0060805 .0751409

2015 | .0368349 .021027 1.75 0.086 -.005399 .0790689

2016 | .0578089 .0216735 2.67 0.010 .0142764 .1013414

2017 | .0325349 .0236488 1.38 0.175 -.0149651 .080035

2018 | .0472696 .026227 1.80 0.078 -.0054089 .0999481

2019 | .0159899 .0261117 0.61 0.543 -.0364571 .0684369

|

statefip |

2 | -.0458623 .0130857 -3.50 0.001 -.0721456 -.0195789

4 | .0078433 .0125965 0.62 0.536 -.0174575 .0331441

5 | .0304552 .0123706 2.46 0.017 .0056082 .0553022

6 | -.1070091 .0133164 -8.04 0.000 -.1337559 -.0802623

8 | -.014567 .0127621 -1.14 0.259 -.0402005 .0110664

9 | .110917 .0124929 8.88 0.000 .0858243 .1360097

10 | .0192278 .0156837 1.23 0.226 -.0122738 .0507294

11 | .0334975 .0138687 2.42 0.019 .0056414 .0613536

12 | .0283076 .0105211 2.69 0.010 .0071754 .0494398

13 | -.02229 .0109857 -2.03 0.048 -.0443555 -.0002245

15 | .0166339 .0196375 0.85 0.401 -.0228092 .0560771

16 | .0154175 .0098178 1.57 0.123 -.0043022 .0351372

17 | .0046427 .0130131 0.36 0.723 -.0214949 .0307803

18 | .0179066 .0120056 1.49 0.142 -.0062073 .0420205

19 | .1289816 .01413 9.13 0.000 .1006007 .1573625

20 | .0505663 .0079863 6.33 0.000 .0345253 .0666074

21 | .0517243 .0129467 4.00 0.000 .0257201 .0777286

22 | -.0233817 .0085157 -2.75 0.008 -.0404859 -.0062774

23 | .1499863 .0104745 14.32 0.000 .1289476 .1710249

24 | -.0258664 .0125576 -2.06 0.045 -.0510891 -.0006436

25 | .0670315 .0127416 5.26 0.000 .0414394 .0926237

26 | .1134835 .0136261 8.33 0.000 .0861146 .1408525

27 | .0584283 .0137375 4.25 0.000 .0308356 .0860209

28 | .0055399 .0022871 2.42 0.019 .0009462 .0101337

29 | .0573427 .009823 5.84 0.000 .0376126 .0770727

30 | .0571815 .0154541 3.70 0.001 .0261411 .0882219

31 | .0174791 .0134112 1.30 0.198 -.0094581 .0444162

32 | -.055743 .0142684 -3.91 0.000 -.0844019 -.027084

33 | .0319857 .0153523 2.08 0.042 .0011496 .0628218

34 | -.0211717 .0131528 -1.61 0.114 -.0475899 .0052464

35 | .0061231 .0129441 0.47 0.638 -.0198759 .0321221

36 | .0559758 .0124085 4.51 0.000 .0310527 .080899

37 | .0232193 .0059777 3.88 0.000 .0112127 .0352259

38 | .0297997 .017834 1.67 0.101 -.0060209 .0656203

39 | .0875127 .0130242 6.72 0.000 .0613528 .1136726

40 | .0401661 .0110057 3.65 0.001 .0180605 .0622718

41 | .1571103 .0129029 12.18 0.000 .131194 .1830265

42 | .0911957 .0117966 7.73 0.000 .0675016 .1148898

44 | .1387425 .0108093 12.84 0.000 .1170314 .1604535

45 | .033432 .001053 31.75 0.000 .031317 .035547

46 | .1067252 .015413 6.92 0.000 .0757672 .1376832

47 | .083409 .0074792 11.15 0.000 .0683865 .0984315

48 | .0059254 .0153378 0.39 0.701 -.0248814 .0367323

49 | .0063227 .0128685 0.49 0.625 -.0195245 .0321698

50 | .0817028 .0175396 4.66 0.000 .0464734 .1169322

51 | -.0195869 .0110414 -1.77 0.082 -.0417643 .0025905

53 | .0887817 .013082 6.79 0.000 .0625056 .1150577

54 | .1411377 .013405 10.53 0.000 .1142131 .1680624

55 | .1027432 .0099465 10.33 0.000 .0827652 .1227213

56 | -.0099932 .0093507 -1.07 0.290 -.0287747 .0087882

|

male | -.0215756 .003721 -5.80 0.000 -.0290495 -.0141017

age | .0157436 .0015672 10.05 0.000 .0125959 .0188914

age2 | -.0001479 .0000163 -9.10 0.000 -.0001806 -.0001153

race_w | -.0863861 .0157263 -5.49 0.000 -.1179732 -.054799

race_b | .0145335 .0182424 0.80 0.429 -.0221075 .0511744

race_n | -.0203663 .0344213 -0.59 0.557 -.0895036 .048771

race_a | -.1131491 .0187599 -6.03 0.000 -.1508295 -.0754686

race_p | -.0128189 .0469682 -0.27 0.786 -.1071574 .0815196

latino | -.0049634 .0188113 -0.26 0.793 -.0427471 .0328203

married | -.1070529 .0095234 -11.24 0.000 -.1261813 -.0879245

famsize | .0151384 .0051865 2.92 0.005 .004721 .0255559

fpl_pct | .0039769 .0005013 7.93 0.000 .00297 .0049838

fpl_pct2 | -.0000323 3.50e-06 -9.23 0.000 -.0000393 -.0000253

educ_1 | .2240969 .0177531 12.62 0.000 .1884387 .2597551

educ_2 | .129838 .0093784 13.84 0.000 .111001 .1486751

educ_3 | .0900188 .0088804 10.14 0.000 .072182 .1078556

unempl | .0054035 .0030693 1.76 0.084 -.0007614 .0115684

abawdwaive | .0232751 .0114497 2.03 0.047 .0002777 .0462724

_cons | -.2900216 .0595787 -4.87 0.000 -.4096888 -.1703543

------------------------------------------------------------------------------

. lincom joint x expansion + medicaid expansion

( 1) joint x expansion + medicaid expansion = 0

------------------------------------------------------------------------------

snap | Coef. Std. Err. t P>|t| [95% Conf. Interval]

-------------+----------------------------------------------------------------

(1) | .0361196 .0128488 2.81 0.007 .0103121 .0619272

------------------------------------------------------------------------------

| **ACA Medicaid Expansion and Healthcare Navigator Grants: Effects on SNAP Food Assistance Receipt** | | |
| --- | --- | --- |
|  |  |  |
|  | **Ordinary**  **Least-Squares** | **Difference-in-Differences** |
| Navigator Grant | -0.001 | 0.003 |
| Robust standard error | (0.003) | (0.003) |
| P-value | 0.842 | 0.417 |
| Effect of Medicaid Expansion |  | 0.042*** |
| Robust standard error |  | (0.009) |
| P-value |  | <0.001 |
| Navigator Grant x Expansion |  | -0.006 |
| Robust standard error |  | (0.004) |
| P-value |  | 0.129 |
| N | 224706 | 413893 |
| Mean of dependent variable | 0.402 | 0.416 |

***p< 0.001 Data source is the Current Population Survey ASEC, 2011-2020 (reporting periods 2010-2019) for differences-and-differences. Data from 2015-2020 (reporting periods 2014-2019) are used for ordinary least-squares analyses of navigator grants, which began in 2014. Navigator grants are in dollars, scaled by low-income population size of each recipient state. All 50 states and DC are included. Regressions are linear probability models using survey weights. The unit of analysis is the individual. Standard errors in parentheses are robust to heteroskedasticity and clustered by state.

**Ordinary least-squares full regression output**

Linear regression Number of obs = 224,706

F(24, 50) = .

Prob > F = .

R-squared = 0.1174

Root MSE = .46064

(Std. Err. adjusted for 51 clusters in statefip)

------------------------------------------------------------------------------

| Robust

snap | Coef. Std. Err. t P>|t| [95% Conf. Interval]

-------------+----------------------------------------------------------------

navigator grant | -.0006235 .0031131 -0.20 0.842 -.0068763 .0056294

|

year |

2015 | .0104086 .0102687 1.01 0.316 -.0102168 .0310339

2016 | .0115357 .0094963 1.21 0.230 -.0075381 .0306095

2017 | -.0259392 .012083 -2.15 0.037 -.0502086 -.0016697

2018 | .0004815 .0141872 0.03 0.973 -.0280144 .0289774

2019 | -.0189912 .0194825 -0.97 0.334 -.0581229 .0201405

|

statefip |

2 | -.1169453 .0302022 -3.87 0.000 -.1776081 -.0562824

4 | -.00211 .011323 -0.19 0.853 -.0248529 .0206328

5 | -.0057736 .0072373 -0.80 0.429 -.0203102 .008763

6 | -.0963231 .0103218 -9.33 0.000 -.1170551 -.075591

8 | -.038403 .0150497 -2.55 0.014 -.0686312 -.0081748

9 | .0218294 .0095721 2.28 0.027 .0026033 .0410555

10 | -.0030391 .0153605 -0.20 0.844 -.0338915 .0278132

11 | -.0374983 .0082131 -4.57 0.000 -.0539949 -.0210017

12 | -.0109458 .0072656 -1.51 0.138 -.0255392 .0036476

13 | -.0802028 .0085145 -9.42 0.000 -.0973047 -.0631009

15 | -.0000982 .0204605 -0.00 0.996 -.0411944 .040998

16 | -.0051517 .0152946 -0.34 0.738 -.0358717 .0255684

17 | -.0055867 .008692 -0.64 0.523 -.0230451 .0118718

18 | -.0178255 .006315 -2.82 0.007 -.0305095 -.0051414

19 | .0481919 .0131844 3.66 0.001 .0217103 .0746735

20 | -.0307138 .00834 -3.68 0.001 -.0474651 -.0139624

21 | .0060266 .008938 0.67 0.503 -.0119258 .0239791

22 | -.0429069 .0070738 -6.07 0.000 -.0571151 -.0286987

23 | .1106485 .0080386 13.76 0.000 .0945024 .1267945

24 | -.0493264 .012368 -3.99 0.000 -.0741682 -.0244846

25 | .0371235 .0120047 3.09 0.003 .0130114 .0612356

26 | .0323409 .0074834 4.32 0.000 .0173101 .0473717

27 | .0201553 .0159357 1.26 0.212 -.0118526 .0521631

28 | -.0285619 .0043786 -6.52 0.000 -.0373566 -.0197673

29 | .0085272 .0053727 1.59 0.119 -.0022642 .0193185

30 | .0311907 .0087604 3.56 0.001 .0135948 .0487866

31 | .0233441 .0135579 1.72 0.091 -.0038877 .0505759

32 | -.084252 .0089766 -9.39 0.000 -.102282 -.066222

33 | .011343 .0151701 0.75 0.458 -.019127 .041813

34 | -.0596814 .0052247 -11.42 0.000 -.0701755 -.0491873

35 | .0379803 .0101604 3.74 0.000 .0175725 .058388

36 | .0131382 .0080479 1.63 0.109 -.0030266 .0293029

37 | .005295 .0044713 1.18 0.242 -.0036858 .0142759

38 | -.0576798 .0185384 -3.11 0.003 -.0949154 -.0204442

39 | .065791 .005406 12.17 0.000 .0549328 .0766491

40 | -.0307925 .006749 -4.56 0.000 -.0443483 -.0172366

41 | .096049 .0110108 8.72 0.000 .0739332 .1181648

42 | .0230571 .0072439 3.18 0.003 .0085073 .037607

44 | .0899209 .0095402 9.43 0.000 .0707589 .109083

45 | -.0112182 .0024637 -4.55 0.000 -.0161667 -.0062697

46 | .0757619 .0158955 4.77 0.000 .0438349 .1076889

47 | .0341885 .0065064 5.25 0.000 .0211201 .0472569

48 | -.0334031 .0071408 -4.68 0.000 -.0477458 -.0190604

49 | -.0461188 .0107565 -4.29 0.000 -.0677239 -.0245138

50 | .0642401 .0185163 3.47 0.001 .0270489 .1014312

51 | -.0486686 .0069111 -7.04 0.000 -.0625499 -.0347873

53 | .0292166 .0101467 2.88 0.006 .0088365 .0495968

54 | .0914919 .0076759 11.92 0.000 .0760745 .1069093

55 | .0492024 .0091045 5.40 0.000 .0309156 .0674892

56 | -.0640111 .0256223 -2.50 0.016 -.1154749 -.0125473

|

male | -.02394 .0020352 -11.76 0.000 -.0280278 -.0198522

age | -.0002774 .0002828 -0.98 0.331 -.0008455 .0002906

age2 | 5.90e-06 5.16e-06 1.14 0.259 -4.47e-06 .0000163

race_w | -.0977399 .015694 -6.23 0.000 -.1292621 -.0662176

race_b | .0272106 .0152175 1.79 0.080 -.0033546 .0577758

race_n | -.0277996 .0256265 -1.08 0.283 -.079272 .0236728

race_a | -.1435361 .0148469 -9.67 0.000 -.173357 -.1137152

race_p | -.0631897 .0327981 -1.93 0.060 -.1290667 .0026873

latino | -.0170017 .0121034 -1.40 0.166 -.0413122 .0073087

married | -.0539477 .0051731 -10.43 0.000 -.0643381 -.0435573

famsize | -.0224648 .0030133 -7.46 0.000 -.0285171 -.0164125

fpl_pct | .0043524 .0001847 23.57 0.000 .0039814 .0047234

fpl_pct2 | -.0000398 1.38e-06 -28.87 0.000 -.0000426 -.000037

hhkids | .0731011 .0054237 13.48 0.000 .0622072 .083995

educ_1 | .1995607 .0109786 18.18 0.000 .1775096 .2216118

educ_2 | .1518848 .0118136 12.86 0.000 .1281564 .1756132

educ_3 | .1161715 .010121 11.48 0.000 .0958428 .1365002

abawdwaive | .0130845 .01146 1.14 0.259 -.0099336 .0361027

unempl | .0184284 .0065674 2.81 0.007 .0052375 .0316193

_cons | .2065794 .0497158 4.16 0.000 .1067224 .3064365

------------------------------------------------------------------------------

**Difference-in-differences full regression output**

Linear regression Number of obs = 413,893

F(30, 50) = .

Prob > F = .

R-squared = 0.1251

Root MSE = .46112

(Std. Err. adjusted for 51 clusters in statefip)

------------------------------------------------------------------------------

| Robust

snap | Coef. Std. Err. t P>|t| [95% Conf. Interval]

-------------+----------------------------------------------------------------

medicaid expansion | .0421268 .0092779 4.54 0.000 .0234916 .0607619

grant x expansion | -.0062164 .0040298 -1.54 0.129 -.0143104 .0018777

navigator grant| .002786 .0034063 0.82 0.417 -.0040557 .0096276

|

year |

2011 | .0338428 .0090272 3.75 0.000 .0157111 .0519744

2012 | .044077 .0101462 4.34 0.000 .0236978 .0644561

2013 | .066677 .0112346 5.93 0.000 .0441117 .0892423

2014 | .0403651 .0125652 3.21 0.002 .0151272 .065603

2015 | .0367143 .0169478 2.17 0.035 .0026737 .0707548

2016 | .0295395 .0171183 1.73 0.091 -.0048437 .0639226

2017 | -.013747 .0184525 -0.74 0.460 -.0508099 .0233159

2018 | .0069314 .0206004 0.34 0.738 -.0344458 .0483085

2019 | -.0133138 .0177684 -0.75 0.457 -.0490027 .0223751

|

statefip |

2 | -.1184251 .0133212 -8.89 0.000 -.1451814 -.0916687

4 | -.0397707 .0101194 -3.93 0.000 -.0600962 -.0194453

5 | -.0201213 .006043 -3.33 0.002 -.0322591 -.0079836

6 | -.1323328 .008569 -15.44 0.000 -.1495441 -.1151214

8 | -.0761987 .0087689 -8.69 0.000 -.0938115 -.0585858

9 | -.0172262 .006467 -2.66 0.010 -.0302156 -.0042368

10 | -.0217395 .0134456 -1.62 0.112 -.0487458 .0052668

11 | -.0502616 .0055368 -9.08 0.000 -.0613827 -.0391406

12 | -.0255146 .0052326 -4.88 0.000 -.0360246 -.0150047

13 | -.0907707 .0030508 -29.75 0.000 -.0968984 -.084643

15 | -.0617019 .0139097 -4.44 0.000 -.0896404 -.0337634

16 | -.0114932 .008618 -1.33 0.188 -.0288029 .0058165

17 | -.0249157 .0063608 -3.92 0.000 -.0376917 -.0121398

18 | -.029001 .0050229 -5.77 0.000 -.0390898 -.0189123

19 | .0195793 .0117928 1.66 0.103 -.0041072 .0432658

20 | -.0365237 .007517 -4.86 0.000 -.051622 -.0214254

21 | .0036519 .0046066 0.79 0.432 -.0056007 .0129044

22 | -.0629327 .0042657 -14.75 0.000 -.0715006 -.0543648

23 | .1005559 .0056353 17.84 0.000 .0892371 .1118747

24 | -.0905304 .0071331 -12.69 0.000 -.1048577 -.0762032

25 | .0016766 .0077658 0.22 0.830 -.0139215 .0172747

26 | .0374563 .0046453 8.06 0.000 .028126 .0467866

27 | -.0280553 .0095634 -2.93 0.005 -.0472639 -.0088467

28 | -.0026957 .002526 -1.07 0.291 -.0077693 .0023779

29 | -.0147721 .003455 -4.28 0.000 -.0217116 -.0078325

30 | -.0105322 .0093894 -1.12 0.267 -.0293915 .008327

31 | -.0376416 .011505 -3.27 0.002 -.0607501 -.0145331

32 | -.1444221 .0078904 -18.30 0.000 -.1602705 -.1285738

33 | -.0542463 .0167929 -3.23 0.002 -.0879757 -.0205168

34 | -.1028294 .0080042 -12.85 0.000 -.1189063 -.0867524

35 | -.000078 .0093254 -0.01 0.993 -.0188086 .0186527

36 | -.0176384 .0062672 -2.81 0.007 -.0302264 -.0050503

37 | -.0140445 .0023179 -6.06 0.000 -.0187003 -.0093888

38 | -.0660101 .021987 -3.00 0.004 -.1101722 -.021848

39 | .028842 .0053144 5.43 0.000 .0181676 .0395163

40 | -.0334031 .0071374 -4.68 0.000 -.0477391 -.0190671

41 | .0652341 .0059248 11.01 0.000 .0533339 .0771343

42 | -.0155036 .0060681 -2.55 0.014 -.0276917 -.0033155

44 | .0633819 .0063117 10.04 0.000 .0507045 .0760592

45 | -.0158904 .0009849 -16.13 0.000 -.0178687 -.0139121

46 | .024248 .0130096 1.86 0.068 -.0018826 .0503787

47 | .0328047 .0023128 14.18 0.000 .0281593 .03745

48 | -.0385115 .0081241 -4.74 0.000 -.0548293 -.0221936

49 | -.1016563 .009443 -10.77 0.000 -.120623 -.0826896

50 | .0313527 .0110358 2.84 0.006 .0091866 .0535187

51 | -.0870652 .0062609 -13.91 0.000 -.0996405 -.0744899

53 | .0032097 .0061055 0.53 0.601 -.0090537 .015473

54 | .0715488 .0060349 11.86 0.000 .0594273 .0836702

55 | .0393774 .0051663 7.62 0.000 .0290005 .0497543

56 | -.0987896 .0158791 -6.22 0.000 -.1306837 -.0668954

|

male | -.0231548 .0016959 -13.65 0.000 -.026561 -.0197486

age | -.0007019 .0002384 -2.94 0.005 -.0011808 -.0002231

age2 | 6.02e-06 5.02e-06 1.20 0.237 -4.07e-06 .0000161

race_w | -.0861652 .0104329 -8.26 0.000 -.1071202 -.0652102

race_b | .0396379 .011396 3.48 0.001 .0167482 .0625275

race_n | -.0198976 .016505 -1.21 0.234 -.0530489 .0132538

race_a | -.1381404 .0147518 -9.36 0.000 -.1677703 -.1085105

race_p | -.0482804 .0358613 -1.35 0.184 -.1203098 .0237491

latino | -.0127573 .0138956 -0.92 0.363 -.0406674 .0151529

married | -.0392953 .0054362 -7.23 0.000 -.0502143 -.0283763

famsize | -.0238308 .0025791 -9.24 0.000 -.029011 -.0186507

fpl_pct | .0042312 .0001887 22.43 0.000 .0038523 .0046102

fpl_pct2 | -.00004 1.33e-06 -30.02 0.000 -.0000427 -.0000373

hhkids | .0742219 .0045122 16.45 0.000 .0651588 .0832849

educ_1 | .217848 .0103048 21.14 0.000 .1971501 .2385458

educ_2 | .1652867 .0093835 17.61 0.000 .1464393 .184134

educ_3 | .1298569 .0089939 14.44 0.000 .1117922 .1479216

abawdwaive | .014483 .0064157 2.26 0.028 .0015967 .0273693

unempl | .0046234 .0030728 1.50 0.139 -.0015486 .0107953

_cons | .2480328 .0347741 7.13 0.000 .1781871 .3178786

------------------------------------------------------------------------------
